# Prevalence of gastrointestinal side effects in hepatocellular carcinoma patients receiving sorafenib: a meta-analysis of 136 studies and 14,416 patients

**DOI:** 10.1177/17588359261442686

**Published:** 2026-05-13

**Authors:** Nathalie Arendt, Tania Payo-Serafín, Anna Kolosenko, Maria Kopsida, Markus Sjöblom, Femke Heindryckx

**Affiliations:** Department of Medical Cell Biology, Uppsala University, Uppsala, Sweden; Institute of Biomedicine, Universidad de León, León, Spain; Department of Medical Cell Biology, Uppsala University, Uppsala, Sweden; Department of Medical Cell Biology, Uppsala University, Uppsala, Sweden; Department of Medical Cell Biology, Uppsala University, Uppsala, Sweden; Department of Medical Cell Biology, Biomedical Center, Uppsala University, Husargatan 3, Uppsala 75 123, Sweden

**Keywords:** abdominal pain, diarrhea, dose modification, gastrointestinal adverse events, hepatocellular carcinoma, nausea, sorafenib, toxicity

## Abstract

**Background:**

Sorafenib, an oral multikinase inhibitor, was the first systemic therapy to demonstrate an overall survival benefit in advanced hepatocellular carcinoma (HCC). Gastrointestinal (GI) adverse events (AE), particularly diarrhea, nausea, and abdominal pain, are commonly reported toxicities and often drive dose modifications.

**Objectives:**

To map the prevalence of GI AE in patients with HCC receiving sorafenib.

**Design:**

Systematic review and meta-analysis conducted in accordance with Preferred Reporting Items for Systematic Reviews and Meta-Analyses guidelines.

**Data sources and methods:**

Clinical trials and relevant observational studies from PubMed reporting on GI side effects with sorafenib were included. Exclusion criteria were studies not involving Sorafenib, studies not involving HCC, previous meta-analyses or systematic reviews, and inaccessible publications. Prevalence of reported GI complications, including diarrhea, nausea, GI toxicity, weight loss/anorexia, constipation, and abdominal pain, were extracted from the included studies and analyzed using GraphPad Prism.

**Results:**

The analysis included 136 studies (178 study divisions; total 14,416 patients). Diarrhea was reported in 132 studies, with a mean prevalence of 40.44% (95% CI: 37.31–43.58) and a weighted prevalence of 42.21%. Nausea was reported in 92 studies (mean 24.75%, 95% CI: 21.19–28.28; weighted 20.35%). Abdominal pain was reported in 62 studies (mean 25.85%, 95% CI: 21.52–30.18; weighted 22.30%). GI toxicity was reported in 69 studies (mean 16.96%, 95% CI: 13.07–20.85; weighted 16.11%). Weight loss and anorexia was reported in 66 studies (mean 24.79%, 95% CI: 20.58–29.00; weighted 22.67%). Constipation was reported in 33 studies (mean 16.93%, 95% CI: 13.97–19.89; weighted 14.13%).

**Conclusion:**

This meta-analysis synthesizes current evidence on GI AEs with sorafenib in HCC, highlighting diarrhea as the most frequent toxicity and underscoring the need for standardized AE reporting and proactive management strategies to maintain adherence and outcomes.

## Plain language summary

When liver cancer therapy hits the gut: a global look at sorafenib’s side effects

Sorafenib is a used to treat people with advanced liver cancer (hepatocellular carcinoma, HCC). It was the first treatment shown to help patients with liver cancer live longer. However, many people taking sorafenib experience stomach and gut-related side effects such as diarrhea, nausea, and stomach pain. These side effects can be serious enough to require lowering the dose or stopping treatment. In this study, we looked at how common these side effects are by reviewing results from 136 clinical studies that together included more than 14,000 patients treated with sorafenib. We found that diarrhea was the most commonly reported GI-related side effect, affecting about 4 out of 10 patients. Both nausea and abdominal pain occurred in about 1 in 4 patients, while weight loss affected about 1 in 5 patients. Constipation and general gut-related toxicity were less common, affecting about 1 in 6 patients. These results show that stomach and gut problems, especially diarrhea, are frequent in people treated with sorafenib. Better ways to monitor and manage these side effects are needed to help patients continue treatment and get the most benefit from it.

## Introduction

Sorafenib, an oral multikinase inhibitor, was the first systemic therapy to demonstrate an overall survival benefit in patients with advanced hepatocellular carcinoma (HCC) and subsequently established itself as the global standard of care for over a decade.^1^ By targeting multiple signaling pathways involved in tumor proliferation and angiogenesis, including RAF, vascular endothelial growth factor receptor (VEGFR), and platelet-derived growth factor receptors (PDGFR), sorafenib slows tumor growth and progression.^2^ Unlike locoregional therapies such as transarterial chemoembolization (TACE), sorafenib acts systemically, which contributes to a distinct and broader spectrum of adverse events (AEs).^3^

Among these common toxicities are upper and lower gastrointestinal (GI) disturbances, which can substantially impact treatment adherence and quality of life.^4–7^ These symptoms include diarrhea, nausea, vomiting, abdominal pain,^6,8^ and in some cases gastrointestinal bleeding or mucositis.^9–11^ These AEs range from mild and self-limiting to severe, often requiring dose reduction or treatment discontinuation.^3–5,7^ Diarrhea is particularly notable, frequently reported as a GI side effect and a common reason for sorafenib dose modification.^6,12^

The mechanisms behind sorafenib-related GI toxicity are likely multifactorial.^8,13^ Inhibition of VEGFRs and PDGFRs can disrupt angiogenesis and reduce mucosal perfusion, impairing epithelial repair and promoting ischemic or inflammatory injury to the gastrointestinal lining. Inhibition of RAF kinases may also interfere with epithelial cell proliferation and turnover, further compromising mucosal barrier function. In addition, VEGF pathway inhibition has been implicated in altered intestinal permeability and dysregulated fluid transport, which may contribute to diarrhea and abdominal discomfort observed in treated patients.^14^ These mechanisms likely act concurrently, leading to impaired absorption, increased luminal secretion, and heightened susceptibility to mucosal injury. Emerging evidence also suggests that tyrosine kinase inhibitors, including sorafenib, may induce alterations in the gut microbiome, potentially exacerbating mucosal inflammation and gastrointestinal symptoms through dysbiosis-related immune and metabolic effects.^8,15^ Nausea and abdominal pain may reflect a combination of systemic drug exposure, local mucosal irritation, and treatment-related metabolic disturbances.^11,16^ Patient-specific factors, including baseline hepatic function, comorbid conditions, and prior therapeutic exposures may further increase susceptibility to these AEs.^8^ Despite their frequency and clinical importance, gastrointestinal complications associated with cancer therapies are inconsistently reported across studies, resulting in substantial heterogeneity in outcome assessment.^11^ This heterogeneity limits cross-study comparability and complicates the development of standardized management strategies.

The aim of this meta-analysis is to comprehensively map the prevalence of gastrointestinal complications in HCC patients treated with sorafenib, focusing on diarrhea, nausea, abdominal pain, weight loss, constipation, and GI toxicity (including mucositis, intestinal bleeding, and ulcerations). Although gastrointestinal toxicities associated with sorafenib are well recognized and summarized in the prescribing information, these data are largely derived from individual clinical trials with heterogeneous designs, patient populations, and reporting standards. The rationale for the present meta-analysis was not to establish the existence of these AEs, rather to provide a systematic, pooled quantification of their incidence across diverse study settings, thereby offering more precise and generalizable estimates than those available from single trials. Pooled estimates are particularly relevant given evolving treatment paradigms, differences between trial populations and real-world practice, and variability in the reporting of gastrointestinal AEs across studies.

Importantly, quantifying the prevalence and distribution of gastrointestinal toxicities at the meta-analytic level has implications beyond routine clinical awareness. This data can inform anticipatory counseling, risk–benefit discussions, and the design of monitoring and supportive care strategies, particularly for patients at higher risk of treatment intolerance. From a research perspective, the findings highlight inconsistencies in AE reporting and underscore the need for standardized toxicity endpoints and time-to-onset analyses in future trials. By synthesizing available evidence in this way, this work seeks to provide a clearer understanding of the burden of these side effects, highlight gaps in knowledge, and inform clinical practice to improve patient management and outcomes.

## Methods

### Eligibility criteria

**Figure 1.**
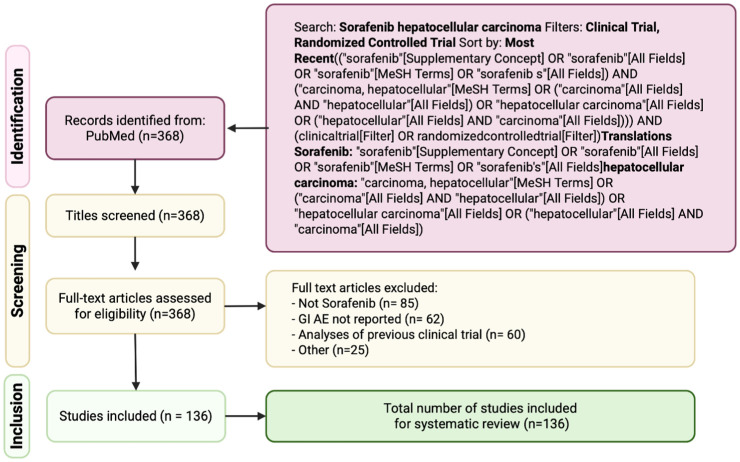
PRISMA diagram illustrating the progression of information through the phases of the systematic review. PRISMA, Preferred Reporting Items for Systematic Review and Meta-Analyses.

Studies were considered eligible if they reported gastrointestinal AEs in patients with HCC treated with sorafenib, specifically diarrhea, nausea, abdominal pain, anorexia/weight loss, constipation, or gastrointestinal toxicities (mucositis, ulceration, hemorrhage). Only clinical trials (including both nonrandomized and randomized controlled trials (RCTs)), and prospective or retrospective studies were included. Exclusion criteria were absence of GI AE reporting, nonsorafenib studies, meta-analyses, systematic reviews, case reports, secondary analyses of prior trials, and inaccessible publications. The Preferred Reporting Items for Systematic Reviews and Meta-Analyses (PRISMA) guidelines were followed (Figure 1, Supplemental Table 1). The review protocol was not registered.

### Information sources and search strategy

The literature search was performed in PubMed using the terms “sorafenib” and “hepatocellular carcinoma,” limited to clinical trials and relevant observational studies. Data extraction was conducted by six independent reviewers, with each entry verified by two others; therefore, all data points were independently assessed by three reviewers. All methodological procedures were predefined prior to data collection.

### Data collection

Data extraction included study identifier (PMID), author, publication year, and study design, as well as geographical location. Participant demographics included sample size and median age. Treatment variables recorded were sorafenib dose, treatment duration, and the occurrence of dose reductions or interruptions. The primary outcomes were the prevalence of gastrointestinal AEs—diarrhea, nausea, abdominal pain, anorexia/weight loss, constipation, and gastrointestinal toxicities (mucositis, ulceration, gastrointestinal hemorrhage)—reported as percentages. AEs not assessed or reported in a study were coded as missing rather than zero and excluded from prevalence estimates to minimize bias. Study quality and risk of bias were assessed using the Mixed Methods Appraisal Tool (MMAT).^17^ Following the MMAT framework, studies were first classified by study design (RCTs, nonrandomized quantitative studies, or descriptive quantitative studies) and then appraised using the five design-specific MMAT criteria. Assessments were performed independently by three reviewers, with each MMAT criterion rated as “Yes,” “No,” or “Can’t tell,” and discrepancies resolved by majority decision (2/3 agreement). Results are presented as criterion-level ratings in accordance with MMAT standards (Supplemental Table 2), without calculating an overall numeric score.

### Statistical analyses

Data were managed in Microsoft Excel and imported into GraphPad Prism version 10.2.3 for analysis. Prevalence of each gastrointestinal AE was summarized across study arms using both mean prevalence (±standard error of the mean (SEM)) and weighted prevalence based on sample size. Bar charts were plotted to show mean prevalences values within the included studies, with error bars indicating SEM. To explore potential causes of variability among study results, subgroup analyses were conducted (e.g., by sorafenib dose, treatment setting (monotherapy vs combination), and type of combination regimen). Unpaired Student’s *t*-tests were used to assess between-group comparisons and a two-sided *p* value < 0.05 was considered statistically significant. Principal component analysis (PCA) was performed on standardized study-level variables (*z*-scored; mean = 0, SD = 1) to explore clustering patterns. Components were retained using the Kaiser criterion (eigenvalues > 1). No variance-based fixed- or random-effects meta-analysis model was applied.

## Results

### Study selection

A systematic PubMed search was performed to identify studies reporting GI AEs in patients with HCC treated with sorafenib. The search strategy combined keywords and MeSH terms for “sorafenib” and “hepatocellular carcinoma,” covering publications from 2005 to 2025. Filters were applied to include clinical trials and RCTs, while excluding systematic reviews, meta-analyses, study protocols, and case reports.

The initial search identified 368 records (Supplemental Table 3). All titles and abstracts were screened according to the predefined inclusion and exclusion criteria. Studies were excluded if they were unrelated to sorafenib treatment, did not focus on HCC, or lacked data on gastrointestinal AEs. Following full-text review, 85 studies were excluded for not involving sorafenib, 62 for not reporting GI AEs, 60 as duplicate analyses of previously published trials, and 25 for other reasons, including inaccessibility, language limitations, or ineligible study design (Supplemental Table 4). A total of 136 studies met inclusion criteria and were retained for final analysis (Table 1 and Supplemental Table 5). Study quality was evaluated using the MMAT tool, with detailed ratings presented in Supplemental Table 2.

**Table 1.** Included studies.

ID	Authors	Title	Year	Reference
1	Fan et al.	Survival in patients with recurrent intermediate-stage hepatocellular carcinoma: sorafenib plus TACE vs TACE alone randomized clinical trial	2024	18
2	Peng et al.	Adjuvant transarterial chemoembolization with sorafenib for portal vein tumor thrombus: a randomized clinical trial	2024	19
3	Yau et al.	Cabozantinib plus atezolizumab versus sorafenib for advanced hepatocellular carcinoma (COSMIC-312): final results of a randomised phase 3 study	2024	20
4	Qin et al.	Tislelizumab vs sorafenib as first-line treatment for unresectable hepatocellular carcinoma: a phase 3 randomized clinical trial	2023	21
5	Qin et al	Camrelizumab plus rivoceranib versus sorafenib as first-line therapy for unresectable hepatocellular carcinoma (CARES-310): a randomized, open-label, international phase 3 study	2023	22
6	Okusaka et al.	A phase I study to investigate the safety, tolerability, and pharmacokinetics of napabucasin combined with sorafenib in Japanese patients with unresectable hepatocellular carcinoma	2023	23
7	Lu et al.	Irradiation stent with 125 I plus TACE versus sorafenib plus TACE for hepatocellular carcinoma with major portal vein tumor thrombosis: a multicenter randomized trial	2023	24
8	Abou-Alfa et al.	Tremelimumab plus durvalumab in unresectable hepatocellular carcinoma	2022	25
9	Kelley et al.	Cabozantinib plus atezolizumab versus sorafenib for advanced hepatocellular carcinoma (COSMIC-312): a multicentre, open-label, randomised, phase 3 trial	2022	26
10	Zheng et al.	Sorafenib plus hepatic arterial infusion chemotherapy versus sorafenib for hepatocellular carcinoma with major portal vein tumor thrombosis: a randomized trial	2022	27
11	Chen et al.	GALNT14 genotype-guided chemoembolization plus sorafenib therapy in hepatocellular carcinoma: a randomized trial	2022	28
12	Abou-Alfa et al.	Phase II study of sorafenib in patients with advanced hepatocellular carcinoma	2006	29
13	Furuse et al.	Phase I study of sorafenib in Japanese patients with hepatocellular carcinoma	2008	30
14	Llovet et al.	Sorafenib in advanced hepatocellular carcinoma	2008	31
15	Cheng et al.	Efficacy and safety of sorafenib in patients in the Asia-Pacific region with advanced hepatocellular carcinoma: a Phase III randomised, double-blind, placebo-controlled trial	2009	32
16	Richly et al.	Combination of sorafenib and doxorubicin in patients with advanced hepatocellular carcinoma: results from a Phase I extension trial	2009	33
17	Yau et al.	Phase 2 open-label study of single-agent sorafenib in treating advanced hepatocellular carcinoma in a hepatitis B-endemic Asian population: presence of lung metastasis predicts poor response	2009	34
18	Yau et al.	Nivolumab versus sorafenib in advanced hepatocellular carcinoma (CheckMate 459): a randomised, multicentre, open-label, Phase 3 trial	2022	35
19	Lyu et al.	Arterial chemotherapy of oxaliplatin plus fluorouracil versus sorafenib in advanced hepatocellular carcinoma: a biomolecular exploratory, randomized, Phase III trial (FOHAIC-1)	2022	36
20	Hashimoto et al.	Upregulation of C/EBPα inhibits suppressive activity of myeloid cells and potentiates antitumor response in mice and patients with cancer	2021	37
21	Bockorny et al.	Priming of sorafenib prior to radiofrequency ablation does not increase treatment effect in hepatocellular carcinoma	2021	38
22	Ding et al.	Transarterial chemoembolization plus lenvatinib versus transarterial chemoembolization plus sorafenib as first-line treatment for hepatocellular carcinoma with portal vein tumor thrombus: a prospective randomized study	2021	39
23	Kaibori et al.	The impact of sorafenib in combination with intermittent hepatic arterial infusion chemotherapy for unresectable hepatocellular carcinoma with major vascular invasion	2022	40
24	Qin et al.	Donafenib versus sorafenib in first-line treatment of unresectable or metastatic hepatocellular carcinoma: a randomized, open-label, parallel-controlled Phases II–III trial	2021	41
25	Ren et al.	Sintilimab plus a bevacizumab biosimilar (IBI305) versus sorafenib in unresectable hepatocellular carcinoma (ORIENT-32): a randomised, open-label, Phases 2–3 study	2021	42
26	Ryoo et al.	Randomised Phase 1b/2 trial of tepotinib vs sorafenib in Asian patients with advanced hepatocellular carcinoma with MET overexpression	2021	43
27	Lin et al.	Potential of novel colchicine dosage schedule for the palliative treatment of advanced hepatocellular carcinoma	2021	44
28	Haruna et al.	Efficacy and safety of sorafenib plus vitamin K treatment for hepatocellular carcinoma: a Phase II, randomized study	2021	45
29	El Dika et al.	Phase II trial of sorafenib and doxorubicin in patients with advanced hepatocellular carcinoma after disease progression on sorafenib	2020	46
30	Kim et al.	A Phase I trial of trametinib in combination with sorafenib in patients with advanced hepatocellular cancer	2020	47
31	Harding et al.	Phase Ib study of enzalutamide with or without sorafenib in patients with advanced hepatocellular carcinoma	2020	48
32	Finn et al.	Atezolizumab plus bevacizumab in unresectable hepatocellular carcinoma	2020	49
33	He et al.	Sorafenib plus hepatic arterial infusion of oxaliplatin, fluorouracil, and leucovorin vs sorafenib alone for hepatocellular carcinoma with portal vein invasion: a randomized clinical trial	2019	50
34	Assenat et al.	Sorafenib alone vs. sorafenib plus GEMOX as 1(st)-line treatment for advanced HCC: the Phase II randomised PRODIGE 10 trial	2019	51
35	Naganuma et al.	β-Hydroxy-β-methyl butyrate/L-arginine/L-glutamine supplementation for preventing hand-foot skin reaction in sorafenib for advanced hepatocellular carcinoma	2019	52
36	Park et al.	Sorafenib with or without concurrent transarterial chemoembolization in patients with advanced hepatocellular carcinoma: the Phase III STAH trial	2019	53
37	Tak et al.	Phase I/II study of first-line combination therapy with sorafenib plus resminostat, an oral HDAC inhibitor, versus sorafenib monotherapy for advanced hepatocellular carcinoma in east Asian patients	2018	54
38	Goyal et al.	A Phase II and biomarker study of sorafenib combined with modified FOLFOX in patients with advanced hepatocellular carcinoma	2019	55
39	Ikeda et al.	A Phase 1b study of transforming growth factor-beta receptor I inhibitor galunisertib in combination with sorafenib in Japanese patients with unresectable hepatocellular carcinoma	2019	56
40	Choi et al.	Randomized, prospective, comparative study on the effects and safety of sorafenib vs. hepatic arterial infusion chemotherapy in patients with advanced hepatocellular carcinoma with portal vein tumor thrombosis	2018	57
41	Lim et al.	Phase II studies with refametinib or refametinib plus sorafenib in patients with RAS-mutated hepatocellular carcinoma	2018	58
42	Leal et al.	Survival and tolerance to sorafenib in Child-Pugh B patients with hepatocellular carcinoma: a prospective study	2018	59
43	Sato et al.	Multicenter Phase II clinical trial of sorafenib combined with transarterial chemoembolization for advanced stage hepatocellular carcinomas (Barcelona Clinic Liver Cancer Stage C): STAB study	2018	60
44	Thomas et al.	A randomized Phase II open-label multiinstitution study of the combination of bevacizumab and erlotinib compared to sorafenib in the first-line treatment of patients with advanced hepatocellular carcinoma	2018	61
45	Kudo et al.	Sorafenib plus low-dose cisplatin and fluorouracil hepatic arterial infusion chemotherapy versus sorafenib alone in patients with advanced hepatocellular carcinoma (SILIUS): a randomized, open label, Phase 3 trial	2018	62
46	Palmer et al.	A multicenter, open-label, phase-I/randomized Phase II study to evaluate safety, pharmacokinetics, and efficacy of nintedanib vs. sorafenib in European patients with advanced hepatocellular carcinoma	2018	63
47	Xu et al.	Sunitinib versus sorafenib plus transarterial chemoembolization for inoperable hepatocellular carcinoma patients	2018	64
48	El-Khoueiry et al.	A Phase I trial of escalating doses of cixutumumab (IMC-A12) and sorafenib in the treatment of advanced hepatocellular carcinoma	2018	65
49	Chow et al.	SIRveNIB: selective internal radiation therapy versus sorafenib in Asia-Pacific patients with hepatocellular carcinoma	2018	66
50	Suzuki et al.	A multicenter Phase II study of sorafenib in Japanese patients with advanced hepatocellular carcinoma and Child Pugh A and B class	2018	67
51	Kudo et al.	Lenvatinib versus sorafenib in first-line treatment of patients with unresectable hepatocellular carcinoma: a randomised Phase 3 noninferiority trial	2018	68
52	Zhang et al.	Combined endovascular brachytherapy, sorafenib, and transarterial chemobolization therapy for hepatocellular carcinoma patients with portal vein tumor thrombus	2017	69
53	Vilgrain et al.	Efficacy and safety of selective internal radiotherapy with yttrium-90 resin microspheres compared with sorafenib in locally advanced and inoperable hepatocellular carcinoma (SARAH): an open-label randomized controlled Phase 3 trial	2017	70
54	Meyer et al.	Sorafenib in combination with transarterial chemoembolization in patients with unresectable hepatocellular carcinoma (TACE 2): a randomised placebo-controlled, double-blind, Phase 3 trial	2017	71
55	Abou-Alfa et al.	Phase II study of first-line trebananib plus sorafenib in patients with advanced hepatocellular carcinoma	2017	72
56	Sho et al.	A phase I study of combination therapy with sorafenib and 5-fluorouracil in patients with advanced hepatocellular carcinoma	2017	73
57	Duffy et al.	Phase I and preliminary Phase II study of TRC105 in combination with sorafenib in hepatocellular carcinoma	2017	74
58	Ishizaki et al.	Phase I study of sorafenib in combination with intermittent hepatic arterial infusion chemotherapy for unresectable hepatocellular carcinoma	2017	75
59	Abou-Alfa et al.	Phase Ib study of codrituzumab in combination with sorafenib in patients with noncurable advanced hepatocellular carcinoma (HCC)	2017	76
60	Hubbard et al.	Phase I/II randomized trial of sorafenib and bevacizumab as first-line therapy in patients with locally advanced or metastatic hepatocellular carcinoma: North Central Cancer Treatment Group Trial N0745 (Alliance)	2017	77
61	Lin et al.	HATT: a Phase IV, single-arm, open-label study of sorafenib in Taiwanese patients with advanced hepatocellular carcinoma	2017	78
62	Giorgio et al.	Sorafenib combined with radio-frequency ablation compared with sorafenib alone in treatment of hepatocellular carcinoma invading portal vein: a Western randomized controlled trial	2016	79
63	Ikeda et al.	Sorafenib plus hepatic arterial infusion chemotherapy with cisplatin versus sorafenib for advanced hepatocellular carcinoma: randomized Phase II trial	2016	80
64	Merchante et al.	Real-life experience with sorafenib for the treatment of hepatocellular carcinoma in HIV-infected patients	2017	81
65	Tai et al.	A Phase Ib study of selumetinib (AZD6244, ARRY-142886) in combination with sorafenib in advanced hepatocellular carcinoma (HCC)	2016	82
66	Shahda et al.	Phase I study of lenalidomide and sorafenib in patients with advanced hepatocellular carcinoma	2016	83
67	Petrini et al.	Phase II trial of sorafenib in combination with 5-fluorouracil infusion in advanced hepatocellular carcinoma	2012	84
68	Song et al.	A single center experience of sorafenib in advanced hepatocellular carcinoma patients: evaluation of prognostic factors	2011	85
69	Gomez-Martin et al.	Efficacy and safety of sorafenib in combination with mammalian target of rapamycin inhibitors for recurrent hepatocellular carcinoma after liver transplantation	2012	86
70	Pawlik et al.	Phase II trial of sorafenib combined with concurrent transarterial chemoembolization with drug-eluting beads for hepatocellular carcinoma	2011	87
71	Lee et al.	Phase 1 trial of S-1 in combination with sorafenib for patients with advanced hepatocellular carcinoma	2012	88
72	Kudo et al.	Phase III study of sorafenib after transarterial chemoembolization in Japanese and Korean patients with unresectable hepatocellular carcinoma	2011	89
73	Coriat et al.	Reversible decrease of portal venous flow in cirrhotic patients: a positive side effect of sorafenib	2011	90
74	Abou-Alfa et al.	Doxorubicin plus sorafenib vs doxorubicin alone in patients with advanced hepatocellular carcinoma: a randomized trial	2010	91
75	Dufouret al.	Continuous administration of sorafenib in combination with transarterial chemoembolization in patients with hepatocellular carcinoma: results of a Phase I study	2010	92
76	Hsu et al.	Phase II study of combining sorafenib with metronomic tegafur/uracil for advanced hepatocellular carcinoma	2010	93
77	Prete et al.	Sorafenib plus octreotide is an effective and safe treatment in advanced hepatocellular carcinoma: multicenter Phase II So.LAR. study	2010	94
78	Jeong et al.	Practical effect of sorafenib monotherapy on advanced hepatocellular carcinoma and portal vein tumor thrombosis	2013	95
79	Cheng et al.	Sunitinib versus sorafenib in advanced hepatocellular cancer: results of a randomized Phase III trial	2013	96
80	Johnson et al.	Brivanib versus sorafenib as first-line therapy in patients with unresectable, advanced hepatocellular carcinoma: results from the randomized Phase III BRISK-FL study	2013	97
81	Finn et al.	Phase I study investigating everolimus combined with sorafenib in patients with advanced hepatocellular carcinoma	2013	98
82	Abdel-Rahman et al.	Sorafenib versus capecitabine in the management of advanced hepatocellular carcinoma	2013	99
83	Jia et al.	Phase I adjuvant trial of sorafenib in patients with hepatocellular carcinoma after orthotopic liver transplantation	2013	100
84	Kelley et al.	Temsirolimus combined with sorafenib in hepatocellular carcinoma: a Phase I dose-finding trial with pharmacokinetic and biomarker correlates	2013	101
85	Kostner et al.	Sorafenib in advanced hepatocellular carcinoma: a nationwide retrospective study of efficacy and tolerability	2013	102
86	Bai et al.	Sorafenib in combination with transarterial chemoembolization improves the survival of patients with unresectable hepatocellular carcinoma: a propensity score matching study	2013	103
87	Brunocilla et al.	Sorafenib in hepatocellular carcinoma: prospective study on adverse events, quality of life, and related feasibility under daily conditions	2013	104
88	Pressiani et al.	Sorafenib in patients with Child-Pugh classes A and B advanced hepatocellular carcinoma: a prospective feasibility analysis	2013	105
89	Jeong et al.	The efficacy of hepatic arterial infusion chemotherapy as an alternative to sorafenib in advanced hepatocellular carcinoma	2012	106
90	Sansonno et al.	Transarterial chemoembolization plus sorafenib: a sequential therapeutic scheme for HCV-related intermediate-stage hepatocellular carcinoma: a randomized clinical trial	2012	107
91	Park et al.	Phase II study of concurrent transarterial chemoembolization and sorafenib in patients with unresectable hepatocellular carcinoma	2012	108
92	Sieghart et al.	Conventional transarterial chemoembolization in combination with sorafenib for patients with hepatocellular carcinoma: a pilot study	2012	109
93	Bitzer et al.	Resminostat plus sorafenib as second-line therapy of advanced hepatocellular carcinoma—the SHELTER study	2016	110
94	Koeberle et al.	Sorafenib with or without everolimus in patients with advanced hepatocellular carcinoma (HCC): a randomized multicenter, multinational Phase II trial (SAKK 77/08 and SASL 29)	2016	111
95	Brade et al.	Phase 1 trial of sorafenib and stereotactic body radiation therapy for hepatocellular carcinoma	2016	112
96	Lencioni et al.	Sorafenib or placebo plus TACE with doxorubicin-eluting beads for intermediate stage HCC: the SPACE trial	2016	113
97	Ciuleanu et al.	A randomized, double-blind, placebo-controlled Phase II study to assess the efficacy and safety of mapatumumab with sorafenib in patients with advanced hepatocellular carcinoma	2016	114
98	Zhang et al.	Sorafenib with and without transarterial chemoembolization for advanced hepatocellular carcinoma with main portal vein tumor thrombosis: a retrospective analysis	2015	115
99	Bruix et al.	Adjuvant sorafenib for hepatocellular carcinoma after resection or ablation (STORM): a Phase 3, randomised, double-blind, placebo-controlled trial	2015	116
100	Cheng et al.	Safety and efficacy of tigatuzumab plus sorafenib as first-line therapy in subjects with advanced hepatocellular carcinoma: a Phase 2 randomized study	2015	117
101	Hoffmann et al.	Impact of neo-adjuvant Sorafenib treatment on liver transplantation in HCC patients—a prospective, randomized, double-blind, Phase III trial	2015	118
102	Cosgrove et al.	Open-label single-arm Phase II trial of sorafenib therapy with drug-eluting bead transarterial chemoembolization in patients with unresectable hepatocellular carcinoma: clinical results	2015	119
103	Yao et al.	Concurrent sorafenib therapy extends the interval to subsequent TACE for patients with unresectable hepatocellular carcinoma	2016	120
104	Cheng et al.	Randomized, open-label Phase 2 study comparing frontline dovitinib versus sorafenib in patients with advanced hepatocellular carcinoma	2016	121
105	Adjei et al.	A Phase I study of the safety, pharmacokinetics, and pharmacodynamics of combination therapy with refametinib plus sorafenib in patients with advanced cancer	2016	122
106	Kan et al.	Sorafenib combined with percutaneous radiofrequency ablation for the treatment of medium-sized hepatocellular carcinoma	2015	123
107	Turnes et al.	[Therapeutic decisions in the treatment of hepatocellular carcinoma and patterns of sorafenib use. Results of the international observational GIDEON trial in Spain]	2015	124
108	Zhu et al.	SEARCH: a Phase III, randomized, double-blind, placebo-controlled trial of sorafenib plus erlotinib in patients with advanced hepatocellular carcinoma	2015	125
109	Cainap et al.	Linifanib versus sorafenib in patients with advanced hepatocellular carcinoma: results of a randomized Phase III trial	2015	126
110	Lim et al.	A Phase II study of the efficacy and safety of the combination therapy of the MEK inhibitor refametinib (BAY 86-9766) plus sorafenib for Asian patients with unresectable hepatocellular carcinoma	2014	127
111	Puzanov et al.	Phase 1 trial of tivantinib in combination with sorafenib in adult patients with advanced solid tumors	2015	128
112	Erhardt et al.	TACE plus sorafenib for the treatment of hepatocellular carcinoma: results of the multicenter, phase II SOCRATES trial	2014	129
113	Chao et al.	The combination of transcatheter arterial chemoembolization and sorafenib is well tolerated and effective in Asian patients with hepatocellular carcinoma: final results of the START trial	2015	130
114	Lee et al.	Randomized Phase II study of the X-linked inhibitor of apoptosis (XIAP) antisense AEG35156 in combination with sorafenib in patients with advanced hepatocellular carcinoma (HCC)	2016	131
115	Ricke et al.	Safety and toxicity of radioembolization plus sorafenib in advanced hepatocellular carcinoma: analysis of the European multicenter trial SORAMIC	2015	132
116	Zheng et al.	Analysis of survival factors in patients with intermediate-advanced hepatocellular carcinoma treated with transcatheter arterial chemoembolization combined with sorafenib	2014	133
117	Srimuninnimit et al.	Efficacy and safety of sorafenib in combination with gemcitabine in patients with advanced hepatocellular carcinoma: a multicenter, open-label, single-arm Phase II study	2014	134
118	Wei et al.	Neutrophil–lymphocyte ratio as a predictor of outcomes for patients with hepatocellular carcinoma undergoing TAE combined with sorafenib	2014	135
119	Imedio et al.	Safety and efficacy of sorafenib in the treatment of advanced hepatocellular carcinoma: a single center experience	2014	136
120	Ji et al.	Sorafenib in liver function impaired advanced hepatocellular carcinoma	2014	137
121	Kulik et al.	Prospective randomized pilot study of Y90+/–sorafenib as bridge to transplantation in hepatocellular carcinoma	2014	138
122	Chen et al.	Phase 2 study of combined sorafenib and radiation therapy in patients with advanced hepatocellular carcinoma	2014	139
123	Chow et al.	Multicenter phase II study of sequential radioembolization-sorafenib therapy for inoperable hepatocellular carcinoma	2014	140
124	Ooka et al.	A Phase I/II study of S-1 with sorafenib in patients with advanced hepatocellular carcinoma	2014	141
125	Hagihara et al.	Phase I study of combination chemotherapy using sorafenib and transcatheter arterial infusion with cisplatin for advanced hepatocellular carcinoma	2014	142
126	Cho et al.	The feasibility of combined transcatheter arterial chemoembolization and radiotherapy for advanced hepatocellular carcinoma	2014	143
127	Nagi et al.	Efficacy and safety of sorafenib–gemcitabine combination therapy in advanced hepatocellular carcinoma: an open-label Phase II feasibility study	2014	144
128	Castello et al.	Metabolic switch in hepatocellular carcinoma patients treated with sorafenib: a proof-of-concept trial	2020	145
129	Kim et al.	Efficacy and safety of liver-directed concurrent chemoradiotherapy and sequential sorafenib for advanced hepatocellular carcinoma: a prospective Phase 2 trial	2020	146
130	Kudo et al.	Randomized, multicenter prospective trial of transarterial chemoembolization (TACE) plus sorafenib as compared with TACE alone in patients with hepatocellular carcinoma: TACTICS trial	2020	147
131	Kondo et al.	Randomized, Phase II trial of sequential hepatic arterial infusion chemotherapy and sorafenib versus sorafenib alone as initial therapy for advanced hepatocellular carcinoma: SCOOP-2 trial	2019	148
132	Mokdad et al.	Efficacy and safety of bavituximab in combination with sorafenib in advanced hepatocellular carcinoma: a single-arm, open-label, Phase II clinical trial	2019	149
133	Gordon et al.	Phase I study of sorafenib and vorinostat in advanced hepatocellular carcinoma	2019	150
134	Kelley et al.	A Phase 2 study of galunisertib (TGF-β1 receptor type I inhibitor) and sorafenib in patients with advanced hepatocellular carcinoma	2019	151
135	Eilard et al.	A prospective clinical trial on sorafenib treatment of hepatocellular carcinoma before liver transplantation	2019	152
136	Jouve et al.	Pravastatin combination with sorafenib does not improve survival in advanced hepatocellular carcinoma	2019	153

### Characteristics of included studies

**Figure 2.**
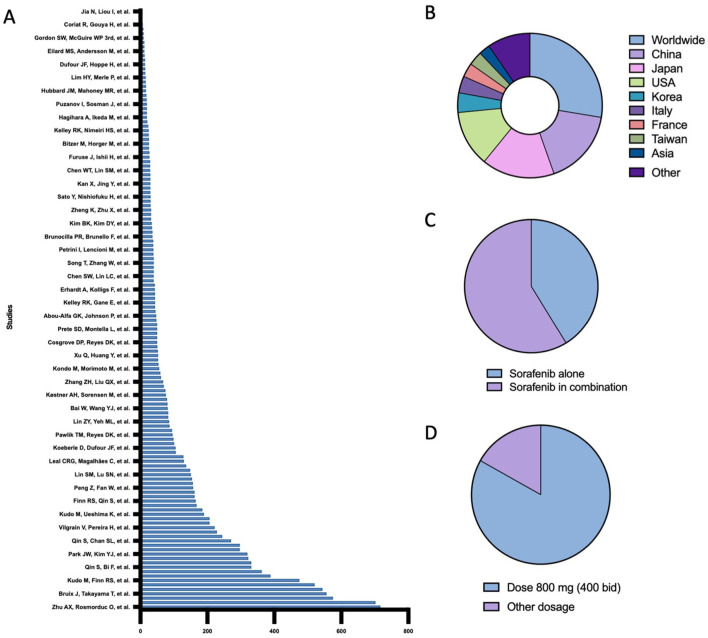
Overview of included studies and treatment modalities. (a) Distribution of sample sizes among studies investigating Sorafenib treatment. The bar chart presents the sample size for each study. (b) Geographic distribution of the studies, shown as a donut chart. The chart depicts the proportion of studies conducted in different countries, with multinational, China, and Japan representing the largest contributions. (c) Proportion of studies using Sorafenib alone versus in combination with another treatment modality. (d) Proportion of studies investigating Sorafenib at a starting dose of 800 mg versus another dosage.

The included clinical studies showed heterogeneity in design, geographical location, and sample size. Most (62 studies) were RCTs, including Phase III trials, followed by 39 Phase II trials and 3 prospective cohort studies. Studies including multiple treatments were divided per treatment arm to allow assessment of differences in sorafenib treatment settings. Altogether, the included studies covered 14,416 patients, with individual study sizes ranging from 4 participants^100^ to 717 participants^124^ (Figure 2(a)). The geographical distribution spanned 20 countries, with the majority of studies conducted internationally across multiple regions, followed by single-country studies predominantly from China and Japan (Figure 2(b)).

This systematic review included studies evaluating diverse sorafenib treatment modalities. Particular focus was given to studies comparing different dosing regimens (Figure 2(c)) or assessing sorafenib as monotherapy versus combination therapy with systemic or locoregional interventions (Figure 2(d)). Each treatment arm including sorafenib was analyzed independently, yielding a total of 178 study arms. Among these, 83% reported standard-dose sorafenib (800 mg daily), while 17% investigated alternative dosing strategies.^28,30,40,45,53,110,128,132,140^ Sorafenib was administered in combination with other treatments in 41% of study arms and as monotherapy in 69%. The most common combination regimen involved TACE.^7,18,24,28,39,53,64,69,71,87,89,92,103,107–109,113,115,118–120,129,130,132,133,143,147,152^

### Prevalence and reporting of gastrointestinal side-effects

**Figure 3.**
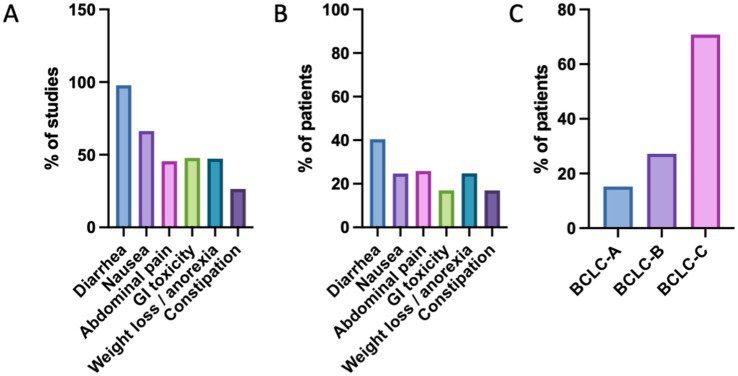
Documentation and prevalence of GI side effects and BCLC staging. (a) Percentage of included studies reporting each GI-related adverse effect. (b) Percentage of total patients reporting each GI-related adverse effect. (c) Percentage of total patients by BCLC stage. BCLC, Barcelona-Clinic Liver Cancer; GI, gastrointestinal.

In our meta-analysis of 136 studies (178 study arms; 14,416 patients), we found variability in GI side effect reporting and documentation (Figure 3(a) and Table 1). Diarrhea was the most consistently reported symptom, documented in 132 studies (174 study arms; 6053 cases), corresponding to 97.8% of all included studies. The mean prevalence of diarrhea was 40.44% (SEM 1.59; 95% CI: 37.31–43.58), with a weighted prevalence of 42.21%. Nausea was reported in 92 studies (118 study arms; 2298 cases), representing 66.3% of studies, with a mean prevalence of 24.75% (SEM 1.79; 95% CI: 21.19–28.28) and a weighted prevalence of 20.35%. Abdominal pain was reported in 62 studies (81 study arms; 2105 cases), accounting for 45.5% of studies, with a mean prevalence of 25.85% (SEM 2.18; 95% CI: 21.52–30.18) and a weighted prevalence of 22.30%. GI toxicity, including ulcerations, intestinal hemorrhages, and mucositis, was documented in 69 studies (85 study arms; 867 cases), corresponding to 47.8% of studies, with a mean prevalence of 16.96% (SEM 1.95; 95% CI: 13.07–20.85) and a weighted prevalence of 16.11%. Weight loss or anorexia was reported in 66 studies (84 study arms; 2047 cases), representing 47.2% of studies, with a mean prevalence of 24.79% (SEM 2.12; 95% CI: 20.58–29.00) and a weighted prevalence of 22.67%. Finally, constipation was reported in 33 studies (47 study arms; 813 cases), corresponding to 26.4% of studies, with a mean prevalence of 16.93% (SEM 1.47; 95% CI: 13.97–19.89) and a weighted prevalence of 14.13%. When stratified by Barcelona-Clinic Liver Cancer (BCLC) stage, the majority of patients across the included trials were in stage C, consistent with the clinical use of sorafenib in intermediate to advanced HCC.^1^

### Diarrhea

**Figure 4.**
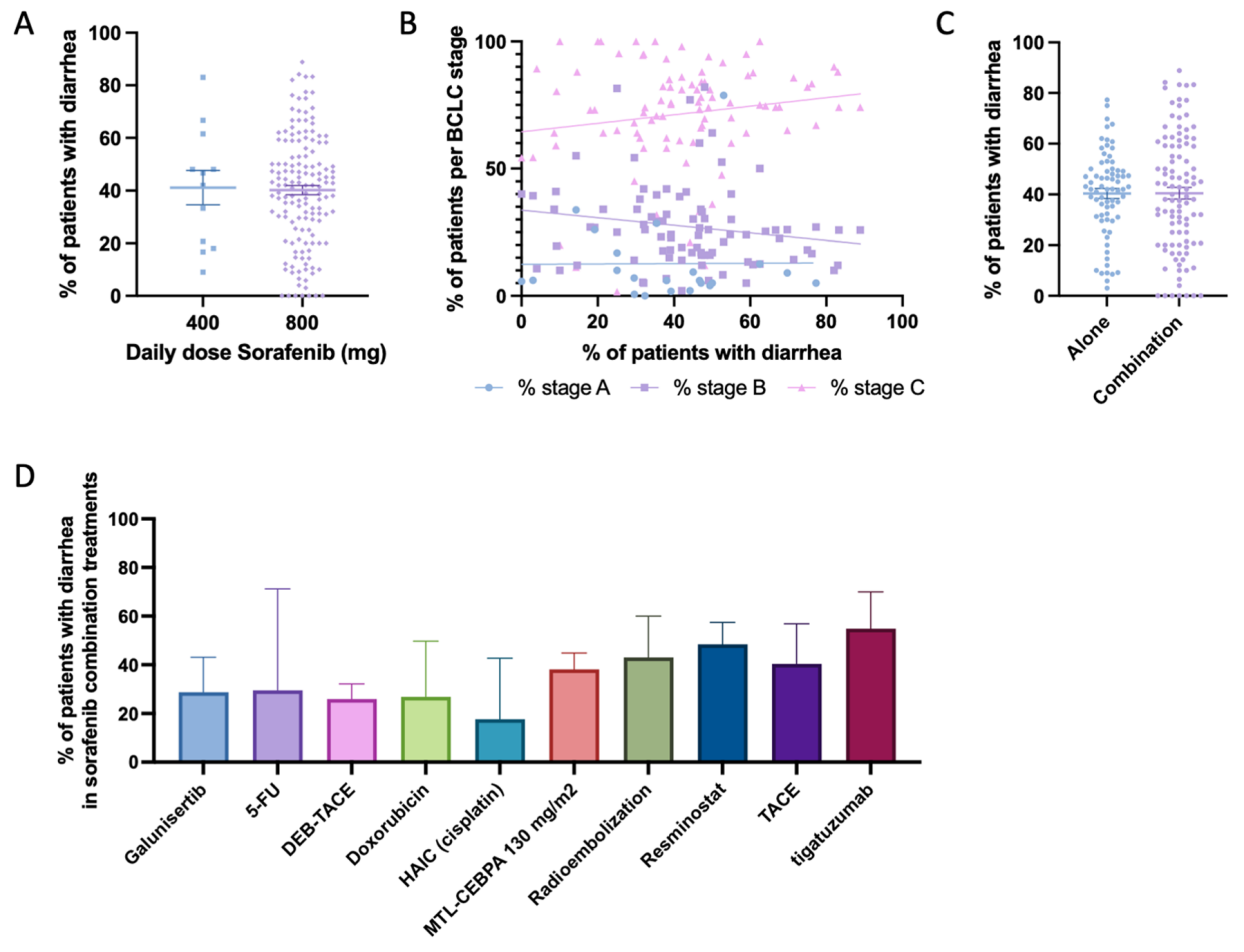
Prevalence of diarrhea in patients undergoing Sorafenib treatment. (a) Comparison of percentage of patients experiencing diarrhea between those treated with 400 mg daily dose Sorafenib versus 800 mg daily dose. (b) Prevalence of patients experiencing diarrhea, cross-examined with prevalence of patients within each Barcelona-Clinic Liver Cancer classification. (c) Comparison of percentage of patients experiencing diarrhea between those treated with Sorafenib alone versus Sorafenib combined with another treatment modality. (d) Bar chart depicting the prevalence of patients experiencing diarrhea while undergoing Sorafenib and a combinational treatment, categorized by combinational treatment modality.

Our meta-analysis identified diarrhea as the most frequently reported gastrointestinal side effect of sorafenib, documented in 132 studies (174 study arms; 6053 cases; Table 2). The mean prevalence across these studies was 40.44% (SEM 1.59; 95% CI: 37.31–43.58), with a weighted prevalence of 42.21% (Figure 3(b)). When stratified by daily dose, patients receiving standard-dose sorafenib (800 mg/day) experienced diarrhea at similar rates compared with those receiving reduced doses (400 mg/day), although the scatter plots suggest considerable inter-study variability (Figure 4(a) and (b)). No clear association was observed between diarrhea prevalence and stage distribution; trials with higher or lower rates of diarrhea included similar proportions of BCLC B and C patients. Stage A patients were rarely represented, and when present, diarrhea rates did not differ markedly from trials dominated by stage B or C cohorts. In treatment setting comparisons, sorafenib monotherapy and combination therapies both showed substantial rates of diarrhea, with no differences in combination regimens (Figure 4(c)). Among combination strategies, the highest proportions of diarrhea were observed when sorafenib was combined with resminostat, TACE, and tigatuzumab^54,110,117^ (Figure 4(d)).

**Table 2.** Prevalence of gastrointestinal symptoms in HCC-patients receiving Sorafenib.

Symptom	Number of studies (*n*)	Number of study arms (*n*)	Cases (*n*)	Mean prevalence (%, SEM, CI)	Weighted prevalence (%)
Diarrhea	132	174	6053	40.44% (1.59, CI: 37.31–43.58)	42.21
Nausea	92	118	2298	24.75% (1.79, CI: 21.19–28.28)	20.35
Abdominal pain	62	81	2105	25.85% (2.176, CI: 21.52–30.18)	22.30
GI toxicity	69	85	867	16.96% (1.95, CI: 13.07–20.85)	16.11
Weight loss or anorexia	66	84	2047	24.79% (2.12, CI: 20.58–29.00)	22.67
Constipation	33	47	813	16.93% (1.47, CI: 13.97–19.89)	14.13

GI, gastrointestinal; SEM, standard error of the mean.

### Nausea

**Figure 5.**
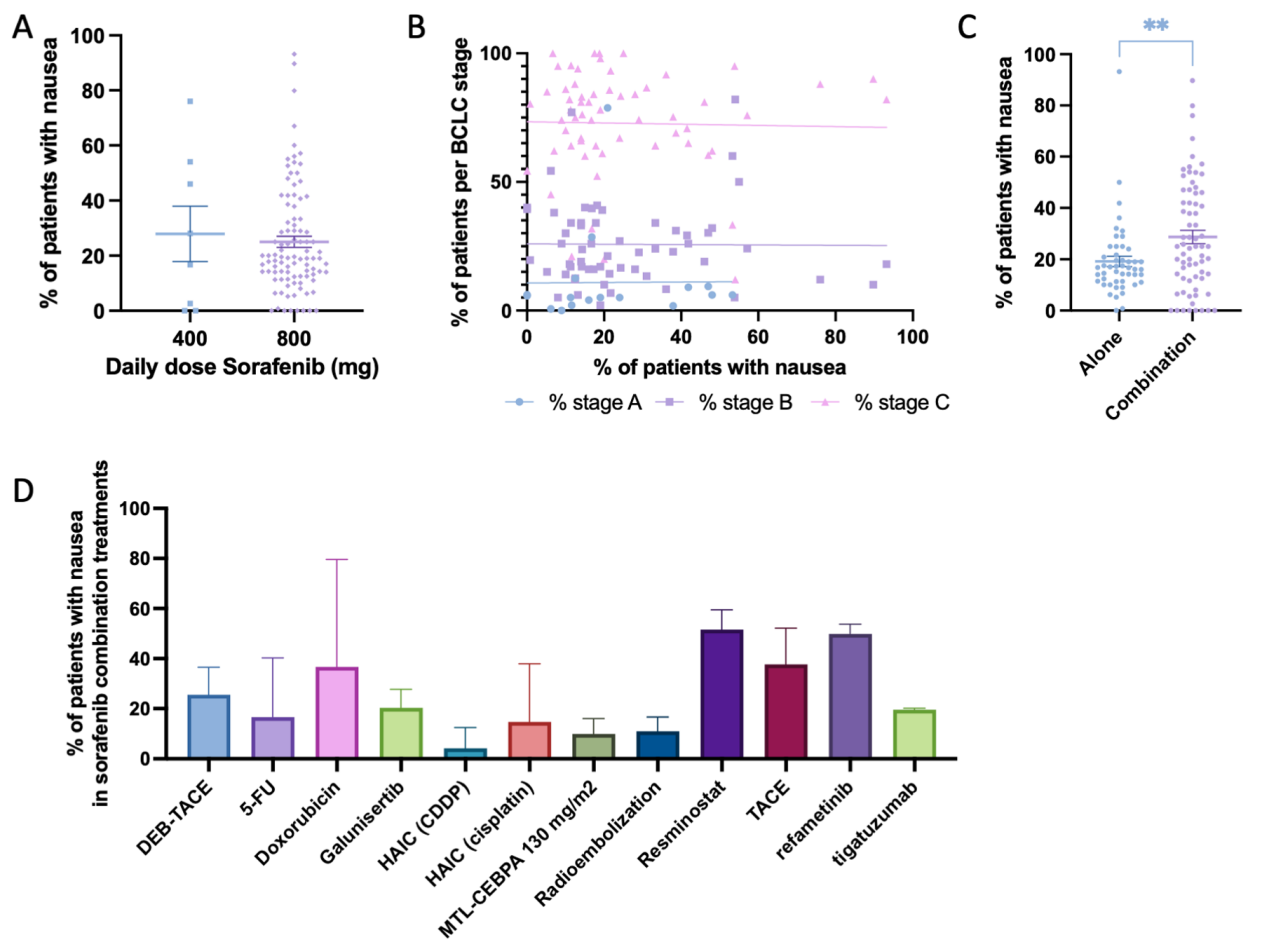
Prevalence of nausea in patients undergoing Sorafenib treatment. (a) Comparison of percentage of patients experiencing nausea between those treated with 400 mg daily dose Sorafenib versus 800 mg daily dose. (b) Prevalence of patients experiencing nausea, cross-examined with prevalence of patients within each Barcelona-Clinic Liver Cancer classification. (c) Comparison of percentage of patients experiencing nausea between those treated with Sorafenib alone versus Sorafenib combined with another treatment modality. (d) Bar chart depicting the prevalence of patients experiencing nausea while undergoing Sorafenib and a combinational treatment, categorized by combinational treatment modality. Significant differences are indicated by asterisks: ** = *p* < 0.01.

Nausea was the second most frequently reported gastrointestinal side effect in sorafenib-treated patients, documented in 92 studies (118 study arms; 2298 cases; Table 2). The mean prevalence across studies was 24.75% (SEM 1.79; 95% CI: 21.19–28.28), with a weighted prevalence of 20.35% (Figure 3(b)). When stratified by daily dose, patients receiving standard-dose sorafenib (800 mg/day) showed nausea rates comparable to those receiving reduced doses (400 mg/day), although inter-study variability was evident (Figure 5(a)). Scatter plots stratified by BCLC stage showed that most trials primarily included stage B and C patients, as expected for sorafenib therapy. No clear correlation was observed between the proportion of patients at a given stage and the prevalence of nausea in each trial (Figure 5(b)). When comparing treatment settings, nausea was significantly more common in combination regimens than in sorafenib monotherapy (Figure 5(c)). Among the combination strategies, higher rates of nausea were observed when sorafenib was combined with TACE, resminostat, or refametinib, with mean prevalence exceeding 40% in these studies (Figure 5(d)).^54,58,110,122,127^ In contrast, combinations with HAIC-based regimens or radioembolization reported lower prevalence rates, generally below 20%.

### GI toxicity

**Figure 6.**
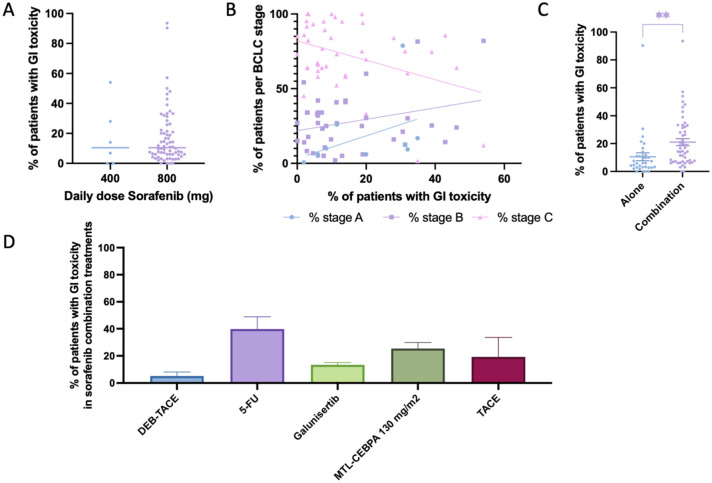
Prevalence of GI toxicity in patients undergoing Sorafenib treatment. (a) Comparison of percentage of patients experiencing GI toxicity between those treated with 400 mg daily dose Sorafenib versus 800 mg daily dose. (b) Prevalence of patients experiencing GI toxicity, cross-examined with prevalence of patients within each Barcelona-Clinic Liver Cancer classification. (c) Comparison of percentage of patients experiencing GI toxicity between those treated with Sorafenib alone versus Sorafenib combined with another treatment modality. (d) Bar chart depicting the prevalence of patients experiencing GI toxicity while undergoing Sorafenib and a combinational treatment, categorized by combinational treatment modality. GI, gastrointestinal. Significant differences are indicated by asterisks: ** = *p* < 0.01.

GI toxicity, defined as gastrointestinal ulcerations, hemorrhages, or mucositis, was reported in 69 studies (85 study arms; 867 cases; Table 2). The mean prevalence across these studies was 16.96% (SEM 1.95; 95% CI: 13.07–20.85), with a weighted prevalence of 16.11% (Figure 3(b)). When stratified by daily dose, there was no clear difference in the prevalence of GI toxicity between patients receiving standard-dose sorafenib (800 mg/day) and those on reduced doses (400 mg/day), although the overall prevalence remained relatively low compared with other GI AEs (Figure 6(a)). Scatter plots stratified by BCLC stage indicated that most trials included predominantly stage B and C patients, with no obvious correlation between the proportion of patients at each stage and reported prevalence of GI toxicity (Figure 6(b)). Comparisons between treatment settings revealed that GI toxicity was significantly more common in combination regimens compared to sorafenib monotherapy (Figure 6(c)). Among the combination strategies, the highest rates of GI toxicity were observed with sorafenib combined with 5-FU,^55,73,106^ where prevalence exceeded 40% in some studies (Figure 6(d)). Other combinations, including TACE, MTL-CEBPA,^37^ and galunisertib,^56,151^ showed intermediate prevalence rates while DEB-TACE combinations were associated with the lowest rates.

### Abdominal pain

**Figure 7.**
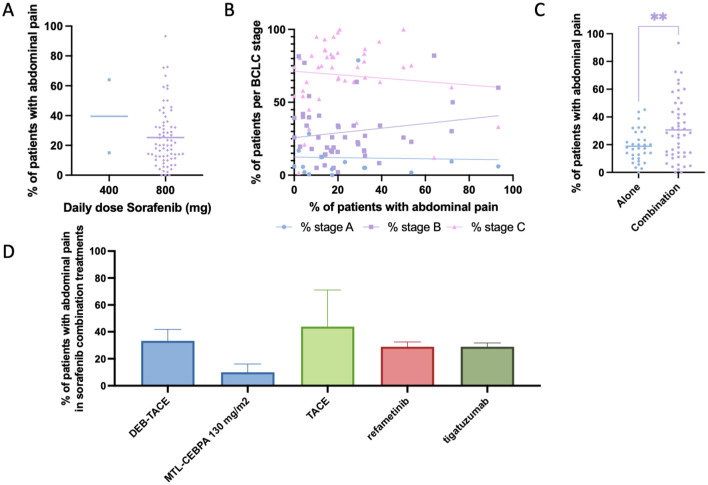
Prevalence of abdominal pain in patients undergoing Sorafenib treatment. (a) Comparison of percentage of patients experiencing abdominal pain between those treated with 400 mg daily dose Sorafenib versus 800 mg daily dose. (b) Prevalence of patients experiencing abdominal pain, cross-examined with prevalence of patients within each Barcelona-Clinic Liver Cancer classification. (c) Comparison of percentage of patients experiencing abdominal pain between those treated with Sorafenib alone versus Sorafenib combined with another treatment modality. (d) Bar chart depicting the prevalence of patients experiencing abdominal pain while undergoing Sorafenib and a combinational treatment, categorized by combinational treatment modality. Significant differences are indicated by asterisks: ** = *p* < 0.01.

Abdominal pain was reported in 62 studies (81 study arms; 2105 cases; Table 2). The mean prevalence across these studies was 25.85% (SEM 2.18; 95% CI: 21.52–30.18), with a weighted prevalence of 22.30% (Figure 3(b)). When stratified by daily dose, abdominal pain was observed at comparable frequencies in patients receiving standard-dose sorafenib (800 mg/day) and those on reduced doses (400 mg/day), with wide variability between trials (Figure 7(a)). Scatter plots stratified by BCLC stage showed that most trials enrolled predominantly stage B and C patients, with no apparent correlation between stage distribution and abdominal pain prevalence (Figure 7(b)). Comparisons between treatment settings demonstrated that abdominal pain was significantly more common in combination regimens than in sorafenib monotherapy (Figure 7(c)). Among combination strategies, the highest rates were observed in studies combining sorafenib with TACE, where mean prevalence exceeded 40% (Figure 7(d)). Elevated prevalence was also reported in combinations with refametinib and tigatuzumab,^58,117,122,127^ while combinations with DEB-TACE^87,113^ or MTL-CEBPA^37^ were associated with lower prevalence.

### Weight loss or anorexia

**Figure 8.**
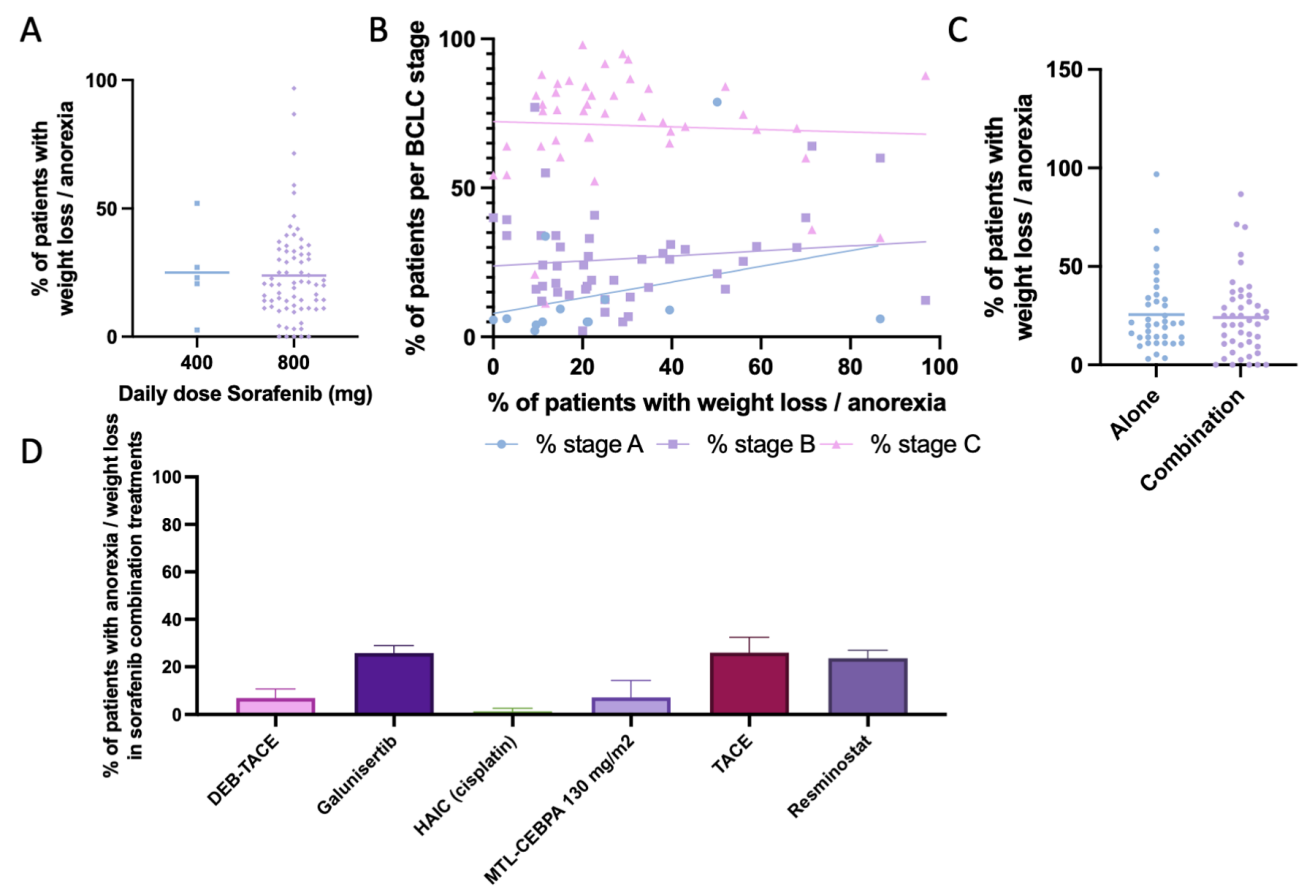
Prevalence of weight loss/anorexia in patients undergoing Sorafenib treatment. (a) Comparison of percentage of patients experiencing weight loss/anorexia between those treated with 400 mg daily dose Sorafenib versus 800 mg daily dose. (b) Prevalence of patients experiencing weight loss/anorexia, cross examined with prevalence of patients within each Barcelona-Clinic Liver Cancer classification. (c) Comparison of percentage of patients experiencing weight loss/anorexia between those treated with Sorafenib alone versus Sorafenib combined with another treatment modality. (d) Bar chart depicting the prevalence of patients experiencing weight loss/anorexia while undergoing Sorafenib and a combinational treatment, categorized by combinational treatment modality.

Weight loss or anorexia was reported in 66 studies (84 study arms; 2047 cases; Table 2). The mean prevalence across studies was 24.79% (SEM 2.12; 95% CI: 20.58–29.00), with a weighted prevalence of 22.67% (Figure 3(b)). When stratified by daily dose, prevalence of weight loss and anorexia was similar in patients receiving standard-dose sorafenib (800 mg/day) and those on reduced doses (400 mg/day), though variability between studies was considerable (Figure 8(a)). Scatter plots stratified by BCLC stage showed that most trials enrolled predominantly stage B and C patients, with no apparent correlation between stage distribution and abdominal pain prevalence (Figure 8(b)). Unlike several other gastrointestinal side effects, no significant difference was observed between sorafenib monotherapy and combination regimens (Figure 8(c)). Within combination arms, prevalence varied according to the partner treatment: higher rates were observed with galunisertib, TACE, and resminostat, while lower rates were reported in combinations with DEB-TACE, HAIC, or MTL-CEBPA (Figure 8(d)).

### Constipation

**Figure 9.**
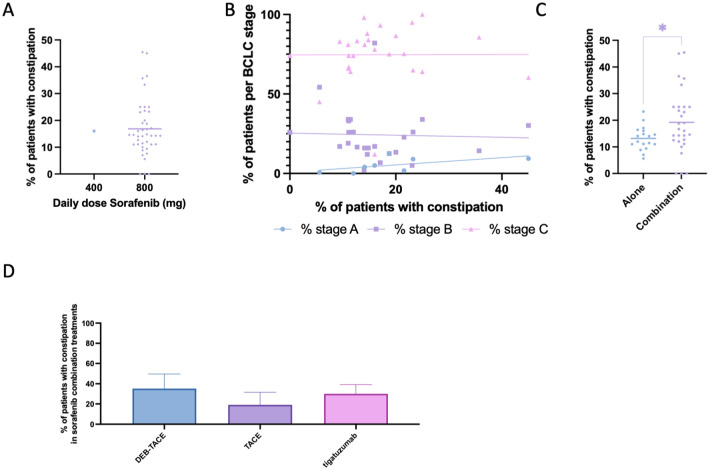
Prevalence of constipation in patients undergoing sorafenib treatment. (a) Comparison of percentage of patients experiencing constipation between those treated with 400 mg daily dose sorafenib versus 800 mg daily dose. (b) Prevalence of patients experiencing constipation, cross-examined with prevalence of patients within each Barcelona-Clinic Liver Cancer classification. (c) Comparison of percentage of patients experiencing constipation between those treated with sorafenib alone versus sorafenib combined with another treatment modality. (d) Bar chart depicting the prevalence of patients experiencing constipation while undergoing sorafenib and a combinational treatment, categorized by combinational treatment modality. Significant differences are indicated by asterisks: * = *p* < 0.05.

Constipation was the least frequently reported gastrointestinal side effect in sorafenib-treated patients, documented in 33 studies (47 study arms; 813 cases; Table 2). The mean prevalence across studies was 16.93% (SEM 1.47; 95% CI: 13.97–19.89), with a weighted prevalence of 14.13% (Figure 3(b)). When stratified by daily dose, interpretation was limited because only one study using a reduced dose of sorafenib (400 mg/day) reported constipation. As such, no reliable conclusions can be drawn about dose–response patterns, and the overall prevalence estimates are primarily driven by standard-dose sorafenib studies (Figure 9(a)). Scatter plots stratified by BCLC stage suggested a slight tendency toward higher prevalence in trials enrolling larger proportions of stage C patients, although no strong correlation was evident (Figure 9(b)). In contrast to diarrhea and weight loss, constipation was significantly more common in combination regimens compared with sorafenib monotherapy (Figure 9(c)). Among combination strategies, constipation was most frequently reported with tigatuzumab and DEB-TACE,^87,113,117^ while lower rates were observed in sorafenib combined with TACE (Figure 9(d)).

### Overall GI-adverse effects

**Figure 10.**
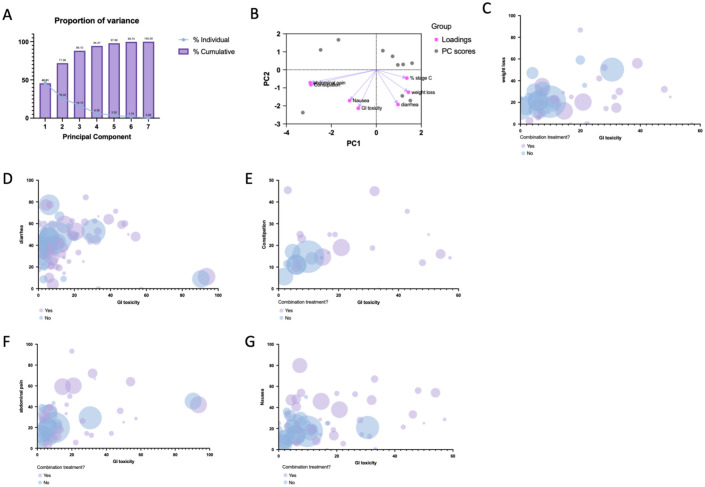
PCA and study-level prevalence of gastrointestinal toxicities in sorafenib based treatments. (a) Screen plot showing the proportion of variance explained by each principal component. PC1 and PC2 together capture ~70% of the variability across study arms. (b) PCA biplot of the first two components, with loadings (purple) and study arms (gray). GI toxicity, diarrhea, nausea, abdominal pain, and weight loss cluster along PC1, indicating they are the major drivers of variation. Constipation and proportion of stage C patients load separately along PC2. (c–g) Bubble plots showing the relationship between overall GI toxicity (*x*-axis) and other adverse events (*y*-axis), including weight loss (c), diarrhea (d), constipation (e), abdominal pain (f), and nausea (g). Each dot represents a study arm, with bubble size proportional to sample size and color indicating sorafenib monotherapy (blue) or combination therapy (purple). PC1, principal component 1; PC2, principal component 2; PCA, principal component analysis.

To further identify the relationships between gastrointestinal side effects, we performed PCA across all study arms. The first two components explained 71.96% of the variance (Figure 10(a)). The biplot revealed that diarrhea, weight loss/anorexia, and GI toxicity clustered together, indicating partial overlap in reporting and prevalence patterns across studies (Figure 10(b)). In contrast, nausea and abdominal pain loaded more independently, suggesting they may represent distinct AE profiles.

Dimensionality reduction via PCA was applied to identify clusters within the dataset based on treatment modalities (Figure 10(c)–(g)). When stratifying bubble plots by treatment setting, combination regimens (purple) were consistently associated with greater heterogeneity in GI toxicity prevalence compared with sorafenib monotherapy (blue; Figure 10(c)–(h)). Higher GI toxicity prevalence tended to coincide with increased rates of diarrhea and anorexia/weight loss (Figure 10(c) and (d)), aligning with the clustering patterns observed in the PCA. For nausea, sorafenib monotherapy studies clustered at low prevalence, while combination regimens were more dispersed, with some reporting concurrent elevations of nausea and GI toxicity (Figure 10(e)).

## Discussion

This meta-analysis of 136 clinical trials including 14,416 patients provides a comprehensive summary of GI AE associated with sorafenib treatment in HCC. Our results confirm that GI side effects are frequent, with diarrhea as the most consistently reported symptom, affecting over 40% of patients, similar to what has been noted in other studies.^4,5,12^ Nausea, abdominal pain, weight loss/anorexia, GI toxicity (including ulcerations and hemorrhage), and constipation were also observed at meaningful prevalence levels, though with considerable variability across studies. Importantly, adverse effect profiles differed between monotherapy and combination regimens, and multivariate analyses highlighted symptom clusters that may reflect shared pathophysiological mechanisms.

When compared with our previous meta-analysis of GI adverse effects in TACE,^11^ sorafenib demonstrates a different side effect profile. While abdominal pain was the most common complication of TACE (reflecting the embolization procedure itself), sorafenib treatment was dominated by systemic toxicities, with diarrhea as the leading symptom.^11^ Nausea and anorexia/weight loss were also more prominent in sorafenib, whereas GI ulcerations and hemorrhage, though less frequent, carry potential clinical significance due to their impact on patient safety and treatment discontinuation. These differences underscore the distinct mechanisms underlying locoregional and systemic treatments: ischemia-driven postembolization syndromes in TACE^11,154^ versus off-target kinase inhibition and epithelial stress in sorafenib.^11,13,16^

Our stratified analyses revealed that most gastrointestinal side effects occurred at similar rates in standard-dose (800 mg/day) and reduced-dose (400 mg/day) sorafenib, although inter-study variability was wide. This finding suggests that toxicity may not increase in a strictly dose-dependent manner, but instead be influenced by patient-specific susceptibility, duration of exposure, and the use of supportive care interventions. Notably, fewer studies were conducted with a reduced-dose, raising the possibility of reporting bias in more complex trial settings. However, real-world data support the clinical relevance of dose adjustments. A recent retrospective evaluated 80 patients treated with reduced-dose sorafenib (400 mg/day) and reported a clinical benefit rate of 56%, with median progression-free survival of 3.7 months and overall survival of 5.3 months.^155^ Grade 3 toxicities occurred in only 11% of patients, and just 7.5% discontinued treatment due to AEs despite the lower dose.^155^ Similarly, a large Veterans Health Administration cohort of 4903 patients demonstrated that reduced starting doses (<800 mg/day) were not associated with inferior overall survival after propensity matching, but were linked to lower discontinuation rates due to gastrointestinal toxicity and reduced cumulative treatment costs.^156^ Together, these findings support dose reduction as a clinically reasonable option in selected patients, particularly those with poorer baseline liver function, higher comorbidity burden, or limited access to alternative therapies.

Combination therapies consistently showed higher and more variable prevalence of several adverse effects compared with monotherapy. Diarrhea and nausea, for example, were particularly common in regimens combining sorafenib with TACE, resminostat, or refametinib. In contrast, combinations with DEB-TACE or HAIC were associated with lower rates, suggesting that treatment-specific interactions may shape the GI toxicity profile. These findings highlight the importance of carefully selecting drug combination and monitoring additive toxicities, particularly in patients with limited hepatic reserve.

PCA and scatter plot analyses revealed that gastrointestinal symptoms did not occur independently but instead grouped into distinct patterns. Diarrhea, weight loss/anorexia, and GI toxicity clustered together, suggesting a shared pathway, possibly related to mucosal injury, epithelial turnover, and altered nutrient absorption. In contrast, nausea and abdominal pain loaded more independently, reflecting different mechanisms such as central nervous system effects or treatment-related abdominal stress. Constipation emerged as a distinct phenomenon, largely confined to combination settings.

These patterns provide insights into the pathophysiology of sorafenib-induced toxicity and may guide targeted management strategies. For instance, clustering of diarrhea with weight loss and GI toxicity suggests that addressing diarrhea proactively may also mitigate downstream nutritional compromise and mucosal injury. Conversely, nausea and abdominal pain may require separate supportive interventions.

From a clinical perspective, our findings emphasize the need for systematic monitoring and proactive management of GI AEs in patients receiving sorafenib. Diarrhea and anorexia, in particular, have major implications for patient quality of life, nutritional status, treatment adherence, and overall clinical outcomes, and may necessitate dose modification or treatment interruption if inadequately managed. Early recognition through structured monitoring protocols may allow early supportive interventions—this, in turn, may reduce symptom severity and prevent downstream complications such as dehydration, weight loss, and overall functional health decline.

While diarrhea was the most frequent gastrointestinal AE in our meta-analysis, increasing evidence suggests that not all events are clinically equivalent. Beyond its base symptomatic burden, the timing of diarrhea onset may carry some prognostic significance. In a cohort of 344 patients, Díaz-González et al. reported that early diarrhea (within 60 days of treatment initiation) was associated with significantly worse survival and may serve as a clinical signal of suboptimal therapeutic benefit, prompting consideration of transition into second-line therapies. These observations highlight diarrhea not only as more than a toxicity requiring management in and of itself but also as a potential biomarker of treatment nonresponse. This distinction is particularly relevant in modern clinical settings, where multiple effective alternative agents are available and early treatment optimization may meaningfully influence long-term patient outcomes.^12^

From a research perspective, the study highlights gaps in the reporting of GI side effects. Although diarrhea was nearly universally documented, other important symptoms such as constipation and anorexia were inconsistently reported, potentially underestimating their clinical impact. Standardization of AE reporting in HCC trials remains an urgent need. Furthermore, the observed differences between monotherapy and combination regimens underscore the importance of including robust safety endpoints in trials testing sorafenib in multimodality contexts.

This meta-analysis has several limitations. First, heterogeneity across studies in design, patient populations, and reporting standards complicates direct comparisons. In particular, grading of AEs (e.g., CTCAE severity categories) was inconsistently reported and subject to discrepancies in definitions and reporting thresholds, limiting the feasibility of comparative analyses of toxicity severity; therefore, we focused on toxicity prevalence as the most consistently reported outcome across trials. Second, selective reporting remains a challenge: some adverse effects may be underrepresented if not actively monitored. Third, the lack of individual patient-level data prevents stratification by duration of treatment, prior therapies, or liver function, which may influence toxicity profiles. Finally, prevalence outcomes were summarized using mean and weighted prevalence estimates across study arms, but no variance-based random-effects meta-analysis was performed; therefore, I² was not calculated. Also, the reliance on published studies introduces the possibility of publication bias, particularly for underreported side effects.

Although this study primarily reports the prevalence of gastrointestinal AEs associated with sorafenib, these findings have several implications for clinical practice and future research. The high frequency of these toxicities supports the need for routine and systematic monitoring, as well as timely supportive care and dose adjustment when indicated, to limit treatment-related morbidity and preserve treatment continuity. In the research setting, the results underscore the value of standardized AE reporting, inclusion of time-to-onset analyses, and integration of patient-reported outcomes to improve the interpretability and clinical relevance of safety data. Taken together, these considerations provide a rationale for extending descriptive prevalence data toward more structured approaches to toxicity management and study design.

In summary, gastrointestinal side effects are common and clinically significant in sorafenib-treated HCC patients, with diarrhea as the predominant symptom, followed by nausea, abdominal pain, and anorexia/weight loss. Multivariate analyses reveal clustering of diarrhea, weight loss, and GI toxicity, while constipation appears confined to combination regimens. Compared with TACE, sorafenib demonstrates a distinct toxicity profile reflective of systemic rather than locoregional mechanisms. These findings underline the importance of proactive GI toxicity management, standardized AE reporting, and careful evaluation of combination regimens to optimize patient outcomes.

## Conclusion

In conclusion, this meta-analysis synthesizes the available evidence on gastrointestinal AEs associated with sorafenib in patients with HCC, confirming diarrhea as the most frequently reported toxicity. Beyond quantifying incidence, these findings highlight the need for consistent AE reporting and the implementation of systematic, proactive management strategies aimed at minimizing treatment-related morbidity, preserving treatment adherence, and ultimately optimizing clinical outcomes.

## Supplemental Material

sj-docx-5-tam-10.1177_17588359261442686 – Supplemental material for Prevalence of gastrointestinal side effects in hepatocellular carcinoma patients receiving sorafenib: a meta-analysis of 136 studies and 14,416 patients Supplemental material, sj-docx-5-tam-10.1177_17588359261442686 for Prevalence of gastrointestinal side effects in hepatocellular carcinoma patients receiving sorafenib: a meta-analysis of 136 studies and 14,416 patients by Nathalie Arendt, Tania Payo-Serafín, Anna Kolosenko, Maria Kopsida, Markus Sjöblom and Femke Heindryckx in Therapeutic Advances in Medical Oncology

sj-pdf-1-tam-10.1177_17588359261442686 – Supplemental material for Prevalence of gastrointestinal side effects in hepatocellular carcinoma patients receiving sorafenib: a meta-analysis of 136 studies and 14,416 patients Supplemental material, sj-pdf-1-tam-10.1177_17588359261442686 for Prevalence of gastrointestinal side effects in hepatocellular carcinoma patients receiving sorafenib: a meta-analysis of 136 studies and 14,416 patients by Nathalie Arendt, Tania Payo-Serafín, Anna Kolosenko, Maria Kopsida, Markus Sjöblom and Femke Heindryckx in Therapeutic Advances in Medical Oncology

sj-pdf-2-tam-10.1177_17588359261442686 – Supplemental material for Prevalence of gastrointestinal side effects in hepatocellular carcinoma patients receiving sorafenib: a meta-analysis of 136 studies and 14,416 patients Supplemental material, sj-pdf-2-tam-10.1177_17588359261442686 for Prevalence of gastrointestinal side effects in hepatocellular carcinoma patients receiving sorafenib: a meta-analysis of 136 studies and 14,416 patients by Nathalie Arendt, Tania Payo-Serafín, Anna Kolosenko, Maria Kopsida, Markus Sjöblom and Femke Heindryckx in Therapeutic Advances in Medical Oncology

sj-pdf-3-tam-10.1177_17588359261442686 – Supplemental material for Prevalence of gastrointestinal side effects in hepatocellular carcinoma patients receiving sorafenib: a meta-analysis of 136 studies and 14,416 patients Supplemental material, sj-pdf-3-tam-10.1177_17588359261442686 for Prevalence of gastrointestinal side effects in hepatocellular carcinoma patients receiving sorafenib: a meta-analysis of 136 studies and 14,416 patients by Nathalie Arendt, Tania Payo-Serafín, Anna Kolosenko, Maria Kopsida, Markus Sjöblom and Femke Heindryckx in Therapeutic Advances in Medical Oncology

sj-pdf-4-tam-10.1177_17588359261442686 – Supplemental material for Prevalence of gastrointestinal side effects in hepatocellular carcinoma patients receiving sorafenib: a meta-analysis of 136 studies and 14,416 patients Supplemental material, sj-pdf-4-tam-10.1177_17588359261442686 for Prevalence of gastrointestinal side effects in hepatocellular carcinoma patients receiving sorafenib: a meta-analysis of 136 studies and 14,416 patients by Nathalie Arendt, Tania Payo-Serafín, Anna Kolosenko, Maria Kopsida, Markus Sjöblom and Femke Heindryckx in Therapeutic Advances in Medical Oncology

## Declarations

**Ethics approval and consent to participate:** Not applicable.

**Consent for publication:** Not applicable.

**Availability of data and materials:** All data are available as Supplemental Tables.
