# Supplementary material for: Prevalence of gastrointestinal side effects in hepatocellular carcinoma patients receiving sorafenib: a meta-analysis of 136 studies and 14,416 patients
Source: Ther Adv Med Oncol. 2026 May 13;18:17588359261442686. doi: 10.1177/17588359261442686 (PMC13180223; doi:10.1177/17588359261442686)
Supplement: sj-pdf-1-tam-10.1177_17588359261442686 – Supplemental material for Prevalence of gastrointestinal side effects in hepatocellular carcinoma patients receiving sorafenib: a meta-analysis of 136 studies and 14,416 patients [file sj-pdf-1-tam-10.1177_17588359261442686.pdf]

ID-number	PMID	Title	Authors	Study Design	Country	Sample size	median age	% stage A	% stage B	% stage C	Daily dose (mg)	Combination treatment?	Combination treatments	diarrhea	Nausea	abdominal pain	GI toxicity	weight loss	Constipation
1	38900435	Survival in Patients With Recurrent Int	Fan W, Zhu B, (	RCT	China	81	54				800	Yes	TACE	46.5	25.9	45.7	NA	37	NA
2	38568599	Adjuvant Transarterial Chemoemboliza	Peng Z, Fan W,	RCT	China	79	55				800	Yes	TACE	60.7594937	20.2531646	16.4556962	NA	16.4556962	7.6
2	38568599	Adjuvant Transarterial Chemoemboliza	Peng Z, Fan W,	RCT	China	79	52				800	No		62.0253165	15.1898734	12.65822785	NA	13.92405063	8.86
3	38364832	Cabozantinib plus atezolizumab versus	Yau T, Kaseb A,	RCT	worldwide	207	64		33	67	800	No		47.3429952	14.0096618	20.28985507	5.797101449	21.51898734	11.11111111
4	37796513	Tislelizumab vs Sorafenib as First-Line	Qin S, Kudo M,	RCT	worldwide	332	60		24.1	75.9	800	No		39.2	NA	NA	NA	11.1	NA
5	37499670	Camrelizumab plus rivoceranib versus	Qin S, Chan SL,	RCT	worldwide	271	56		15	85	800	no		38.7453875	5.16605166	NA	0	14.39114391	NA
6	37188895	A Phase I Study to Investigate the Safe	Okusaka T, Mo	Phase I	Japan	6	72				800	Yes	Napabucasin	83.3	NA	NA	NA	NA	33.3
7	37038986	Irradiation stent with 125 I plus TACE	Lu J, Guo JH, Ji	RCT	China	54					800	Yes	TACE	10	40.7407407	12.96296296	6	10	10
8	38319892	Tremelimumab plus Durvalumab in Ur	Abou-Alfa GK, I	RCT	worldwide	389	64		17	83	800	No		44.7	14.2	16.8	NA	NA	9.4
9	35798016	Cabozantinib plus atezolizumab versus	Kelley RK, Rima	RCT	worldwide	207	64		34	66	800	No		47	14	20	6	14	11
10	35103539	Sorafenib Plus Hepatic Arterial Infusio	Zheng K, Zhu X,	RCT	China	32	56				800	Yes	Hepatic Arterial Infusion Che	59	22	NA	3	NA	NA
10	35103539	Sorafenib Plus Hepatic Arterial Infusion Chemotherapy		RCT	China	32	55				800	No		41	19	NA	0	NA	NA
11	34982369	GALNT14 genotype-guided chemoemb	Chen WT, Lin S	RCT	China	29	67				400	Yes	TACE	20.7	NA	NA	6.9	20.7	NA
12	16908937	Phase II study of sorafenib in patients	Abou-Alfa GK, S	Phase II	worldwide	137	69				800	No		43.1	16.1	NA	10.9	19	NA
13	17953709	Phase I study of sorafenib in Japanese	Furuse J, Ishii H	Phase I	Japan	13	69				400	No		61.5384615	NA	NA	0	23.07692308	NA
13	17953709	Phase I study of sorafenib in Japanese	Furuse J, Ishii H	Phase I	Japan	14	70				800	No		50	NA	NA	21.42857143	35.71428571	NA
14	18650514	Sorafenib in advanced hepatocellular c	Llovet JM, Ricci	RCT	worldwide	297	64.9		18	82	800	No		39	11	8	NA	14	NA
15	19095497	Efficacy and safety of sorafenib in pati	Cheng AL, Kang	RCT	Asia-Pacific	149	51			95.3	800	No		25.5033557	11.409396	NA	2.684563758	NA	NA
16	19101137	Combination of sorafenib and doxorub	Richly H, Schult	Phase I	Germany	18	55.5				800	Yes	Doxorubicin	16.6666667	NA	NA	NA	NA	NA
17	19107763	Phase 2 open-label study of single-age	Yau T, Chan P, I	Phase II	Hong Kong	51	56				800	No		66.6666667	19.61	23.52941176	11.76470588	NA	NA
18	34914889	Nivolumab versus sorafenib in advance	Yau T, Park JW,	RCT	worldwide	363	65	5	17	78	800	No		47.11	11.29	NA	NA	11.02	NA
19	34905388	Arterial Chemotherapy of Oxaliplatin F	Lyu N, Wang X,	RCT	China	129	53		6.8	93.2	800	No		37.98	21.71	13.95	4.65	30.23	17.05
20	34407972	Upregulation of C/EBPα Inhibits Suppr	Hashimoto A, S	Phase Ib	worldwide	4	62				800	Yes (coadmin.)	MTL-CEBPA 90 mg/m2	75	0	0	0	25	NA
20	34407972	Upregulation of C/EBPα Inhibits Suppr	Hashimoto A, S	Phase Ib	worldwide	18	65				800	Yes (coadmin.)	MTL-CEBPA 130 mg/m2	33.33	5.56	5.56	22.22	0	NA
20	34407972	Upregulation of C/EBPα Inhibits Suppr	Hashimoto A, S	Phase Ib	worldwide	14	66.5				800	Yes (sequential admin.)	MTL-CEBPA 130 mg/m2	42.86	14.29	14.29	28.57	14.29	NA
21	34297268	Priming of Sorafenib Prior to Radiofre	Bockorny B, Bu	RCT	USA	9	69.4				800	No		31.3	50	43.6	9.4	11.11	NA
22	34237154	Transarterial chemoembolization plus	Ding X, Sun W,	RCT	China	32	56				800	Yes	TACE	31.3	50	43.6	NA	15.6	NA
23	34236269	The Impact of Sorafenib in Combinatic	Kaibori M, Mat	Phase II	Japan	38	67				400	Yes	Cisplatin (HAIC)	NA	2.63	NA	NA	2.6	NA
24	34185551	Donafenib Versus Sorafenib in First-Li	Qin S, Bi F, Gu	RCT	China	332	53		12	88	800	No		47.29	NA	NA	NA	10.84	NA
25	34143971	Sintilimab plus a bevacizumab biosimi	Ren Z, Xu J, Bai	RCT	China	185	54		14	86	800	No		42	10	14	2	17	NA
26	33972742	Randomised Phase 1b/2 trial of tepoti	Ryoo BY, Cheng	RCT	worldwide	44	54		5.4	94.6	800	No		31.8	NA	NA	NA	NA	NA
27	33655688	Potential of novel colchicine dosage sc	Lin ZY, Yeh ML,	Phase Iia	Taiwan	86	62					no		46.46	17.44	3.49	NA	NA	NA
28	33481328	Efficacy and safety of sorafenib plus vi	Haruna Y, Yaku	RCT	Japan	22	72		27	73	400	Yes	Vitamin K	18	NA	NA	0	NA	NA
28	33481328	Efficacy and safety of sorafenib plus vi	Haruna Y, Yaku	RCT	Japan	22	71.9		41	59	400	No		9	NA	NA	14	NA	NA
29	32841541	Phase II trial of sorafenib and doxorubi	El Dika I, Capar	Phase II trial	USA	30	65				800	Yes	Doxorubicin	53	67	NA	33	30	NA
30	32776632	A Phase I Trial of Trametinib in Combi	Kim R, Tan E, V	Phase I	USA	17	65		12	88	400	Yes	Trametinib	83	76	NA	NA	NA	NA
31	32548867	Phase Ib Study of Enzalutamide with o	Harding JJ, Kell	Phase I	USA	12	62					Yes	Enzalutamide	25	25	42	17	25	25
32	32402160	Atezolizumab plus Bevacizumab in Un	Finn RS, Qin S,	Phase III	worldwide	165	66	4	16	81	800	No		49.4	16	17.3	NA	9.6	14.1
33	31070690	Sorafenib Plus Hepatic Arterial Infusio	He M, Li Q, Zou	RCT	China	124	49				800	Yes	HAIC of FOLFOX (oxiplatin, le	29.0322581	79.8387097	35.48387097	7.258064516	35.48387097	NA
33	31070690	Sorafenib Plus Hepatic Arterial Infusio	He M, Li Q, Zou	RCT	China	121	49				800	No		42.1487603	28.9256198	9.917355372	7.438016529	32.23140496	NA
34	30944458	Sorafenib alone vs. sorafenib plus GEN	Assenat E, Pag	RCT	France	39	65		10	90	800	Yes	Gemcitabine + oxalplatin	82.0512821	89.7435897	NA	NA	NA	NA
34	30944458	Sorafenib alone vs. sorafenib plus GEN	Assenat E, Pag	RCT	France	44	62		18	82	800	No		75	93.1818182	NA	NA	NA	NA
35	30587616	β-Hydroxy-β-methyl Butyrate/L-Arginir	Naganuma A, Hoshino T, Ohno N		Japan	25	72.4				800	Yes	Oral nutritional supplement	28	NA	NA	NA	NA	NA
35	30587616	β-Hydroxy-β-methyl Butyrate/L-Arginir	Naganuma A, Hoshino T, Ohno N		Japan	25	68.2				800	No		56	NA	NA	NA	NA	NA
36	30529387	Sorafenib with or without concurrent t	Park JW, Kim Y	RCT	Korea	153	60.2	1.8	22.9	75.3	600	Yes	cTACE	39.2156863	37.9084967	53.59477124	NA	NA	21.56862745
36	30529387	Sorafenib with or without concurrent t	Park JW, Kim Y	RCT	Korea	167	61.3	0	26	74	600	No		32.3353293	8.98203593	17.36526946	NA	NA	11.9760479
37	30198057	Phase I/II study of first-line combinati	Tak WY, Ryoo B	RCT	worldwide	84	65		24.1	75.9	800	Yes	Resminostat	54.7619048	57.1428571	NA	NA	20.23809524	NA
37	30198057	Phase I/II study of first-line combinati	Tak WY, Ryoo B	RCT	worldwide	84	62		23.8	76.2	800	No		46.4285714	14.2857143	NA	NA	14.28571429	NA
38	30190369	A Phase II and Biomarker Study of Sora	Goyal L, Zheng	Phase II	USA	39	65		5	95	800	Yes	FOLFOX	58.974359	53.8461538	10.25641026	7.692307692	29	23.07692308
39	29995286	A phase 1b study of transforming grov	Ikeda M, Morin	Phase Ib	Japan	7	68				800	Yes	Galunisertib	28.5714286	14.2857143	NA	14.29	29	14.28571429
39	29995286	A phase 1b study of transforming grov	Ikeda M, Morin	Phase Ib	Japan	7	67				800	Yes	Galunisertib	14.2857143	28.5714286	NA	14.29	NA	14.28571429
40	29982870	Randomized, prospective, comparative	Choi JH, Chung	RCT	Korea	29	60.2				800	No		17.2413793	NA	NA	NA	3.45	NA
41	29950351	Phase II Studies with Refametinib or R	Lim HY, Merle I	Phase II	worldwide	16	67	12.5	12.5	75	800	Yes	Rafametinib	62.5	12.5	12.5	31.3	25	18.75
42	29948358	Survival and tolerance to sorafenib in (	Leal CRG, Magalhães C, Barbosa		Brazil	130	61.7		12.3	87.7	800	No		60.7692308	NA	NA	NA	96.8	NA
43	29934260	Multicenter Phase II Clinical Trial of Sc	Sato Y, Nishiof	Phase II	Japan	31	75				800	Yes	TACE	45.1612903	41.9354839	58.06451613	NA	NA	NA
44	29719302	A Randomized Phase II Open-Label Mu	Thomas MB, G	RCT	USA	43	61	9	26	65	800	No		69.7674419	41.8604651	23.25581395	NA	39.53488372	23.25581395
45	29631810	Sorafenib plus low-dose cisplatin and	Kudo M, Ueshir	RCT	Japan	88	69		31	69	800	Yes	HAIC (cisplatin + fluorouracil)	34.0909091	38.6363636	NA	NA	39.77272727	NA
45	29631810	Sorafenib plus low-dose cisplatin and	Kudo M, Ueshir	RCT	Japan	102	68		26	74	800	No		49.0196078	16.6666667	NA	NA	33.33333333	NA
46	29563636	A multicentre, open-label, phase-I/ran	Palmer DH, Ma	Phase I + 2	Europe	31	64		22.6	74.2	800	No		67.7	29	29	NA	NA	NA
47	29552783	Sunitinib versus sorafenib plus transar	Xu Q, Huang Y,	RCT	China	53	62	9.4	30.2	60.4	800	Yes	TACE	45	47	72	32	15	45
48	29520435	A phase I trial of escalating doses of c	El-Khoueiry AB,	Phase I	USA	21	61				800	Yes	Cixutumumab	62	29	NA	NA	24	NA
49	29498924	SIRveNIB: Selective Internal Radiation	Chow PKH, Gar	Phase III	Worldwide	162	57.5	0.6	54.3	45.1	800	No		29.6	6.2	6.8	1.9	NA	5.6
50	29474553	A multicenter Phase II study of sorafer	Suzuki E, Kanek	Phase II	Japan	40	69				800	No		37.5	10	NA	7.5	NA	NA
50	29474553	A multicenter Phase II study of sorafer	Suzuki E, Kanek	Phase II	Japan	12	66				800	No		58.3	25	NA	0	NA	NA
51	29433850	Lenvatinib versus sorafenib in first-line	Kudo M, Finn R	Phase III	worldwide	475	62		19	81	800	No		46	14	18	NA	22	11
52	29209114	Combined endovascular brachytherapy	Zhang ZH, Liu C	Retrospective	China	37					800	Yes	EVBT, stent, TACE	70.2702703	NA	NA	NA	NA	NA
52	29209114	Combined endovascular brachytherapy	Zhang ZH, Liu C	Retrospective	China	31					800	Yes	TACE	61.2903226	NA	NA	25.8	NA	NA
53	29107679	Efficacy and safety of selective interna	Vilgrain V, Pere	Phase III	France	222	65	5	27	67	800	No		77.3	24	32.4	6	21.3	NA
54	28648803	Sorafenib in combination with transar	Meyer T, Fox R	Phase III	UK	157	65				800	Yes	TACE	59.2	45.8	59.2	14.6	12.1	14.6
55	28592620	Phase II Study of First-Line Trebananib	Abou-Alfa GK, I	Phase II	USA	60	64				800	Yes	Trebananib 10 mg/kg	20	NA	NA	NA	NA	NA
56	28573606	A Phase I Study of Combination Therap	Sho T, Nakanisl	Phase I	Japan	12	65				800	Yes	5-FU	0	0	NA	33.3	NA	NA
57	28465443	Phase I and Preliminary Phase II Study	Duffy AG, Ma C	Phase I+II	USA	25	60				800	Yes	TRC105	60	56	36	48	32	12
58	28272913	Phase I Study of Sorafenib in Combina	Ishizaki M, Kaik	Phase I	Japan	3	71.8				400	Yes	HAIC	66.6666667	0	NA	NA	NA	NA
58	28272913	Phase I Study of Sorafenib in Combina	Ishizaki M, Kaik	Phase I	Japan	3	71.8				400	Yes	HAIC	33.3333333	0	NA	NA	NA	NA
58	28272913	Phase I Study of Sorafenib in Combina	Ishizaki M, Kaik	Phase I	Japan	6	71.8				400	Yes	HAIC	16.6666667	16.6666667	NA	NA	NA	NA
58	28272913	Phase I Study of Sorafenib in Combina	Ishizaki M, Kaik	Phase I	Japan	3	71.8				800	Yes	HAIC	33.3333333	0	NA	NA	NA	NA

59	28120036	Phase Ib study of codrituzumab in com	Abou-Alfa GK, '	Phase Ib	worldwide	12	56.8				800	Yes	Codrituzumab (2.5 mg/kg qw	0	NA	NA	NA	NA	NA
59	28120036	Phase Ib study of codrituzumab in com	Abou-Alfa GK, '	Phase Ib	worldwide	12	63.8				800	Yes	Codrituzumab (5 mg/kg qw)	0	NA	NA	NA	NA	NA
59	28120036	Phase Ib study of codrituzumab in com	Abou-Alfa GK, '	Phase Ib	worldwide	3	57.7				800	Yes	Codrituzumab (10 mg/kg qw)	0	NA	NA	NA	NA	NA
59	28120036	Phase Ib study of codrituzumab in com	Abou-Alfa GK, '	Phase Ib	worldwide	6	64.5				800	Yes	Codrituzumab (1600 mg q2w	16.6666667	NA	NA	NA	NA	NA
59	28120036	Phase Ib study of codrituzumab in com	Abou-Alfa GK, '	Phase Ib	worldwide	7	57.9				800	Yes	Codrituzumab (1600 mg qw)	0	NA	NA	NA	NA	NA
60	27943153	Phase I/II Randomized Trial of Sorafer	Hubbard JM, M	Phase I	USA	17	66				800/400	Yes	Bevacizumab	NA	5.88235294	NA	5.88	5.882352941	NA
61	27909950	HATT: a phase IV, single-arm, open-lal	Lin SM, Lu SN,	Phase IV	Taiwan	151	62				800	No		45	NA	NA	NA	5.3	NA
62	27793949	Sorafenib Combined with Radio-freque	Giorgio A, Merola MG, Montesarchio L, Merol			49	71				800	Yes	RFA-MPVTT	12.244898	NA	14.28571429	NA	20.40816327	NA
62	27793949	Sorafenib Combined with Radio-freque	Giorgio A, Merola MG, Montesarchio L, Merol			50	72				800	No		12	NA	14	NA	16	NA
63	27573564	Sorafenib plus hepatic arterial infusior	Ikeda M, Shimi	Phase II	Japan	65	66		29.2	70.8	800	Yes	HAIC (cisplatin)	35.3846154	41.5384615	NA	NA	NA	NA
63	27573564	Sorafenib plus hepatic arterial infusior	Ikeda M, Shimi	Phase II	Japan	41	64		39	61	800	No		41.4634146	19.5121951	NA	NA	NA	NA
64	27755109	Real-life experience with sorafenib for	Merchante N, Ibarra S, Revollo B		Spain	44	50	7	14	68	800	No		29.5454545	NA	NA	NA	NA	NA
65	27681866	A phase Ib study of selumetinib (AZD6	Tai WM, Yong '	Phase Ib	Singapore	18	63		25.9	74.1	800	Yes	Selumetinib (75 mg)	88.8888889	NA	11.11111111	NA	NA	11.11111111
65	27681866	A phase Ib study of selumetinib (AZD6	Tai WM, Yong '	Phase Ib	Singapore	6	63		25.9	74.1	800	Yes	Selumetinib (50 mg)	83.3333333	NA	50	NA	NA	0
65	27681866	A phase Ib study of selumetinib (AZD6	Tai WM, Yong '	Phase Ib	Singapore	3	63		25.9	74.1	800	Yes	Selumetinib (100 mg)	66.6666667	NA	0	NA	NA	0
66	27256874	Phase I Study of Lenalidomide and Sor	Shahda S, Loeh	Phase I	USA	5	56				800	Yes	Lenalidomide	20	60	NA	NA	NA	NA
67	22033636	Phase II trial of sorafenib in combinati	Petrini I, Lencic	Phase II	Italy	39	67		24	64	800	Yes	5-Fluorouracil	58.974359	33.3333333	NA	46.2	NA	NA
68	21941188	A single center experience of sorafenil	Song T, Zhang W, Wu Q, Kong D,		China	40	56		52.5	47.5	800	No		52.5	NA	NA	NA	NA	NA
69	21932373	Efficacy and safety of sorafenib in cor	Gomez-Martin C, Bustamante J,		Spain	31	53.6				800	Yes	Everolimus or sirolimus	77.4193548	6.4516129	NA	6.451612903	NA	NA
70	21911714	Phase II trial of sorafenib combined wi	Pawlik TM, Rey	Phase II	USA	35	63		34	64	800	No		8.57142857	11.4285714	5.714285714	2.857142857	NA	11.42857143
70	21911714	Phase II trial of sorafenib combined wi	Pawlik TM, Rey	Phase II	USA	33	63		34	64	800	Yes	DEB-TACE	30.3030303	33.3333333	27.27272727	3.03030303	3.03030303	45.45454545
70	21911714	Phase II trial of sorafenib combined wi	Pawlik TM, Rey	Phase II	USA	28	63		34	64	800	Yes	DEB-TACE	21.4285714	17.8571429	39.28571429	7.142857143	10.71428571	25
71	21695438	Phase 1 trial of S-1 in combination wit	Lee SJ, Lee J, P	Phase I		3	48				800	Yes	S-1 (50 mg/m2/day)	66.6666667	0	66.66666667	33.33333333	0	0
71	21695438	Phase 1 trial of S-1 in combination wit	Lee SJ, Lee J, P	Phase I		4	48				800	Yes	S-1 (60 mg/m2/day)	50	25	25	50	25	25
71	21695438	Phase 1 trial of S-1 in combination wit	Lee SJ, Lee J, P	Phase I		6	48				800	Yes	S-1 (70 mg/m2/day)	50	0	33.33333333	16.66666667	0	16.66666667
71	21695438	Phase 1 trial of S-1 in combination wit	Lee SJ, Lee J, P	Phase I		7	48				800	Yes	S-1 (80 mg/m2/day)	0	28.5714286	28.57142857	57.14285714	0	14.28571429
72	21664811	Phase III study of sorafenib after trans	Kudo M, Imana	Phase III	worldwide	229	69				800	No		31	NA	NA	NA	NA	NA
73	21340026	Reversible decrease of portal venous f	Coriat R, Gouya H, Mir O, Ropert S, Vignaux O			7	62.8	71.4285714	28.5714286	0	800	No		NA	NA	NA	NA	NA	NA
74	21081728	Doxorubicin plus sorafenib vs doxorubi	Abou-Alfa GK, .	Phase II	worldwide	47	66				800	Yes	Doxorubicin	10.6382979	6.38297872	6.382978723	NA	NA	NA
75	21036880	Continuous administration of sorafenil	Dufour JF, Hopp	Phase I	Switzerland	14	63.5		64	36	800	Yes	TACE	50	NA	28.57142857	NA	71.42857143	NA
76	20416968	Phase II study of combining sorafenib	Hsu CH, Shen Y	Phase II	Taiwan	53	57		6	94	800	Yes	Tegafur/uracil	49.0566038	13.2075472	NA	19	NA	NA
77	20041325	Sorafenib plus octreotide is an effectiv	Prete SD, Mont	Phase II		50	67.5		42	58	800	Yes	Octreotide	32	NA	4	6	NA	NA
78	24312711	Practical effect of sorafenib monother	Jeong SW, Jang	Phase I	Korea	30	57			100	800	No		20	6.7	NA	3.3	NA	NA
79	24081937	Sunitinib versus sorafenib in advanced	Cheng AL, Kang	Phase III	Worldwide	544	59		16	84	800	No		47.2	17.3	19.9	9.8	20.7	14.8
80	23980084	Brivanib versus sorafenib as first-line t	Johnson PJ, Qir	Phase III	worldwide	575	60	5	17	78	800	No		50	19	32	NA	21	16
81	23928403	Phase I study investigating everolimus	Finn RS, Poon F	Phase I	worldwide	16	57.5			100	800	Yes	Everolimus 2.5mg	62.5	18.8	50	18.8	NA	25
81	23928403	Phase I study investigating everolimus	Finn RS, Poon F	Phase I	worldwide	14	62.5		14.3	85.7	800	Yes	Everolimus 5.0mg	71.4	21.4	14.3	42.9	NA	35.7
82	23824645	Sorafenib versus capecitabine in the m	Abdel-Rahman	Phase II	Egypt	26	53.5			100	800	No		30	NA	NA	3	NA	NA
83	23749944	Phase I adjuvant trial of sorafenib in p	Jia N, Liou I, Ha	Phase I	USA	4	57				400-800	No		25	NA	NA	25	NA	NA
84	23519998	Temsirolimus combined with sorafenil	Kelley RK, Nim	Phase I	USA	25	60		16	84	400	Yes	Temsirolimus	48	28	NA	28	52	NA
85	23431262	Sorafenib in advanced hepatocellular c	Køstner AH, So	Retrospective	Denmark	76					800	No		42	32	NA	4	47	NA
86	23324079	Sorafenib in combination with transar	Bai W, Wang Y	Prospective	China	82	54		23.2	76.8	800	Yes	TACE	36.6	NA	NA	7.3	NA	NA
87	23263829	Sorafenib in hepatocellular carcinoma	Brunocilla PR, I	Prospective	Italy	36	67.4		8.3	91.7	400-800	no		52.8	36.1	38.9	2.8	25	NA
88	23041587	Sorafenib in patients with Child-Pugh (	Pressiani T, Bo	Prospective	Italy	297	68	78.8	21.2		800-1200	No		53	20.9	29.3	30.6	50.2	NA
89	22524575	The efficacy of hepatic arterial infusio	Jeong SW, Jang	Phase I	Korea	20	59.5			100	800	No		35	25	NA	10	NA	NA
90	22334456	Transarterial chemoembolization plus	Sansonno D, La	RCT	Italy	40	73			100	800	No		10	17.5	NA	NA	NA	NA
91	22314421	Phase II study of concurrent transarter	Park JW, Koh Y	Phase II	Korea	50	61.5		82	12	400	Yes	TACE	48	54	64	54	NA	16
92	22215073	Conventional transarterial chemoembo	Sieghart W, Pir	Pilot	Austria	15	67	6	60	33.3	800	Yes	TACE	46.7	53.3	93.3	20	86.7	NA
93	26952006	Resminostat plus sorafenib as second-	Bitzer M, Horg	Phase I/II	Germany + It	26	67		19	81	400	Yes	resminostat	42	46	15	NA	27	NA
94	26884590	Sorafenib with or without everolimus i	Koeberle D, Du	RCT	worldwide	59	66		25.4237288	74.5762712	800	Yes	Everolimus	64	NA	NA	39	56	NA
94	26884590	Sorafenib with or without everolimus i	Koeberle D, Du	RCT	worldwide	46	65		30.4347826	69.5652174	800	No		48	NA	NA	20	59	NA
95	26867886	Phase 1 Trial of Sorafenib and Stereot	Brade AM, Ng	Phase I	Canada	16	61.5		25	75	200-800	Yes	SBRT	NA	NA	NA	18.75	NA	NA
96	26809111	Sorafenib or placebo plus TACE with d	Lencioni R, Llo	RCT	Worldwide	154	64.5				800	Yes	TACE	52.9	37.9	60.1	20.9	20.3	19
97	26802147	A randomized, double-blind, placebo-c	Ciuleanu T, Baz	RCT	Worldwide	51	64		29.4	70.6	800	No		37	NA	NA	NA	43	NA
97	26802147	A randomized, double-blind, placebo-c	Ciuleanu T, Baz	RCT	Worldwide	50	60		28	72	800	Yes	mapatumuab	32	NA	NA	NA	38	NA
98	26446238	Sorafenib With and Without Transarte	Zhang Y, Fan W	retrospective ana	China	43	50.1				800	Yes	TACE	44.4	NA	NA	NA	33.3	NA
98	26446238	Sorafenib With and Without Transarte	Zhang Y, Fan W	retrospective ana	China	44	53.6				800	No		43.2	NA	NA	NA	34.1	NA
99	26361969	Adjuvant sorafenib for hepatocellular (	Bruix J, Takaya	RCT	worldwide	556	58				800	No		43	9	10	NA	11	7
100	26071796	Safety and efficacy of tigatuzumab plu	Cheng AL, Kang	Phase II	worldwide	53	63				800	Yes	tigatuzumab	44.2	19.2	26.9	NA	NA	36.5
100	26071796	Safety and efficacy of tigatuzumab plu	Cheng AL, Kang	Phase II	worldwide	54	62.5				800	Yes	tigatuzumab	65.5	20	30.9	NA	NA	23.6
100	26071796	Safety and efficacy of tigatuzumab plu	Cheng AL, Kang	Phase II	worldwide	55	66				800	No		49.1	14.5	21.8	NA	NA	16.4
101	25957784	Impact of neo-adjuvant Sorafenib trea	Hoffmann K, G	RCT	Germany	24	58.5				800	yes	TACE	37.5	12.5	NA	NA	4.2	NA
102	26069923	Open-Label Single-Arm Phase II Trial c	Cosgrove DP, R	Phase II	USA	50	60	6	32	62	800	Yes	TACE	38	48	NA	6	NA	NA
103	26989044	Concurrent sorafenib therapy extends	Yao X, Yan D, Z	(non randomized	China	50	56.5		42	58	800	Yes	TACE	38	NA	NA	14	NA	NA
104	27082062	Randomized, open-label phase 2 study	Cheng AL, Thor	RCT	worldwide	83	56		2	98	800	No		42	19	20	11	20	14
105	26644411	A Phase I Study of the Safety, Pharmac	Adjei AA, Richa	Phase I	worldwide	19	63.7				800	Yes	refametinib	84.2	52.6	26.3	26.3	42	NA
106	25683938	Sorafenib combined with percutaneou	Kan X, Jing Y, V	RCT	China	30	53.7		26.7	73.3	400	Yes	RFA	46.7	NA	NA	NA	NA	NA
107	25583146	[Therapeutic decisions in the treatmer	Turnes J, Díaz F	Non-randomized	Spain	702	64.6	28.5	39.7	31.8	800	No		35.4	16.8	6.8	NA	NA	NA
108	25547503	SEARCH: a phase III, randomized, doub	Zhu AX, Rosmo	Phase III	worldwide	362	60.5		16.6	83.4	800	Yes	Erlotinib	76.2	24.3	32	NA	34.8	12.7
108	25547503	SEARCH: a phase III, randomized, doub	Zhu AX, Rosmo	Phase III	worldwide	355	60		13.4	86.6	800	No		59.4	31	32.4	NA	30.7	20
109	25488963	Linifanib versus Sorafenib in patients v	Cainap C, Qin S	RCT Phase III	worldwide	521	60		19.6	80.4	800	No		9.2	0.8	2.7	NA	NA	NA
110	25294897	A phase II study of the efficacy and saf	Lim HY, Heo J,	Phase II	Asia	70	55.4				600-800	Yes	Refametinib	77.1	47.1	31.4	4.3	NA	NA
111	25294187	Phase 1 trial of tivantinib in combinati	Puzanov I, Sosr	Phase I	USA/Italy	20	61.9				200-400	Yes	Tivantinib	40	25	NA	NA	NA	NA
112	25173458	TACE plus sorafenib for the treatment	Erhardt A, Kolli	Phase II	Germany	43	69	2	77	21	525 (100-800)	Yes	TACE	44.2	11.6	4.7	NA	9.3	NA
113	25099027	The combination of transcatheter arte	Chao Y, Chung	Phase II	Asia			16.9	81.5	1.6	800	Yes	TACE	25	NA	2.1	34.9	NA	NA
114	24977690	Randomized Phase II Study of the X-lir	Lee FA, Zee BC	Phase II	China	31	61				800	Yes	AEG35136	6.5	NA	NA	NA	NA	NA
114	24977690	Randomized Phase II Study of the X-lir	Lee FA, Zee BC	Phase II	China	17	54				800	No		5.9	NA	NA	NA	NA	NA

115	24930619	Safety and toxicity of radioembolizatic	Ricke J, Bulla K	Phase III	Europe	20	68.5		30	70	557	No		55	10	NA	NA	68	NA
115	24930619	Safety and toxicity of radioembolizatic	Ricke J, Bulla K	Phase III	Europe	20	71.5		40	60	614	Yes	Radioembolization	55	15	NA	NA	70	NA
116	24894839	Analysis of survival factors in patients	Zheng J, Shao (	Phase III	China	75	51.3		10.7	89.3	800	Yes	TACE	4	NA	NA	8	NA	NA
117	24810940	Efficacy and safety of sorafenib in com	Srimuninnimit	Phase II	Pakistan	45	50.2				800	Yes	Gemcitabine	71	42	NA	NA	NA	NA
118	24793745	Neutrophil-lymphocyte ratio as a predi	Wei K, Wang M	Non-randomized	Taiwan	40	58.7		50	50	800	Yes	TAE	62.5	55	72.5	NA	NA	NA
119	24740650	Safety and efficacy of sorafenib in the	Imedio ER, Bev	Non-randomized	Italy	62	62		17.7	82.3	800	No		37.1	NA	NA	3.2	NA	NA
120	24698672	Sorafenib in liver function impaired ad	Ji YX, Zhang ZF	RCT	China	95	59.4		12	88	800	No		14.6	12.4	NA	NA	NA	14.6
121	24681342	Prospective randomized pilot study of	Kulik L, Vouche	RCT	USA	10	58		10	20	800	Yes	Y90	10	20	NA	NA	NA	NA
122	24661657	Phase 2 study of combined sorafenib a	Chen SW, Lin L(	Phase II	Taiwan	40	60	10	25	65	800	Yes	RT	25	NA	NA	8.4	NA	NA
123	24614178	Multicenter phase II study of sequentia	Chow PK, Poon	Phase II	Asia	29	64,6		38	62	600	Yes	Radioembolization	31	7	NA	NA	NA	NA
124	24599799	A phase I/II study of S-1 with sorafenil	Ooka Y, Chiba T	Phase I/II	Asia	26	65.5	26	26.9	73.1		Yes	S-2	19.2	NA	NA	11.5	NA	NA
125	24438504	Phase I study of combination chemoth	Hagihara A, Ike	Phase I	Japan	20	64.5				800	Yes	Cisplatin	20	NA	NA	10	NA	NA
126	24350564	The feasibility of combined transcathe	Cho JY, Paik YH	Non-randomized	China	35	51			100	800	Yes	TACE+IMRT	20.8	NA	20.8	NA	NA	NA
127	24333135	Efficacy and safety of sorafenib-gemcitabine combina		Phase II	Pakistan	30	55.3				800	Yes	Gemcitabine	20	NA	NA	NA	NA	NA
128	32206991	Metabolic Switch in Hepatocellular Ca	Castello A, Rim	proof-of-concept	Italy	13	69				800			23	NA	NA	NA	NA	NA
129	32084526	Efficacy and Safety of Liver-Directed C	Kim BK, Kim DY	Phase III	Korea	34	56				800	no	radiation therapy, hepatic ar	36.2	17	12.8	4.2	NA	NA
130	31801872	Randomised, multicentre prospective t	Kudo M, Ueshir	multicentre pros	Japan	80	72	33.8	55	11.3	800	yes	TACE	14.3	NA	NA	NA	11.7	NA
131	31615466	Randomized, phase II trial of sequentia	Kondo M, Morii	Phase II	Japan	33	70.9	6.1	39.4	54.4	800	no		3	0	0	NA	3	NA
131	31615466	Randomized, phase II trial of sequentia	Kondo M, Morii	Phase II	Japan	23	72	5.7	40	54.3	800	yes	hepatic arterial infusion chen	0	0	4	NA	0	NA
132	31429027	Efficacy and Safety of Bavituximab in (	Mokdad AA, Zh	Phase II	USA	38	61		5	95	800	yes	bavituximab	32	8	18	13	NA	NA
133	31305287	Phase I Study of Sorafenib and Vorinos	Gordon SW, Mc	Phase I	USA	10	65				800/400	yes	Vorinostat	37.6	25	NA	6.3	6.3	12.5
134	31295152	A Phase 2 Study of Galunisertib (TGF-[	Kelley RK, Gane	Phase II	worldwide	44	64		40.9	52.3	800	yes	Galunisertib	43.2	18.2	11.4	11.4	22.7	NA
135	31185950	A prospective clinical trial on sorafenil	Eilard MS, And	prospective clinic	Sweden	12	55				800	no		58.3	25	NA	16.7	NA	NA
136	31125576	Pravastatin combination with sorafeni	Jouve JL, Lecon	Phase III	France	162	68				800	yes	pravastatin	11	NA	41.9	93.5	NA	NA
136	31125576	Pravastatin combination with sorafeni	Jouve JL, Lecon	Phase III	France	161	68				800	no		8.9	NA	45.3	90.4	NA	NA
