# Supplementary material for: Prevalence of gastrointestinal side effects in hepatocellular carcinoma patients receiving sorafenib: a meta-analysis of 136 studies and 14,416 patients
Source: Ther Adv Med Oncol. 2026 May 13;18:17588359261442686. doi: 10.1177/17588359261442686 (PMC13180223; doi:10.1177/17588359261442686)
Supplement: sj-pdf-2-tam-10.1177_17588359261442686 – Supplemental material for Prevalence of gastrointestinal side effects in hepatocellular carcinoma patients receiving sorafenib: a meta-analysis of 136 studies and 14,416 patients [file sj-pdf-2-tam-10.1177_17588359261442686.pdf]

Reason for exclusion	Number of studies
Not sorafenib	85
AE not reported	62
Subanalyses previous trial	60
Not HCC	8
Inaccessible	4
Not a clinical trial	7
