# Supplementary material for: Prevalence of gastrointestinal side effects in hepatocellular carcinoma patients receiving sorafenib: a meta-analysis of 136 studies and 14,416 patients
Source: Ther Adv Med Oncol. 2026 May 13;18:17588359261442686. doi: 10.1177/17588359261442686 (PMC13180223; doi:10.1177/17588359261442686)
Supplement: sj-pdf-3-tam-10.1177_17588359261442686 – Supplemental material for Prevalence of gastrointestinal side effects in hepatocellular carcinoma patients receiving sorafenib: a meta-analysis of 136 studies and 14,416 patients [file sj-pdf-3-tam-10.1177_17588359261442686.pdf]

				SCREENING QUESTIONS		2. RANDOMIZED CONTROLLED TRIALS					3. NON-RANDOMIZED STUDIES				
	PMID	Title	Authors	S1. Are there clear research questions?	S2. Do the collected data allow to address the research questions?	2.1. Is randomization appropriately performed?	2.2. Are the groups comparable at baseline?	2.3. Are there complete outcome data?	2.4. Are outcome assessors blinded to the intervention provided?	2.5 Did the participants adhere to the assigned intervention?	3.1. Are the participants representative of the target population?	3.2. Are measurements appropriate regarding both the outcome and intervention (or exposure)?	3.3. Are there complete outcome data?	3.4. Are the confounders accounted for in the design and analysis?	3.5. During the study period, is the intervention administered (or exposure occurred) as intended?
1	38900435	Survival in	Fan W, Zhu B, Chen S,	Yes	Yes	Yes	Yes	Yes	Can't tell	No					
2	38568599	Adjuvant T	Peng Z, Fan W, Liu Z, X	Yes	Yes	Yes	Yes	Yes	Can't tell	Yes					
3	38364832	Cabozantir	Yau T, Kaseb A, Cheng	Yes	Yes	Yes	Yes	Yes	Yes	Yes					
4	37796513	Tislelizuma	Qin S, Kudo M, Meyer	Yes	Yes	Yes	Yes	Yes	No	Can't tell					
5	37499670	Camrelizur	Qin S, Chan SL, Gu S, B	Yes	Yes	Yes	Yes	Yes	No	Yes					
6	37188895	A Phase I S	Okusaka T, Morimoto	Yes	Yes						Yes	Yes	Yes	Can't tell	Yes
7	37038986	Irradiation	Lu J, Guo JH, Ji JS, Li YI	Yes	Yes	Yes	Yes	Yes	No	Yes					
8	38319892	Tremelimu	Abou-Alfa GK, Lau G, K	Yes	Yes	Yes	Yes	Yes	Can't tell	Yes					
9	35798016	Cabozantir	Kelley RK, Rimassa L, C	Yes	Yes	Yes	Yes	Yes	Yes	Yes					
10	35103539	Sorafenib	Plus Hepatic Arterial Inf	Yes	Yes	Yes	Yes	Yes	Yes	Yes					
11	34982369	GALNT14 g	Chen WT, Lin SM, Lee	Yes	Yes	Yes	Yes	Yes	Can't tell	Yes					
12	16908937	Phase II stu	Abou-Alfa GK, Schwar	Yes	Yes						Yes	Yes	Yes	Can't tell	Yes
13	17953709	Phase I stu	Furuse J, Ishii H, Naka	Yes	Yes	Yes	Yes	Yes	Can't tell	Yes					
14	18650514	Sorafenib i	Llovet JM, Ricci S, Maz	Yes	Yes	Yes	Yes	Yes	Yes	Yes					
15	19095497	Efficacy an	Cheng AL, Kang YK, Ch	Yes	Yes	Yes	Yes	Yes	Yes	Yes					
16	19101137	Combinatio	Richly H, Schultheis B,	Yes	Yes						Yes	Yes	Yes	Can't tell	No
17	19107763	Phase 2 op	Yau T, Chan P, Ng KK, C	Yes	Yes						Yes	Yes	Yes	Can't tell	Yes
18	34914889	Nivolumab	Yau T, Park JW, Finn R	Yes	Yes	Yes	Yes	Yes	Yes	No					
19	34905388	Arterial Ch	Lyu N, Wang X, Li JB, L	Yes	Yes	Yes	Yes	Yes	Can't tell	Yes					
20	34407972	Upregulati	Hashimoto A, Sarker D	Yes	Yes						Yes	Yes	Yes	Can't tell	Yes
21	34297268	Priming of	Bockorny B, Bullock AJ	Yes	Yes	Yes	Yes	Yes	Yes	Yes					
22	34237154	Transarteri	Ding X, Sun W, Li W, S	Yes	Yes	Yes	Yes	Yes	Can't tell	No					
23	34236269	The Impact	Kaibori M, Matsushima	Yes	Yes						Yes	Yes	Yes	Can't tell	Yes
24	34185551	Donafenib	Qin S, Bi F, Gu S, Bai Y	Yes	Yes	Yes	Yes	Yes	Yes	Yes					
25	34143971	Sintilimab	Ren Z, Xu J, Bai Y, Xu A	Yes	Yes	Yes	Yes	Yes	Yes	Can't tell					
26	33972742	Randomise	Ryoo BY, Cheng AL, Re	Yes	Yes	Yes	Yes	Yes	No	Can't tell					
27	33655688	Potential o	Lin ZY, Yeh ML, Huang	Yes	Yes						Yes	Yes	Yes	Can't tell	Yes
28	33481328	Efficacy an	Haruna Y, Yakushijin T,	Yes	Yes	Yes	Yes	Yes	Can't tell	Yes					
29	32841541	Phase II tri	El Dika I, Capanu M, Ch	Yes	Yes						Yes	Yes	Yes	Can't tell	Yes
30	32776632	A Phase I T	Kim R, Tan E, Wang E,	Yes	Yes						Yes	Yes	Yes	Can't tell	Yes
31	32548867	Phase Ib St	Harding JJ, Kelley RK, T	Yes	Yes						Can't tell	Yes	Yes	Can't tell	No
32	32402160	Atezolizum	Finn RS, Qin S, Ikeda M	Yes	Yes						Yes	Yes	Yes	Can't tell	Yes
33	31070690	orafenib Al	Wu X, Xu L, Wei W, Le	Yes	Yes	Yes	Yes	Yes	Can't tell	No					
34	30944458	Sorafenib a	Assenat E, Pageaux GF	Yes	Yes	Yes	Yes	Yes	Can't tell	Yes					
35	30587616	β-Hydroxy-	Naganuma A, Hoshino	Yes	Yes						Yes	Yes	Yes	Can't tell	Yes
36	30529387	Sorafenib v	Park JW, Kim YJ, Kim D	Yes	Yes	Yes	Yes	Yes	Can't tell	Yes					
37	30198057	Phase I/II s	Tak WY, Ryoo BY, Lim	Yes	Yes	Yes	Yes	Yes	Can't tell	No					
38	30190369	A Phase II a	Goyal L, Zheng H, Abra	Yes	Yes						Yes	Yes	Yes	Can't tell	no
39	29995286	A phase 1b	Ikeda M, Morimoto M,	Yes	Yes						Yes	Yes	Yes	Can't tell	Yes
40	29982870	Randomize	Choi JH, Chung WJ, Ba	Yes	Yes	Yes	Yes	Yes	Yes	Yes					
41	29950351	Phase II St	Lim HY, Merle P, Weis	Yes	Yes						Yes	Yes	Yes	Can't tell	Yes
42	29948358	Survival an	Leal CRG, Magalhães C	Yes	Yes						Yes	Yes	Yes	Can't tell	Yes
43	29934260	Multicente	Sato Y, Nishiofuku H, Y	Yes	Yes						Yes	Yes	Yes	Can't tell	Yes
44	29719302	A Randomi	Thomas MB, Garrett-M	Yes	Yes	Yes	Yes	Yes	Yes	Yes					
45	29631810	Sorafenib p	Kudo M, Ueshima K, Yo	Yes	Yes	Yes	Yes	Yes	Can't tell	Yes					
46	29563636	A multicen	Palmer DH, Ma YT, Pe	Yes	Yes	Yes	Yes	Yes	Can't tell	Yes					
47	29552783	Sunitinib v	Xu Q, Huang Y, Shi H, S	Yes	Yes	Can't tell	Yes	Yes	Can't tell	Yes					
48	29520435	A phase I t	El-Khoueiry AB, O'Don	Yes	Yes						Yes	Yes	Can't tell	Can't tell	No
49	29498924	SIRveNIB: S	Chow PKH, Gandhi M,	Yes	Yes	Yes	Yes	Yes	Yes	Yes					
50	29474553	A multicen	Suzuki E, Kaneko S, Ok	Yes	Yes						Yes	Yes	No	Can't tell	No
51	29433850	Lenvatinib	Kudo M, Finn RS, Qin S	Yes	Yes	Yes	Yes	Yes	Can't tell	Yes					
52	29209114	Combined	Zhang ZH, Liu QX, Zhar	Yes	Yes						Can't tell	Can't tell	Yes	No	Can't tell
53	29107679	Efficacy an	Vilgrain V, Pereira H, A	Yes	Yes	Yes	Yes	Yes	Can't tell	Yes					
54	28648803	Sorafenib i	Meyer T, Fox R, Ma YT	Yes	Yes	Yes	Yes	Yes	Yes	Yes					
55	28592620	Phase II St	Abou-Alfa GK, Blanc JF	Yes	Yes						No	Yes	Yes	Can't tell	Yes
56	28573606	A Phase I S	Sho T, Nakanishi M, M	Yes	Yes						No	Yes	Can't tell	Can't tell	Yes
57	28465443	Phase I and	Duffy AG, Ma C, Ulaha	Yes	Yes						Yes	Yes	Yes	Can't tell	Yes
58	28272913	Phase I Stu	Ishizaki M, Kaibori M,	Yes	Yes						Yes	Yes	Yes	Can't tell	Yes
59	28120036	Phase Ib st	Abou-Alfa GK, Yen CJ,	Yes	Yes						Yes	Yes	Yes	Can't tell	Yes
60	27943153	Phase I/II F	Hubbard JM, Mahoney	Yes	Yes						Yes	Yes	Yes	Can't tell	Yes
61	27909950	HATT: a ph	Lin SM, Lu SN, Chen PT	Yes	Yes						Yes	Yes	Yes	Can't tell	No
62	27793949	Sorafenib (	Giorgio A, Merola MG,	Yes	Yes	Yes	Yes	Yes	Can't tell	Yes					
63	27573564	Sorafenib p	Ikeda M, Shimizu S, Sa	Yes	Yes	Yes	Yes	Yes	Can't tell	no					
64	27755109	Real-life e:	Merchante N, Ibarra S,	Yes	Yes						Yes	Yes	Yes	Can't tell	Yes
65	27681866	A phase Ib	Tai WM, Yong WP, Lim	Yes	Yes						Yes	Yes	Yes	Can't tell	Yes
66	27256874	Phase I Stu	Shahda S, Loehrer PJ, C	Yes	Yes						Can't tell	Yes	Yes	Can't tell	Yes
67	22033636	Phase II tri	Petrini I, Lencioni M, R	Yes	Yes						Yes	Yes	Yes	Can't tell	Yes
68	21941188	A single ce	Song T, Zhang W, Wu	Yes	Yes						Yes	Yes	Yes	Can't tell	Yes
69	21932373	Efficacy an	Gomez-Martin C, Bust	Yes	Yes						Yes	Yes	Yes	Can't tell	Yes
70	21911714	Phase II tri	Pawlik TM, Reyes DK, C	Yes	Yes						Yes	Yes	Yes	Can't tell	Yes
71	21695438	Phase 1 tri	Lee SJ, Lee J, Park SH,	Yes	Yes						Yes	Yes	Yes	Can't tell	Yes
72	21664811	Phase III st	Kudo M, Imanaka K, Ch	Yes	Yes	Can't tell	Yes	Yes	Can't tell	Yes					
73	21340026	Reversible	Coriat R, Gouya H, Mir	Yes	Yes						Yes	Yes	Yes	Can't tell	Yes
74	21081728	Doxorubici	Abou-Alfa GK, Johnsor	Yes	Yes	Yes	Yes	Yes	Yes	Yes					
75	21036880	Continuous	Dufour JF, Hoppe H, He	Yes	Yes						Yes	Yes	Yes	Can't tell	Yes
76	20416968	Phase II stu	Hsu CH, Shen YC, Lin Z	Yes	Yes						Yes	Yes	Yes	Can't tell	Yes
77	20041325	Sorafenib p	Prete SD, Montella L, C	Yes	Yes						Yes	Yes	Yes	Can't tell	Yes
78	24312711	Practical e	Jeong SW, Jang JY, Shi	Yes	Yes						No	Yes	Yes	Can't tell	Can't tell
79	24081937	Sunitinib v	Cheng AL, Kang YK, Lin	Yes	Yes	Yes	Yes	Yes	No	Yes					
80	23980084	Brivanib ve	Johnson PJ, Qin S, Park	Yes	Yes	Yes	Yes	Yes	Yes	No					

81	Phase I study inve	Finn RS, Poon RT, Yau T, Klümpen		Yes	Yes						Can't tell	Yes	Can't tell	Can't tell	Can't tell
82	23824645	Sorafenib \	Abdel-Rahman O, Abd	Yes	Yes						Can't tell	Yes	No	Can't tell	No
83	23749944	Phase I adj	Jia N, Liou I, Halldorso	Yes	Yes						No	Yes	No	No	Can't tell
84	23519998	Temsirolin	Kelley RK, Nimeiri HS,	Yes	Yes						Yes	Yes	Yes	Can't tell	Yes
85	23431262	Sorafenib i	Køstner AH, Sorensen	Yes	Yes						Yes	Can't tell	Yes	No	No
86	23324079	Sorafenib i	Bai W, Wang YJ, Zhao	Yes	Yes						Yes	Yes	Yes	Can't tell	No
87	23263829	Sorafenib i	Brunocilla PR, Brunello	Yes	Yes						Can't tell	Yes	Yes	Can't tell	No
88	23041587	Sorafenib i	Pressiani T, Boni C, Rir	Yes	Yes						Yes	Yes	Yes	No	No
89	22524575	The efficac	Jeong SW, Jang JY, Lee	Yes	Yes						Can't tell	Yes	Yes	Yes	Yes
90	22334456	Transarteri	Sansonno D, Lauletta (	Yes	Yes	Yes	Yes	Yes	Yes	Yes					
91	22314421	Phase II stu	Park JW, Koh YH, Kim I	Yes	Yes						Yes	Yes	Yes	Can't tell	Yes
92	22215073	Conventior	Sieghart W, Pinter M,	Yes	Yes						Yes	Yes	Yes	Yes	Yes
93	26952006	Resminos	Bitzer M, Horger M, (	Yes	Yes		Yes	Yes			Yes	Yes	Yes	Can't tell	Yes
94	26884590	Sorafenib	Koeberle D, Dufour .	Yes	Yes	Yes	Yes	Yes	Yes	Yes					
95	26867886	Phase 1 Tri	Brade AM, Ng S, Brierl	Yes	Yes						Yes	Yes	Yes	Can't tell	Can't tell
96	26809111	Sorafenib (	Lencioni R, Llovet JM,	Yes	Yes	Yes	Yes	Yes	Yes	Yes					
97	26802147	A randomi:	Ciuleanu T, Bazin I, Lur	Yes	Yes	Yes	Yes	Yes	Yes	Yes					
98	26446238	Sorafenib \	Zhang Y, Fan W, Wang	Yes	Yes						Yes	Yes	Yes	Can't tell	Yes*
99	26361969	Adjuvant s	Bruix J, Takayama T, M	Yes	Yes	Yes	Yes	Yes	Yes	Yes					
100	26071796	Safety and	Cheng AL, Kang YK, He	Yes	Yes						Yes	Yes	Yes	Yes	Yes
101	25957784	Impact of i	Hoffmann K, Ganten T	Yes	Yes	Yes	Yes	Yes	Yes	Can't tell					
102	26069923	Open-Labe	Cosgrove DP, Reyes DK	Yes	Yes						Yes	Yes	Yes	Can't tell	Yes
103	26989044	Concurrent	Yao X, Yan D, Zeng H, I	Yes	Yes						Yes	Yes	Yes	Can't tell	Yes
104	27082062	Randomize	Cheng AL, Thongprasei	Yes	Yes	Yes	Yes	Yes	No	Yes					
105	26644411	A Phase I S	Adjei AA, Richards DA,	Yes	Yes						Yes	Yes	Yes	Can't tell	Yes
106	25683938	Sorafenib (	Kan X, Jing Y, Wan QY,	Yes	Yes	Yes	Yes	Yes	Can't tell	Can't tell					
107	25583146	[Therapeut	Turnes J, Díaz R, Herna	Yes	Yes						Yes	Yes	Yes	Can't tell	Yes
108	25547503	SEARCH: a	Zhu AX, Rosmorduc O,	Yes	Yes	Yes	Yes	Yes	Yes	Yes					
109	25488963	Linifanib v	Cainap C, Qin S, Huang	Yes	Yes	Yes	Yes	Yes	No	Can't tell					
110	25294897	A phase II :	Lim HY, Heo J, Choi HJ,	Yes	Yes						Yes	Yes	Yes	Can't tell	Yes
111	25294187	Phase 1 tri	Puzanov I, Sosman J, S	Yes	Yes						Yes	Yes	Yes	No	Yes
112	25173458	TACE plus :	Erhardt A, Kolligs F, Dc	Yes	Yes						Yes	Yes	Yes	Can't tell	Yes
113	25099027	The combi	Chao Y, Chung YH, Har	Yes	Yes						Yes	Yes	Yes	Can't tell	Yes
114	24977690	Randomize	Lee FA, Zee BC, Cheun	Yes	Yes	Yes	Can't tell	Yes	Can't tell	Can't tell					
115	24930619	Safety and	Ricke J, Bulla K, Kolligs	Yes	Yes	Yes	Yes	Yes	Can't tell	Can't tell					
116	24894839	Analysis of	Zheng J, Shao G, Luo J.	Yes	Yes						Yes	Yes	Yes	Can't tell	Yes
117	24810940	Efficacy an	Srimuninnimit V, Sriur	Yes	Yes						Yes	Yes	Yes	Can't tell	Yes
118	24793745	Neutrophil	Wei K, Wang M, Zhang	Yes	Yes						Yes	Yes	Yes	Can't tell	Yes
119	24740650	Safety and efficacy of sorafenib ir		Yes	Yes						Yes	Yes	Yes	Can't tell	Yes
120	24698672	Sorafenib i	Ji YX, Zhang ZF, Lan KT	Yes	Yes	Yes	Yes	Yes	Can't tell	Can't tell					
121	24681342	Prospective	Kulik L, Vouche M, Kop	Yes	Yes	Yes	Yes	Yes	Can't tell	No					
122	24661657	Phase 2 study of combined sorafe		Yes	Yes						Yes	Yes	Yes	No	Yes
123	24614178	Multicenter phase II study of sequ		Yes	Yes						Yes	Yes	Yes	Can't tell	Yes
124	24599799	A phase I/II study of S-1 with sora		Yes	Yes						Yes	Yes	Yes	Can't tell	Yes
125	24438504	Phase I study of combination cher		Yes	Yes						Yes	Yes	Yes	Can't tell	No
126	24350564	The feasibility of combined transc		Yes	Yes						Yes	Yes	Yes	Can't tell	Yes
127	24333135	Efficacy and safety of sorafenib-g		Yes	Yes						Yes	Yes	Yes	Can't tell	No
128	32206991	Metabolic	Castello A, Rimassa L,	Yes	Yes						Yes	Yes	Yes	Can't tell	Yes
129	32084526	Efficacy ar	Kim BK, Kim DY, Byun	Yes	Yes						Yes	Yes	Yes	Can't tell	Yes
130	31801872	Randomise	Kudo M, Ueshima K, Ik	Yes	Yes	Yes	Yes	Yes	No	Yes					
131	31615466	Randomize	Kondo M, Morimoto M	Yes	Yes	Yes	Yes	Yes	Can't tell	No					
132	31615466	Randomize	Kondo M, Morimoto M	Yes	Yes	Yes	Yes	Yes	Can't tell	No					
133	31429027	Efficacy ar	Mokdad AA, Zhu H, Be	Yes	Yes						Yes	Yes	Yes	Can't tell	no
134	31305287	Phase I Stu	Gordon SW, McGuire \	Yes	Yes						Yes	Yes	Yes	Can't tell	No
135	31295152	A Phase 2	Kelley RK, Gane E, Ass	Yes	Yes						Yes	Yes	Yes	Can't tell	No
136	31185950	A prospect	Eilard MS, Andersson	Yes	Yes						Yes	Yes	Yes	Can't tell	Yes
137	31125576	Pravastatir	Jouve JL, Lecomte T, B	Yes	Yes	Yes	Yes	Yes	No	Yes					
138	31125576	Pravastatir	Jouve JL, Lecomte T, B	Yes	Yes	Yes	Yes	Yes	No	Yes					
