# Supplementary material for: Prevalence of gastrointestinal side effects in hepatocellular carcinoma patients receiving sorafenib: a meta-analysis of 136 studies and 14,416 patients
Source: Ther Adv Med Oncol. 2026 May 13;18:17588359261442686. doi: 10.1177/17588359261442686 (PMC13180223; doi:10.1177/17588359261442686)
Supplement: sj-pdf-4-tam-10.1177_17588359261442686 – Supplemental material for Prevalence of gastrointestinal side effects in hepatocellular carcinoma patients receiving sorafenib: a meta-analysis of 136 studies and 14,416 patients [file sj-pdf-4-tam-10.1177_17588359261442686.pdf]

| PMID | Title | Citation | First Author |
|---|---|---|---|
| 39673613 | Absorption, metabolism, and excretion of oral [(14)C] radiolabeled donafenib: an open-label, phase I, single-dose study in humans | Cancer Chemother Pharmacol. 2024 Dec 14;95(1):5. doi: 10.1007/s00280-024-04725-w. | Ma S |
| 39412677 | Association of adverse events and quality of life in patients with unresectable hepatocellular carcinoma | Qual Life Res. 2024 Dec;33(12):3377-3386. doi: 10.1007/s11136-024-03779-w. Epub 2024 Oct 16. | Agirrezabal I |
| 39141577 | Efficacy and safety of bintrafusp alfa in 2 phase I expansion cohorts with advanced HCC | Hepatology. 2025 Jan 1;81(1):32-43. doi: 10.1097/HEP.0000000000001054. Epub 2024 Aug 13. | Lim HY |
| 39089633 | Outcomes in the Asian subgroup of the phase III randomised HIMALAYA study of tremelimumab plus durvalumab in unresectable hepatocellular carcinoma | J Hepatol. 2025 Feb;82(2):258-267. doi: 10.1016/j.jhep.2024.07.017. Epub 2024 Jul 31. | Lau G |
| 39007783 | Skeletal muscle quality predicts overall survival in advanced liver hepatocellular carcinoma treated with SIRT and sorafenib: A subanalysis of the SORAMIC trial | United European Gastroenterol J. 2024 Oct;12(8):1016-1027. doi: 10.1002/ueg2.12627. Epub 2024 Jul 15. | Surov A |
| 38900435 | Survival in Patients With Recurrent Intermediate-Stage Hepatocellular Carcinoma: Sorafenib Plus TACE vs TACE Alone Randomized Clinical Trial | JAMA Oncol. 2024 Aug 1;10(8):1047-1054. doi: 10.1001/jamaoncol.2024.1831. | Fan W |
| 38844309 | Nivolumab plus ipilimumab combination therapy in patients with advanced hepatocellular carcinoma previously treated with sorafenib: 5-year results from CheckMate 040 | Ann Oncol. 2024 Jun;35(6):537-548. doi: 10.1016/j.annonc.2024.03.005. Epub 2024 May 22. | Melero I |
| 38805668 | Patient-Reported Outcomes From the Phase III HIMALAYA Study of Tremelimumab Plus Durvalumab in Unresectable Hepatocellular Carcinoma | J Clin Oncol. 2024 Aug 10;42(23):2790-2799. doi: 10.1200/JCO.23.01462. Epub 2024 May 28. | Sangro B |
| 38568599 | Adjuvant Transarterial Chemoembolization With Sorafenib for Portal Vein Tumor Thrombus: A Randomized Clinical Trial | JAMA Surg. 2024 Jun 1;159(6):616-624. doi: 10.1001/jamasurg.2024.0506. | Peng Z |
| 38437209 | Cost-effectiveness analysis of Tislelizumab vs Sorafenib as the first-line treatment of unresectable hepatocellular carcinoma | PLoS One. 2024 Mar 4;19(3):e0295090. doi: 10.1371/journal.pone.0295090. eCollection 2024. | Chen Q |
| 38382875 | Four-year overall survival update from the phase III HIMALAYA study of tremelimumab plus durvalumab in unresectable hepatocellular carcinoma | Ann Oncol. 2024 May;35(5):448-457. doi: 10.1016/j.annonc.2024.02.005. Epub 2024 Feb 19. | Sangro B |
| 38364832 | Cabozantinib plus atezolizumab versus sorafenib for advanced hepatocellular carcinoma (COSMIC-312): final results of a randomised phase 3 study | Lancet Gastroenterol Hepatol. 2024 Apr;9(4):310-322. doi: 10.1016/S2468-1253(23)00454-5. Epub 2024 Feb 13. | Yau T |
| 38363156 | CALGB 80802 (Alliance): Impact of Sorafenib with and without Doxorubicin on Hepatitis C Infection in Patients with Advanced Hepatocellular Carcinoma | Cancer Res Commun. 2024 Mar 7;4(3):682-690. doi: 10.1158/2767-9764.CRC-22-0516. | Abou-Alfa GK |
| 38300386 | Cost-Effectiveness Analysis of Camrelizumab Plus Rivoceranib Versus Sorafenib as a First-Line Therapy for Unresectable Hepatocellular Carcinoma in the Chinese Health Care System | Clin Drug Investig. 2024 Mar;44(3):149-162. doi: 10.1007/s40261-024-01343-5. Epub 2024 Feb 1. | Lang W |
| 38270822 | Phase 1 trial of navitoclax and sorafenib in patients with relapsed or refractory solid tumors with hepatocellular carcinoma expansion cohort | Invest New Drugs. 2024 Feb;42(1):127-135. doi: 10.1007/s10637-024-01420-8. Epub 2024 Jan 25. | Emiloju OE |
| 38151184 | Nivolumab in sorafenib-naive and sorafenib-experienced patients with advanced hepatocellular carcinoma: 5-year follow-up from CheckMate 040 | Ann Oncol. 2024 Apr;35(4):381-391. doi: 10.1016/j.annonc.2023.12.008. Epub 2023 Dec 25. | El-Khoueiry AB |
| 37796513 | Tislelizumab vs Sorafenib as First-Line Treatment for Unresectable Hepatocellular Carcinoma: A Phase 3 Randomized Clinical Trial | JAMA Oncol. 2023 Dec 1;9(12):1651-1659. doi: 10.1001/jamaoncol.2023.4003. | Qin S |
| 37676259 | FDA Approval Summary: Tremelimumab in Combination with Durvalumab for the Treatment of Patients with Unresectable Hepatocellular Carcinoma | Clin Cancer Res. 2024 Jan 17;30(2):269-273. doi: 10.1158/1078-0432.CCR-23-2124. | Patel TH |
| 37499670 | Camrelizumab plus rivoceranib versus sorafenib as first-line therapy for unresectable hepatocellular carcinoma (CARES-310): a randomised, open-label, international phase 3 study | Lancet. 2023 Sep 30;402(10408):1133-1146. doi: 10.1016/S0140-6736(23)00961-3. Epub 2023 Jul 24. | Qin S |
| 37219875 | Impact of body composition in advanced hepatocellular carcinoma: A subanalysis of the SORAMIC trial | Hepatol Commun. 2023 May 23;7(6):e0165. doi: 10.1097/HC9.0000000000000165. eCollection 2023 Jun 1. | Surov A |
| 37188895 | A Phase I Study to Investigate the Safety, Tolerability and Pharmacokinetics of Napabucasin Combined with Sorafenib in Japanese Patients with Unresectable Hepatocellular Carcinoma | Drugs R D. 2023 Jun;23(2):99-107. doi: 10.1007/s40268-023-00416-8. Epub 2023 May 15. | Okusaka T |
| 37165980 | Exposure-response analysis for nivolumab plus ipilimumab combination therapy in patients with advanced hepatocellular carcinoma (CheckMate 040) | Clin Transl Sci. 2023 Aug;16(8):1445-1457. doi: 10.1111/cts.13544. Epub 2023 May 30. | Sangro B |
| 37038986 | Irradiation stent with 125 I plus TACE versus sorafenib plus TACE for hepatocellular carcinoma with major portal vein tumor thrombosis: a multicenter randomized trial | Int J Surg. 2023 May 1;109(5):1188-1198. doi: 10.1097/JS9.0000000000000295. | Lu J |
| 36512738 | Nivolumab Plus Cabozantinib With or Without Ipilimumab for Advanced Hepatocellular Carcinoma: Results From Cohort 6 of the CheckMate 040 Trial | J Clin Oncol. 2023 Mar 20;41(9):1747-1757. doi: 10.1200/JCO.22.00972. Epub 2022 Dec 13. | Yau T |
| 36455168 | Pembrolizumab Versus Placebo as Second-Line Therapy in Patients From Asia With Advanced Hepatocellular Carcinoma: A Randomized, Double-Blind, Phase III Trial | J Clin Oncol. 2023 Mar 1;41(7):1434-1443. doi: 10.1200/JCO.22.00620. Epub 2022 Dec 1. | Qin S |
| 36341767 | Overall survival and objective response in advanced unresectable hepatocellular carcinoma: A subanalysis of the REFLECT study | J Hepatol. 2023 Jan;78(1):133-141. doi: 10.1016/j.jhep.2022.09.006. Epub 2022 Sep 20. | Kudo M |
| 38319892 | Tremelimumab plus Durvalumab in Unresectable Hepatocellular Carcinoma | NEJM Evid. 2022 Aug;1(8):EVIDoa2100070. doi: 10.1056/EVIDoa2100070. Epub 2022 Jun 6. | Abou-Alfa GK |
| 35881180 | Non-hypervascular hepatobiliary phase hypointense lesions detected in patients with hepatocellular carcinoma: a post hoc analysis of SORAMIC trial to identify risk factors for progression | Eur Radiol. 2023 Jan;33(1):493-500. doi: 10.1007/s00330-022-09000-1. Epub 2022 Jul 26. | Öcal O |
| 35798016 | Cabozantinib plus atezolizumab versus sorafenib for advanced hepatocellular carcinoma (COSMIC-312): a multicentre, open-label, randomised, phase 3 trial | Lancet Oncol. 2022 Aug;23(8):995-1008. doi: 10.1016/S1470-2045(22)00326-6. Epub 2022 Jul 4. | Kelley RK |
| 35763041 | Extracellular Vesicles May Predict Response to Radioembolization and Sorafenib Treatment in Advanced Hepatocellular Carcinoma: An Exploratory Analysis from the SORAMIC Trial | Clin Cancer Res. 2022 Sep 1;28(17):3890-3901. doi: 10.1158/1078-0432.CCR-22-0569. | Shuen TWH |
| 35487183 | Quality of life assessment of cabozantinib in patients with advanced hepatocellular carcinoma in the CELESTIAL trial | Eur J Cancer. 2022 Jun;168:91-98. doi: 10.1016/j.ejca.2022.03.021. Epub 2022 Apr 26. | Freemantle N |
| 35421228 | Pembrolizumab Monotherapy for Previously Untreated Advanced Hepatocellular Carcinoma: Data from the Open-Label, Phase II KEYNOTE-224 Trial | Clin Cancer Res. 2022 Jun 13;28(12):2547-2554. doi: 10.1158/1078-0432.CCR-21-3807. | Verset G |
| 35364421 | Updated efficacy and safety of KEYNOTE-224: a phase II study of pembrolizumab in patients with advanced hepatocellular carcinoma previously treated with sorafenib | Eur J Cancer. 2022 May;167:1-12. doi: 10.1016/j.ejca.2022.02.009. Epub 2022 Mar 29. | Kudo M |
| 35337274 | Comparison of the outcomes between sorafenib and lenvatinib as the first-line systemic treatment for HBV-associated hepatocellular carcinoma: a propensity score matching analysis | BMC Gastroenterol. 2022 Mar 25;22(1):135. doi: 10.1186/s12876-022-02210-3. | Choi NR |
| 35274724 | Phase I Study Evaluating Dose De-escalation of Sorafenib with Metformin and Atorvastatin in Hepatocellular Carcinoma (SMASH) | Oncologist. 2022 Mar 11;27(3):165-e222. doi: 10.1093/oncolo/oyab008. | Ostwal V |
| 35226112 | A clinical scoring tool validated with machine learning for predicting severe hand-foot syndrome from sorafenib in hepatocellular carcinoma | Cancer Chemother Pharmacol. 2022 Apr;89(4):479-485. doi: 10.1007/s00280-022-04411-9. Epub 2022 Feb 28. | Abuhelwa AY |
| 35103539 | Sorafenib Plus Hepatic Arterial Infusion Chemotherapy versus Sorafenib for Hepatocellular Carcinoma with Major Portal Vein Tumor Thrombosis: A Randomized Trial | Radiology. 2022 May;303(2):455-464. doi: 10.1148/radiol.211545. Epub 2022 Feb 1. | Zheng K |
| 34986867 | Hepatocellular carcinoma patients with high circulating cytotoxic T cells and intra-tumoral immune signature benefit from pembrolizumab: results from a single-arm phase 2 trial | Genome Med. 2022 Jan 6;14(1):1. doi: 10.1186/s13073-021-00995-8. | Hong JY |
| 34982369 | GALNT14 genotype-guided chemoembolization plus sorafenib therapy in hepatocellular carcinoma: a randomized trial | Hepatol Int. 2022 Feb;16(1):148-158. doi: 10.1007/s12072-021-10283-7. Epub 2022 Jan 4. | Chen WT |
| 34923912 | The transcatheter arterial chemoembolization combined with targeted nanoparticle delivering sorafenib system for the treatment of microvascular invasion of hepatocellular carcinoma | Bioengineered. 2021 Dec;12(2):11124-11135. doi: 10.1080/21655979.2021.2001239. | Su D |
| 34914889 | Nivolumab versus sorafenib in advanced hepatocellular carcinoma (CheckMate 459): a randomised, multicentre, open-label, phase 3 trial | Lancet Oncol. 2022 Jan;23(1):77-90. doi: 10.1016/S1470-2045(21)00604-5. Epub 2021 Dec 13. | Yau T |
| 34910380 | Validation of the prophylactic efficacy of urea-based creams on sorafenib-induced hand-foot skin reaction in patients with advanced hepatocellular carcinoma: A randomised experiment study | Cancer Rep (Hoboken). 2022 Jul;5(7):e1532. doi: 10.1002/cnr2.1532. Epub 2021 Dec 14. | Lien RY |
| 34905388 | Arterial Chemotherapy of Oxaliplatin Plus Fluorouracil Versus Sorafenib in Advanced Hepatocellular Carcinoma: A Biomolecular Exploratory, Randomized, Phase III Trial (FOHAIC-1) | J Clin Oncol. 2022 Feb 10;40(5):468-480. doi: 10.1200/JCO.21.01963. Epub 2021 Dec 14. | Lyu N |
| 34902530 | Updated efficacy and safety data from IMbrave150: Atezolizumab plus bevacizumab vs. sorafenib for unresectable hepatocellular carcinoma | J Hepatol. 2022 Apr;76(4):862-873. doi: 10.1016/j.jhep.2021.11.030. Epub 2021 Dec 11. | Cheng AL |
| 34735674 | A phase 1 study of pegylated recombinant arginase (PEG-BCT-100) in combination with systemic chemotherapy (capecitabine and oxaliplatin)[PACOX] in advanced hepatocellular carcinoma patients | Invest New Drugs. 2022 Apr;40(2):314-321. doi: 10.1007/s10637-021-01178-3. Epub 2021 Nov 4. | Yau T |
| 34686780 | Prognostic value of baseline imaging and clinical features in patients with advanced hepatocellular carcinoma | Br J Cancer. 2022 Feb;126(2):211-218. doi: 10.1038/s41416-021-01577-6. Epub 2021 Oct 22. | Öcal O |
| 34621044 | Efficacy and safety of cabozantinib for patients with advanced hepatocellular carcinoma based on albumin-bilirubin grade | Br J Cancer. 2022 Mar;126(4):569-575. doi: 10.1038/s41416-021-01532-5. Epub 2021 Oct 7. | Kelley RK |
| 34614432 | Efficacy comparison of optimal treatments for hepatocellular carcinoma patients with portal vein tumor thrombus | Ann Hepatol. 2022 Jan-Feb;27(1):100552. doi: 10.1016/j.aohep.2021.100552. Epub 2021 Oct 3. | Zhang Y |
| 34541612 | Gadoxetic acid uptake as a molecular imaging biomarker for sorafenib resistance in patients with hepatocellular carcinoma: a post hoc analysis of the SORAMIC trial | J Cancer Res Clin Oncol. 2022 Sep;148(9):2487-2496. doi: 10.1007/s00432-021-03803-3. Epub 2021 Sep 20. | Öcal O |
| 34454995 | Liver function after combined selective internal radiation therapy or sorafenib monotherapy in advanced hepatocellular carcinoma | J Hepatol. 2021 Dec;75(6):1387-1396. doi: 10.1016/j.jhep.2021.07.037. Epub 2021 Aug 27. | Ricke J |
| 34407972 | Upregulation of C/EBPα Inhibits Suppressive Activity of Myeloid Cells and Potentiates Antitumor Response in Mice and Patients with Cancer | Clin Cancer Res. 2021 Nov 1;27(21):5961-5978. doi: 10.1158/1078-0432.CCR-21-0986. Epub 2021 Aug 18. | Hashimoto A |
| 34351608 | Efficacy and Safety Results from a Phase 2, Randomized, Double-Blind Study of Enzalutamide Versus Placebo in Advanced Hepatocellular Carcinoma | Clin Drug Investig. 2021 Sep;41(9):795-808. doi: 10.1007/s40261-021-01063-0. Epub 2021 Aug 5. | Ryoo BY |
| 34297268 | Priming of Sorafenib Prior to Radiofrequency Ablation Does Not Increase Treatment Effect in Hepatocellular Carcinoma | Dig Dis Sci. 2022 Jul;67(7):3455-3463. doi: 10.1007/s10620-021-07156-2. Epub 2021 Jul 23. | Bockorny B |
| 34292792 | Safety, Efficacy, and Pharmacodynamics of Tremelimumab Plus Durvalumab for Patients With Unresectable Hepatocellular Carcinoma: Randomized Expansion of a Phase I/II Study | J Clin Oncol. 2021 Sep 20;39(27):2991-3001. doi: 10.1200/JCO.20.03555. Epub 2021 Jul 22. | Kelley RK |
| 34243077 | Health-related quality of life in locally advanced hepatocellular carcinoma treated by either radioembolisation or sorafenib (SARAH trial) | Eur J Cancer. 2021 Sep;154:46-56. doi: 10.1016/j.ejca.2021.05.032. Epub 2021 Jul 6. | Pereira H |
| 34237154 | Transarterial chemoembolization plus lenvatinib versus transarterial chemoembolization plus sorafenib as first-line treatment for hepatocellular carcinoma with portal vein tumor thrombus: A prospective randomized study | Cancer. 2021 Oct 15;127(20):3782-3793. doi: 10.1002/cncr.33677. Epub 2021 Jul 8. | Ding X |
| 34236269 | The Impact of Sorafenib in Combination with Intermittent Hepatic Arterial Infusion Chemotherapy for Unresectable Hepatocellular Carcinoma with Major Vascular Invasion | Cancer Invest. 2022 Jan;40(1):81-89. doi: 10.1080/07357907.2021.1952594. Epub 2021 Aug 26. | Kaibori M |
| 34189869 | Characterization of response to atezolizumab + bevacizumab versus sorafenib for hepatocellular carcinoma: Results from the IMbrave150 trial | Cancer Med. 2021 Aug;10(16):5437-5447. doi: 10.1002/cam4.4090. Epub 2021 Jun 29. | Salem R |
| 34185551 | Donafenib Versus Sorafenib in First-Line Treatment of Unresectable or Metastatic Hepatocellular Carcinoma: A Randomized, Open-Label, Parallel-Controlled Phase II-III Trial | J Clin Oncol. 2021 Sep 20;39(27):3002-3011. doi: 10.1200/JCO.21.00163. Epub 2021 Jun 29. | Qin S |
| 34143971 | Sintilimab plus a bevacizumab biosimilar (IBI305) versus sorafenib in unresectable hepatocellular carcinoma (ORIENT-32): a randomised, open-label, phase 2-3 study | Lancet Oncol. 2021 Jul;22(7):977-990. doi: 10.1016/S1470-2045(21)00252-7. Epub 2021 Jun 15. | Ren Z |
| 34139320 | Percutaneous ablation for locally advanced hepatocellular carcinoma with tumor portal invasion | Clin Res Hepatol Gastroenterol. 2021 Nov;45(6):101731. doi: 10.1016/j.clinre.2021.101731. Epub 2021 Jun 15. | Blaise L |
| 34108184 | Pharmacodynamic Biomarkers Predictive of Survival Benefit with Lenvatinib in Unresectable Hepatocellular Carcinoma: From the Phase III REFLECT Study | Clin Cancer Res. 2021 Sep 1;27(17):4848-4858. doi: 10.1158/1078-0432.CCR-20-4219. Epub 2021 Jun 9. | Finn RS |
| 34087115 | Lenvatinib versus sorafenib for first-line treatment of unresectable hepatocellular carcinoma: patient-reported outcomes from a randomised, open-label, non-inferiority, phase 3 trial | Lancet Gastroenterol Hepatol. 2021 Aug;6(8):649-658. doi: 10.1016/S2468-1253(21)00110-2. Epub 2021 Jun 2. | Vogel A |
| 34059326 | Cost-Utility Analysis of Transarterial Radioembolization With Yttrium-90 Resin Microspheres Compared With Sorafenib in Locally Advanced and Inoperable Hepatocellular Carcinoma | Clin Ther. 2021 Jul;43(7):1201-1212. doi: 10.1016/j.clinthera.2021.04.018. Epub 2021 May 28. | Zarca K |
| 34051880 | Patient-reported outcomes with atezolizumab plus bevacizumab versus sorafenib in patients with unresectable hepatocellular carcinoma (IMbrave150): an open-label, randomised, phase 3 trial | Lancet Oncol. 2021 Jul;22(7):991-1001. doi: 10.1016/S1470-2045(21)00151-0. Epub 2021 May 27. | Galle PR |
| 33972742 | Randomised Phase 1b/2 trial of tepotinib vs sorafenib in Asian patients with advanced hepatocellular carcinoma with MET overexpression | Br J Cancer. 2021 Jul;125(2):200-208. doi: 10.1038/s41416-021-01380-3. Epub 2021 May 10. | Ryoo BY |
| 33971141 | Apatinib as second-line or later therapy in patients with advanced hepatocellular carcinoma (AHELP): a multicentre, double-blind, randomised, placebo-controlled, phase 3 trial | Lancet Gastroenterol Hepatol. 2021 Jul;6(7):559-568. doi: 10.1016/S2468-1253(21)00109-6. Epub 2021 May 8. | Qin S |
| 33856599 | A phase II clinical study on the efficacy and predictive biomarker of pegylated recombinant arginase on hepatocellular carcinoma | Invest New Drugs. 2021 Oct;39(5):1375-1382. doi: 10.1007/s10637-021-01111-8. Epub 2021 Apr 15. | Chan SL |
| 33855585 | Baseline Interleukin-6 and -8 predict response and survival in patients with advanced hepatocellular carcinoma treated with sorafenib monotherapy: an exploratory post hoc analysis of the SORAMIC trial | J Cancer Res Clin Oncol. 2022 Feb;148(2):475-485. doi: 10.1007/s00432-021-03627-1. Epub 2021 Apr 14. | Öcal O |
| 33824476 | Phase 1b/2 trial of tepotinib in sorafenib pretreated advanced hepatocellular carcinoma with MET overexpression | Br J Cancer. 2021 Jul;125(2):190-199. doi: 10.1038/s41416-021-01334-9. Epub 2021 Apr 6. | Decaens T |
| 33813432 | Efficacy of Ramucirumab Versus Sorafenib as Subsequent Treatment for Hepatocellular Carcinoma | Anticancer Res. 2021 Apr;41(4):2187-2192. doi: 10.21873/anticanres.14993. | Maesaka K |
| 33655688 | Potential of novel colchicine dosage schedule for the palliative treatment of advanced hepatocellular carcinoma | Kaohsiung J Med Sci. 2021 Jul;37(7):616-623. doi: 10.1002/kjm2.12374. Epub 2021 Mar 3. | Lin ZY |
| 33646489 | Phase IB study of sorafenib and evofosfamide in patients with advanced hepatocellular and renal cell carcinomas (NCCTG N1135, Alliance) | Invest New Drugs. 2021 Aug;39(4):1072-1080. doi: 10.1007/s10637-021-01090-w. Epub 2021 Mar 1. | Tran NH |
| 33556230 | Atezolizumab and bevacizumab combination compared with sorafenib as the first-line systemic treatment for patients with unresectable hepatocellular carcinoma: A cost-effectiveness analysis in China and the United states | Liver Int. 2021 May;41(5):1097-1104. doi: 10.1111/liv.14795. Epub 2021 Feb 8. | Wen F |
| 33481328 | Efficacy and safety of sorafenib plus vitamin K treatment for hepatocellular carcinoma: A phase II, randomized study | Cancer Med. 2021 Feb;10(3):914-922. doi: 10.1002/cam4.3674. Epub 2021 Jan 22. | Haruna Y |
| 33472666 | Axitinib in combination with radiotherapy for advanced hepatocellular carcinoma: a phase I clinical trial | Radiat Oncol. 2021 Jan 20;16(1):18. doi: 10.1186/s13014-020-01742-w. | Yang KL |
| 33464318 | Cost-effectiveness of Pembrolizumab as a Second-Line Therapy for Hepatocellular Carcinoma | JAMA Netw Open. 2021 Jan 4;4(1):e2033761. doi: 10.1001/jamanetworkopen.2020.33761. | Chiang CL |
| 33420951 | Phase 2 trial comparing sorafenib, pravastatin, their combination or supportive care in HCC with Child-Pugh B cirrhosis | Hepatol Int. 2021 Feb;15(1):93-104. doi: 10.1007/s12072-020-10120-3. Epub 2021 Jan 9. | Blanc JF |
| 33231873 | Health-related quality-of-life impact of pembrolizumab versus best supportive care in previously systemically treated patients with advanced hepatocellular carcinoma: KEYNOTE-240 | Cancer. 2021 Mar 15;127(6):865-874. doi: 10.1002/cncr.33317. Epub 2020 Nov 24. | Ryoo BY |
| 33197225 | Systemic Therapy for Advanced Hepatocellular Carcinoma: ASCO Guideline | J Clin Oncol. 2020 Dec 20;38(36):4317-4345. doi: 10.1200/JCO.20.02672. Epub 2020 Nov 16. | Gordan JD |
| 33188713 | Pattern of progression in advanced hepatocellular carcinoma treated with ramucirumab | Liver Int. 2021 Mar;41(3):598-607. doi: 10.1111/liv.14731. Epub 2020 Dec 5. | Reig M |
| 33139266 | Phase II Study of Avelumab in Patients with Advanced Hepatocellular Carcinoma Previously Treated with Sorafenib | Clin Cancer Res. 2021 Feb 1;27(3):713-718. doi: 10.1158/1078-0432.CCR-20-3094. Epub 2020 Nov 2. | Lee DW |
| 33118519 | [Effect of ozone oil for prevention and treatment of sorafenib-induced hand-foot skin reactions: a randomized controlled trial] | Nan Fang Yi Ke Da Xue Xue Bao. 2020 Oct 30;40(10):1488-1492. doi: 10.12122/j.issn.1673-4254.2020.10.15. | Chen X |
| 33048465 | Treatment strategy of adding transcatheter arterial chemoembolization to sorafenib for advanced stage hepatocellular carcinoma | Cancer Rep (Hoboken). 2021 Feb;4(1):e1294. doi: 10.1002/cnr2.1294. Epub 2020 Oct 13. | Lee WC |
| 33044793 | FDA Supplemental Approval Summary: Lenvatinib for the Treatment of Unresectable Hepatocellular Carcinoma | Oncologist. 2021 Mar;26(3):e484-e491. doi: 10.1002/onco.13566. Epub 2020 Nov 10. | Nair A |
| 33039810 | Effect of urea cream on sorafenib-associated hand-foot skin reaction in patients with hepatocellular carcinoma: A multicenter, randomised, double-blind controlled study | Eur J Cancer. 2020 Nov;140:19-27. doi: 10.1016/j.ejca.2020.09.012. Epub 2020 Oct 8. | Lee YS |
| 33001135 | Efficacy and Safety of Nivolumab Plus Ipilimumab in Patients With Advanced Hepatocellular Carcinoma Previously Treated With Sorafenib: The CheckMate 040 Randomized Clinical Trial | JAMA Oncol. 2020 Nov 1;6(11):e204564. doi: 10.1001/jamaoncol.2020.4564. Epub 2020 Nov 12. | Yau T |
| 32964588 | A clinical trial with valproic acid and hydralazine in combination with gemcitabine and cisplatin followed by doxorubicin and dacarbazine for advanced hepatocellular carcinoma | Asia Pac J Clin Oncol. 2022 Feb;18(1):19-27. doi: 10.1111/ajco.13443. Epub 2020 Sep 22. | Liu YC |
| 32847838 | Second-line cabozantinib after sorafenib treatment for advanced hepatocellular carcinoma: a subgroup analysis of the phase 3 CELESTIAL trial | ESMO Open. 2020 Aug;5(4):e000714. doi: 10.1136/esmoopen-2020-000714. | Kelley RK |
| 32841541 | Phase II trial of sorafenib and doxorubicin in patients with advanced hepatocellular carcinoma after disease progression on sorafenib | Cancer Med. 2020 Oct;9(20):7453-7459. doi: 10.1002/cam4.3389. Epub 2020 Aug 25. | El Dika I |
| 32817068 | Ramucirumab in the second-line for patients with hepatocellular carcinoma and elevated alpha-fetoprotein: patient-reported outcomes across two randomised clinical trials | ESMO Open. 2020 Aug;5(4):e000797. doi: 10.1136/esmoopen-2020-000797. | Zhu AX |
| 32776632 | A Phase I Trial of Trametinib in Combination with Sorafenib in Patients with Advanced Hepatocellular Cancer | Oncologist. 2020 Dec;25(12):e1893-e1899. doi: 10.1634/theoncologist.2020-0759. Epub 2020 Sep 14. | Kim R |
| 32716114 | A randomized, double-blind, placebo-controlled, phase 3 study of tivantinib in Japanese patients with MET-high hepatocellular carcinoma | Cancer Sci. 2020 Oct;111(10):3759-3769. doi: 10.1111/cas.14582. Epub 2020 Aug 26. | Kudo M |
| 32615109 | Liver transplantation in hepatocellular carcinoma after tumour downstaging (XXL): a randomised, controlled, phase 2b/3 trial | Lancet Oncol. 2020 Jul;21(7):947-956. doi: 10.1016/S1470-2045(20)30224-2. | Mazzaferro V |
| 32548867 | Phase Ib Study of Enzalutamide with or Without Sorafenib in Patients with Advanced Hepatocellular Carcinoma | Oncologist. 2020 Dec;25(12):e1825-e1836. doi: 10.1634/theoncologist.2020-0521. Epub 2020 Jul 2. | Harding JJ |
| 32502443 | Atezolizumab with or without bevacizumab in unresectable hepatocellular carcinoma (GO30140): an open-label, multicentre, phase 1b study | Lancet Oncol. 2020 Jun;21(6):808-820. doi: 10.1016/S1470-2045(20)30156-X. | Lee MS |
| 32442193 | Impact of direct-acting antiviral therapy for hepatitis C-related hepatocellular carcinoma | PLoS One. 2020 May 22;15(5):e0233212. doi: 10.1371/journal.pone.0233212. eCollection 2020. | Lin WC |
| 32419473 | Lenvatinib versus sorafenib for unresectable hepatocellular carcinoma: a cost-effectiveness analysis | J Comp Eff Res. 2020 Jun;9(8):553-562. doi: 10.2217/cer-2020-0041. Epub 2020 May 18. | Cai H |
| 32417993 | Effect of early adverse events resulting in sorafenib dose adjustments on survival outcomes of advanced hepatocellular carcinoma patients | Int J Clin Oncol. 2020 Sep;25(9):1672-1677. doi: 10.1007/s10147-020-01698-7. Epub 2020 May 16. | Ruanglertboon W |
| 32402160 | Atezolizumab plus Bevacizumab in Unresectable Hepatocellular Carcinoma | N Engl J Med. 2020 May 14;382(20):1894-1905. doi: 10.1056/NEJMoa1915745. | Finn RS |

| | | | |
|---|---|---|---|
| 32366155 | The value of sorafenib trough levels in patients with advanced hepatocellular carcinoma - a substudy of the SORAMIC trial | Acta Oncol. 2020 Sep;59(9):1028-1035. doi: 10.1080/0284186X.2020.1759826. Epub 2020 May 5. | Labeur TA |
| 32357963 | MTL-CEBPA, a Small Activating RNA Therapeutic Upregulating C/EBP-α, in Patients with Advanced Liver Cancer: A First-in-Human, Multicenter, Open-Label, Phase I Trial | Clin Cancer Res. 2020 Aug 1;26(15):3936-3946. doi: 10.1158/1078-0432.CCR-20-0414. Epub 2020 May 1. | Sarker D |
| 32279446 | Ramucirumab in elderly patients with hepatocellular carcinoma and elevated alpha-fetoprotein after sorafenib in REACH and REACH-2 | Liver Int. 2020 Aug;40(8):2008-2020. doi: 10.1111/liv.14462. Epub 2020 May 6. | Kudo M |
| 32271494 | A Multicenter Phase II Study of Second-Line Axitinib for Patients with Advanced Hepatocellular Carcinoma Failing First-Line Sorafenib Monotherapy | Oncologist. 2020 Sep;25(9):e1280-e1285. doi: 10.1634/theoncologist.2020-0143. Epub 2020 Apr 9. | Lin ZZ |
| 32265508 | Covariate-adjusted analysis of the Phase 3 REFLECT study of lenvatinib versus sorafenib in the treatment of unresectable hepatocellular carcinoma | Br J Cancer. 2020 Jun;122(12):1754-1759. doi: 10.1038/s41416-020-0817-7. Epub 2020 Apr 8. | Briggs A |
| 32212089 | Prognostic Significance of VEGF and HIF-1 α in Hepatocellular Carcinoma Patients Receiving Sorafenib Versus Metformin Sorafenib Combination | J Gastrointest Cancer. 2021 Mar;52(1):269-279. doi: 10.1007/s12029-020-00389-w. | El Shorbagy S |
| 32206991 | Metabolic Switch in Hepatocellular Carcinoma Patients Treated with Sorafenib: a Proof-of-Concept Trial | Mol Imaging Biol. 2020 Oct;22(5):1446-1454. doi: 10.1007/s11307-020-01489-6. | Castello A |
| 32107609 | Ramucirumab after prior sorafenib in patients with advanced hepatocellular carcinoma and elevated alpha-fetoprotein: Japanese subgroup analysis of the REACH-2 trial | J Gastroenterol. 2020 Jun;55(6):627-639. doi: 10.1007/s00535-020-01668-w. Epub 2020 Feb 27. | Kudo M |
| 32084526 | Efficacy and Safety of Liver-Directed Concurrent Chemoradiotherapy and Sequential Sorafenib for Advanced Hepatocellular Carcinoma: A Prospective Phase 2 Trial | Int J Radiat Oncol Biol Phys. 2020 May 1;107(1):106-115. doi: 10.1016/j.ijrobp.2020.01.027. Epub 2020 Feb 19. | Kim BK |
| 31860408 | Body Composition in Patients with Radioactive Iodine-Refractory, Advanced Differentiated Thyroid Cancer Treated with Sorafenib or Placebo: A Retrospective Analysis of the Phase III DECISION Trial | Thyroid. 2019 Dec;29(12):1820-1827. doi: 10.1089/thy.2018.0784. | Huillard O |
| 31856618 | Cost-effectiveness analysis of ramucirumab treatment for patients with hepatocellular carcinoma who progressed on sorafenib with α-fetoprotein concentrations of at least 400 ng/ml | J Med Econ. 2020 Apr;23(4):347-352. doi: 10.1080/13696998.2019.1707211. Epub 2020 Jan 6. | Zheng H |
| 31832851 | Sorafenib may enhance antitumour efficacy in hepatocellular carcinoma patients by modulating the proportions and functions of natural killer cells | Invest New Drugs. 2020 Oct;38(5):1247-1256. doi: 10.1007/s10637-019-00885-2. Epub 2019 Dec 13. | Hu J |
| 31801872 | Randomised, multicentre prospective trial of transarterial chemoembolisation (TACE) plus sorafenib as compared with TACE alone in patients with hepatocellular carcinoma: TACTICS trial | Gut. 2020 Aug;69(8):1492-1501. doi: 10.1136/gutjnl-2019-318934. Epub 2019 Dec 4. | Kudo M |
| 31797680 | Tumor burden and liver function in HCC patient selection for selective internal radiation therapy: SARAH post-hoc study | Future Oncol. 2020 Jan;16(1):4315-4325. doi: 10.2217/fon-2019-0658. Epub 2019 Dec 4. | Palmer DH |
| 31790344 | Pembrolizumab As Second-Line Therapy in Patients With Advanced Hepatocellular Carcinoma in KEYNOTE-240: A Randomized, Double-Blind, Phase III Trial | J Clin Oncol. 2020 Jan 20;38(3):193-202. doi: 10.1200/JCO.19.01307. Epub 2019 Dec 2. | Finn RS |
| 31739839 | Safety, pharmacokinetics and efficacy of donafenib in treating advanced hepatocellular carcinoma: report from a phase 1b trial | Pharmazie. 2019 Nov 1;74(11):688-693. doi: 10.1691/ph.2019.9626. | Liu J |
| 31720835 | REFLECT-a phase 3 trial comparing efficacy and safety of lenvatinib to sorafenib for the treatment of unresectable hepatocellular carcinoma: an analysis of Japanese subset | J Gastroenterol. 2020 Jan;55(1):113-122. doi: 10.1007/s00535-019-01642-1. Epub 2019 Nov 12. | Yamashita T |
| 31637577 | Cabozantinib exposure-response analyses of efficacy and safety in patients with advanced hepatocellular carcinoma | J Pharmacokinet Pharmacodyn. 2019 Dec;46(6):577-589. doi: 10.1007/s10928-019-09659-y. Epub 2019 Oct 21. | Nguyen L |
| 31615466 | Randomized, phase II trial of sequential hepatic arterial infusion chemotherapy and sorafenib versus sorafenib alone as initial therapy for advanced hepatocellular carcinoma: SCOOP-2 trial | BMC Cancer. 2019 Oct 15;19(1):954. doi: 10.1186/s12885-019-6198-8. | Kondo M |
| 31570105 | Circulating DNA as prognostic biomarker in patients with advanced hepatocellular carcinoma: a translational exploratory study from the SORAMIC trial | J Transl Med. 2019 Oct 1;17(1):328. doi: 10.1186/s12967-019-2079-9. | Alunni-Fabbroni M |
| 31429027 | Efficacy and Safety of Bavituximab in Combination with Sorafenib in Advanced Hepatocellular Carcinoma: A Single-Arm, Open-Label, Phase II Clinical Trial | Target Oncol. 2019 Oct;14(5):541-550. doi: 10.1007/s11523-019-00663-3. | Mokdad AA |
| 31421157 | Impact of combined selective internal radiation therapy and sorafenib on survival in advanced hepatocellular carcinoma | J Hepatol. 2019 Dec;71(6):1164-1174. doi: 10.1016/j.jhep.2019.08.006. Epub 2019 Aug 14. | Ricke J |
| 31391334 | Augmentation of IFN-γ+ CD8+ T cell responses correlates with survival of HCC patients on sorafenib therapy | JCI Insight. 2019 Aug 8;4(15):e130116. doi: 10.1172/jci.insight.130116. eCollection 2019 Aug 8. | Kalathil SG |
| 31305287 | Phase I Study of Sorafenib and Vorinostat in Advanced Hepatocellular Carcinoma | Am J Clin Oncol. 2019 Aug;42(8):649-654. doi: 10.1097/COC.0000000000000567. | Gordon SW |
| 31295152 | A Phase 2 Study of Galunisertib (TGF-β1 Receptor Type I Inhibitor) and Sorafenib in Patients With Advanced Hepatocellular Carcinoma | Clin Transl Gastroenterol. 2019 Jul;10(7):e00056. doi: 10.14309/ctg.0000000000000056. | Kelley RK |
| 31251403 | Phase 2 study of pembrolizumab and circulating biomarkers to predict anticancer response in advanced, unresectable hepatocellular carcinoma | Cancer. 2019 Oct 15;125(20):3603-3614. doi: 10.1002/cncr.32339. Epub 2019 Jun 28. | Feun LG |
| 31249394 | Urine protein:creatinine ratio vs 24-hour urine protein for proteinuria management: analysis from the phase 3 REFLECT study of lenvatinib vs sorafenib in hepatocellular carcinoma | Br J Cancer. 2019 Jul;121(3):218-221. doi: 10.1038/s41416-019-0506-6. Epub 2019 Jun 28. | Evans TRJ |
| 31185950 | A prospective clinical trial on sorafenib treatment of hepatocellular carcinoma before liver transplantation | BMC Cancer. 2019 Jun 11;19(1):568. doi: 10.1186/s12885-019-5760-8. | Eilard MS |
| 31176752 | Nivolumab in advanced hepatocellular carcinoma: Sorafenib-experienced Asian cohort analysis | J Hepatol. 2019 Sep;71(3):543-552. doi: 10.1016/j.jhep.2019.05.014. Epub 2019 Jun 7. | Yau T |
| 31125576 | Pravastatin combination with sorafenib does not improve survival in advanced hepatocellular carcinoma | J Hepatol. 2019 Sep;71(3):516-522. doi: 10.1016/j.jhep.2019.04.021. Epub 2019 May 22. | Jouve JL |
| 31070690 | Sorafenib Plus Hepatic Arterial Infusion of Oxaliplatin, Fluorouracil, and Leucovorin vs Sorafenib Alone for Hepatocellular Carcinoma With Portal Vein Invasion: A Randomized Clinical Trial | JAMA Oncol. 2019 Jul 1;5(7):953-960. doi: 10.1001/jamaoncol.2019.0250. | He M |
| 30969136 | RATIONALE 301 study: tislelizumab versus sorafenib as first-line treatment for unresectable hepatocellular carcinoma | Future Oncol. 2019 Jun;15(16):1811-1822. doi: 10.2217/fon-2019-0097. Epub 2019 Apr 10. | Qin S |
| 30963691 | Novel transforming growth factor beta receptor I kinase inhibitor galunisertib (LY2157299) in advanced hepatocellular carcinoma | Liver Int. 2019 Aug;39(8):1468-1477. doi: 10.1111/liv.14113. Epub 2019 Jun 3. | Faivre S |
| 30954567 | Doxorubicin-loaded nanoparticles for patients with advanced hepatocellular carcinoma after sorafenib treatment failure (RELIVE): a phase 3 randomised controlled trial | Lancet Gastroenterol Hepatol. 2019 Jun;4(6):454-465. doi: 10.1016/S2468-1253(19)30040-8. Epub 2019 Apr 4. | Merle P |
| 30944458 | Sorafenib alone vs. sorafenib plus GEMOX as 1(st)-line treatment for advanced HCC: the phase II randomised PRODIGE 10 trial | Br J Cancer. 2019 Apr;120(9):896-902. doi: 10.1038/s41416-019-0443-4. Epub 2019 Apr 4. | Assenat E |
| 30920122 | Exposure-Response Modeling and Simulation of Progression-Free Survival and Adverse Events of Sorafenib Treatment in Patients With Advanced Thyroid Cancer | Clin Transl Sci. 2019 Sep;12(5):459-469. doi: 10.1111/cts.12634. Epub 2019 Apr 12. | Grevel J |
| 30665869 | Ramucirumab after sorafenib in patients with advanced hepatocellular carcinoma and increased α-fetoprotein concentrations (REACH-2): a randomised, double-blind, placebo-controlled, phase 3 trial | Lancet Oncol. 2019 Feb;20(2):282-296. doi: 10.1016/S1470-2045(18)30937-9. Epub 2019 Jan 18. | Zhu AX |
| 30587616 | β-Hydroxy-β-methyl Butyrate/L-Arginine/L-Glutamine Supplementation for Preventing Hand-Foot Skin Reaction in Sorafenib for Advanced Hepatocellular Carcinoma | In Vivo. 2019 Jan-Feb;33(1):155-161. doi: 10.21873/invivo.11452. | Naganuma A |
| 30529387 | Sorafenib with or without concurrent transarterial chemoembolization in patients with advanced hepatocellular carcinoma: The phase III STAH trial | J Hepatol. 2019 Apr;70(4):684-691. doi: 10.1016/j.jhep.2018.11.029. Epub 2018 Dec 6. | Park JW |
| 30408106 | Regorafenib treatment for patients with hepatocellular carcinoma who progressed on sorafenib-A cost-effectiveness analysis | PLoS One. 2018 Nov 8;13(11):e0207132. doi: 10.1371/journal.pone.0207132. eCollection 2018. | Shlomai A |
| 30352941 | A Phase Ib, Open-Label Study of Dalantercept, an Activin Receptor-Like Kinase 1 Ligand Trap, plus Sorafenib in Advanced Hepatocellular Carcinoma | Oncologist. 2019 Feb;24(2):161-e70. doi: 10.1634/theoncologist.2018-0654. Epub 2018 Oct 23. | Abou-Alfa GK |
| 30203774 | Drug effect analysis of sorafenib combined with transcatheter arterial chemoembolization in the treatment of advanced hepatocellular carcinoma | Pak J Pharm Sci. 2018 Jul;31(4(Special)):1751-1755. | Xu X |
| 30198057 | Phase I/II study of first-line combination therapy with sorafenib plus resminostat, an oral HDAC inhibitor, versus sorafenib monotherapy for advanced hepatocellular carcinoma in east Asian patients | Invest New Drugs. 2018 Dec;36(6):1072-1084. doi: 10.1007/s10637-018-0658-x. Epub 2018 Sep 10. | Tak WY |
| 30190369 | A Phase II and Biomarker Study of Sorafenib Combined with Modified FOLFOX in Patients with Advanced Hepatocellular Carcinoma | Clin Cancer Res. 2019 Jan 1;25(1):80-89. doi: 10.1158/1078-0432.CCR-18-0847. Epub 2018 Sep 6. | Goyal L |
| 30108162 | Molecular predictors of prevention of recurrence in HCC with sorafenib as adjuvant treatment and prognostic factors in the phase 3 STORM trial | Gut. 2019 Jun;68(6):1065-1075. doi: 10.1136/gutjnl-2018-316408. Epub 2018 Aug 14. | Pinyol R |
| 29995286 | A phase 1b study of transforming growth factor-beta receptor I inhibitor galunisertib in combination with sorafenib in Japanese patients with unresectable hepatocellular carcinoma | Invest New Drugs. 2019 Feb;37(1):118-126. doi: 10.1007/s10637-018-0636-3. Epub 2018 Jul 11. | Ikeda M |
| 29982870 | Randomized, prospective, comparative study on the effects and safety of sorafenib vs. hepatic arterial infusion chemotherapy in patients with advanced hepatocellular carcinoma with portal vein tumor thrombosis | Cancer Chemother Pharmacol. 2018 Sep;82(3):469-478. doi: 10.1007/s00280-018-3638-0. Epub 2018 Jul 7. | Choi JH |
| 29972759 | Cabozantinib in Patients with Advanced and Progressing Hepatocellular Carcinoma | N Engl J Med. 2018 Jul 5;379(1):54-63. doi: 10.1056/NEJMoa1717002. | Abou-Alfa GK |
| 29950351 | Phase II Studies with Refametinib or Refametinib plus Sorafenib in Patients with RAS-Mutated Hepatocellular Carcinoma | Clin Cancer Res. 2018 Oct 1;24(19):4650-4661. doi: 10.1158/1078-0432.CCR-17-3588. Epub 2018 Jun 27. | Lim HY |
| 29948358 | Survival and tolerance to sorafenib in Child-Pugh B patients with hepatocellular carcinoma: a prospective study | Invest New Drugs. 2018 Oct;36(5):911-918. doi: 10.1007/s10637-018-0621-x. Epub 2018 Jun 13. | Leal CRG |
| 29943624 | Are we SHARP enough? The importance of adequate patient selection in sorafenib treatment for hepatocellular carcinoma | Acta Oncol. 2018 Nov;57(11):1467-1474. doi: 10.1080/0284186X.2018.1479070. Epub 2018 Jun 26. | Labeur TA |
| 29934260 | Multicenter Phase II Clinical Trial of Sorafenib Combined with Transarterial Chemoembolization for Advanced Stage Hepatocellular Carcinomas (Barcelona Clinic Liver Cancer Stage C): STAB Study | J Vasc Interv Radiol. 2018 Aug;29(8):1061-1067. doi: 10.1016/j.jvir.2018.03.020. Epub 2018 Jun 20. | Sato Y |
| 29898591 | Phase I Dose-Finding Study of OPB-111077, a Novel STAT3 Inhibitor, in Patients with Advanced Hepatocellular Carcinoma | Cancer Res Treat. 2019 Apr;51(2):510-518. doi: 10.4143/crt.2018.226. Epub 2018 Jun 13. | Yoo C |
| 29875066 | Pembrolizumab in patients with advanced hepatocellular carcinoma previously treated with sorafenib (KEYNOTE-224): a non-randomised, open-label phase 2 trial | Lancet Oncol. 2018 Jul;19(7):940-952. doi: 10.1016/S1470-2045(18)30351-6. Epub 2018 Jun 3. | Zhu AX |
| 29866075 | Hepatic arterial infusion chemotherapy followed by sorafenib in patients with advanced hepatocellular carcinoma (HICS 55): an open label, non-comparative, phase II trial | BMC Cancer. 2018 Jun 4;18(1):633. doi: 10.1186/s12885-018-4519-y. | Hatooka M |
| 29719302 | A Randomized Phase II Open-Label Multi-Institution Study of the Combination of Bevacizumab and Erlotinib Compared to Sorafenib in the First-Line Treatment of Patients with Advanced Hepatocellular Carcinoma | Oncology. 2018;94(6):329-339. doi: 10.1159/000485384. Epub 2018 May 2. | Thomas MB |
| 29710388 | Antitumor effect of sorafenib and mammalian target of rapamycin inhibitor in liver transplantation recipients with hepatocellular carcinoma recurrence | Liver Transpl. 2018 Jul;24(7):932-945. doi: 10.1002/lt.25191. | Jung DH |
| 29704513 | Outcomes of sequential treatment with sorafenib followed by regorafenib for HCC: Additional analyses from the phase III RESORCE trial | J Hepatol. 2018 Aug;69(2):353-358. doi: 10.1016/j.jhep.2018.04.010. Epub 2018 Apr 26. | Finn RS |
| 29659672 | Phase III randomized study of second line ADI-PEG 20 plus best supportive care versus placebo plus best supportive care in patients with advanced hepatocellular carcinoma | Ann Oncol. 2018 Jun 1;29(6):1402-1408. doi: 10.1093/annonc/mdy101. | Abou-Alfa GK |
| 29631810 | Sorafenib plus low-dose cisplatin and fluorouracil hepatic arterial infusion chemotherapy versus sorafenib alone in patients with advanced hepatocellular carcinoma (SILIUS): a randomised, open label, phase 3 trial | Lancet Gastroenterol Hepatol. 2018 Jun;3(6):424-432. doi: 10.1016/S2468-1253(18)30078-5. Epub 2018 Apr 7. | Kudo M |
| 29625879 | Tivantinib for second-line treatment of MET-high, advanced hepatocellular carcinoma (METIV-HCC): a final analysis of a phase 3, randomised, placebo-controlled study | Lancet Oncol. 2018 May;19(5):682-693. doi: 10.1016/S1470-2045(18)30146-3. Epub 2018 Apr 3. | Rimassa L |
| 29563636 | A multicentre, open-label, phase-I/randomised phase-II study to evaluate safety, pharmacokinetics, and efficacy of nintedanib vs. sorafenib in European patients with advanced hepatocellular carcinoma | Br J Cancer. 2018 May;118(9):1162-1168. doi: 10.1038/s41416-018-0051-8. Epub 2018 Mar 22. | Palmer DH |
| 29552783 | Sunitinib versus sorafenib plus transarterial chemoembolization for inoperable hepatocellular carcinoma patients | J BUON. 2018 Jan-Feb;23(1):193-199. | Xu Q |
| 29543938 | Efficacy and Safety of Transarterial Chemoembolization Plus External Beam Radiotherapy vs Sorafenib in Hepatocellular Carcinoma With Macroscopic Vascular Invasion: A Randomized Clinical Trial | JAMA Oncol. 2018 May 1;4(5):661-669. doi: 10.1001/jamaoncol.2017.5847. | Yoon SM |
| 29520435 | A phase I trial of escalating doses of cixutumumab (IMC-A12) and sorafenib in the treatment of advanced hepatocellular carcinoma | Cancer Chemother Pharmacol. 2018 May;81(5):957-963. doi: 10.1007/s00280-018-3553-4. Epub 2018 Mar 8. | El-Khoueiry AB |
| 29506478 | A phase II trial of S-1 and oxaliplatin in patients with advanced hepatocellular carcinoma | BMC Cancer. 2018 Mar 5;18(1):252. doi: 10.1186/s12885-018-4039-9. | Lee DW |
| 29498924 | SIRveNIB: Selective Internal Radiation Therapy Versus Sorafenib in Asia-Pacific Patients With Hepatocellular Carcinoma | J Clin Oncol. 2018 Jul 1;36(19):1913-1921. doi: 10.1200/JCO.2017.76.0892. Epub 2018 Mar 2. | Chow PKH |
| 29474553 | A multicenter Phase II study of sorafenib in Japanese patients with advanced hepatocellular carcinoma and Child Pugh A and B class | Jpn J Clin Oncol. 2018 Apr 1;48(4):317-321. doi: 10.1093/jjco/hyy010. | Suzuki E |
| 29455421 | Impact of baseline characteristics on outcomes of advanced HCC patients treated with sorafenib: a secondary analysis of a phase III study | J Cancer Res Clin Oncol. 2018 May;144(5):901-908. doi: 10.1007/s00432-018-2610-z. Epub 2018 Feb 17. | Abdel-Rahman O |
| 29433850 | Lenvatinib versus sorafenib in first-line treatment of patients with unresectable hepatocellular carcinoma: a randomised phase 3 non-inferiority trial | Lancet. 2018 Mar 24;391(10126):1163-1173. doi: 10.1016/S0140-6736(18)30207-1. | Kudo M |
| 29386313 | Benefit-Risk Summary of Regorafenib for the Treatment of Patients with Advanced Hepatocellular Carcinoma That Has Progressed on Sorafenib | Oncologist. 2018 Apr;23(4):496-500. doi: 10.1634/theoncologist.2017-0422. Epub 2018 Jan 31. | Pelosof L |
| 29327075 | Phase II Study of Sorafenib Combined with Concurrent Hepatic Arterial Infusion of Oxaliplatin, 5-Fluorouracil and Leucovorin for Unresectable Hepatocellular Carcinoma with Major Portal Vein Thrombosis | Cardiovasc Intervent Radiol. 2018 May;41(5):734-743. doi: 10.1007/s00270-017-1874-z. Epub 2018 Jan 11. | He MK |
| 29258090 | Hand-Foot Syndrome and Post-Progression Treatment Are the Good Predictors of Better Survival in Advanced Hepatocellular Carcinoma Treated with Sorafenib: A Multicenter Study | Oncology. 2017;93 Suppl 1:113-119. doi: 10.1159/000481241. Epub 2017 Dec 20. | Ogawa C |
| 29209114 | Combined endovascular brachytherapy, sorafenib, and transarterial chemobolization therapy for hepatocellular carcinoma patients with portal vein tumor thrombus | World J Gastroenterol. 2017 Nov 21;23(43):7735-7745. doi: 10.3748/wjg.v23.i43.7735. | Zhang ZH |
| 29107679 | Efficacy and safety of selective internal radiotherapy with yttrium-90 resin microspheres compared with sorafenib in locally advanced and inoperable hepatocellular carcinoma (SARAH): an open-label randomised controlled phase 3 trial | Lancet Oncol. 2017 Dec;18(12):1624-1636. doi: 10.1016/S1470-2045(17)30683-6. Epub 2017 Oct 26. | Vilgrain V |
| 29040999 | Hepatic Function during Repeated TACE Procedures and Prognosis after Introducing Sorafenib in Patients with Unresectable Hepatocellular Carcinoma: Multicenter Analysis | Dig Dis. 2017;35(6):602-610. doi: 10.1159/000480256. Epub 2017 Oct 17. | Hiraoka A |
| 29040994 | Sorafenib-Regorafenib Sequential Therapy in Advanced Hepatocellular Carcinoma: A Single-Institute Experience | Dig Dis. 2017;35(6):611-617. doi: 10.1159/000480257. Epub 2017 Oct 17. | Ueshima K |
| 28906355 | Combination of transcatheter arterial chemoembolization and interrupted dosing sorafenib improves patient survival in early-intermediate stage hepatocellular carcinoma: A post hoc analysis of the START trial | Medicine (Baltimore). 2017 Sep;96(37):e7655. doi: 10.1097/MD.0000000000007655. | Lee TY |
| 28815645 | Lenalidomide as second-line therapy for advanced hepatocellular carcinoma: exploration of biomarkers for treatment efficacy | Aliment Pharmacol Ther. 2017 Oct;46(8):722-730. doi: 10.1111/apt.14270. Epub 2017 Aug 17. | Shao YY |
| 28687627 | Phase II Trial of Sorafenib in Combination with Capecitabine in Patients with Hepatocellular Carcinoma: INST 08-20 | Oncologist. 2017 Oct;22(10):1158-e116. doi: 10.1634/theoncologist.2017-0168. Epub 2017 Jul 7. | Patt Y |
| 28687477 | Prognostic factors and predictors of sorafenib benefit in patients with hepatocellular carcinoma: Analysis of two phase III studies | J Hepatol. 2017 Nov;67(5):999-1008. doi: 10.1016/j.jhep.2017.06.026. Epub 2017 Jul 4. | Bruix J |
| 28648803 | Sorafenib in combination with transarterial chemoembolisation in patients with unresectable hepatocellular carcinoma (TACE 2): a randomised placebo-controlled, double-blind, phase 3 trial | Lancet Gastroenterol Hepatol. 2017 Aug;2(8):565-575. doi: 10.1016/S2468-1253(17)30156-5. Epub 2017 Jun 23. | Meyer T |
| 28622427 | Feasibility study of personalized peptide vaccination for hepatocellular carcinoma patients refractory to locoregional therapies | Cancer Sci. 2017 Sep;108(9):1732-1738. doi: 10.1111/cas.13301. Epub 2017 Jul 23. | Yutani S |
| 28592620 | Phase II Study of First-Line Trebananib Plus Sorafenib in Patients with Advanced Hepatocellular Carcinoma | Oncologist. 2017 Jul;22(7):780-e65. doi: 10.1634/theoncologist.2017-0058. Epub 2017 Jun 7. | Abou-Alfa GK |
| 28591675 | Ramucirumab as second-line treatment in patients with advanced hepatocellular carcinoma following first-line therapy with sorafenib: Patient-focused outcome results from the randomised phase III REACH study | Eur J Cancer. 2017 Aug;81:17-25. doi: 10.1016/j.ejca.2017.05.001. Epub 2017 Jun 4. | Chau I |
| 28573606 | A Phase I Study of Combination Therapy with Sorafenib and 5-Fluorouracil in Patients with Advanced Hepatocellular Carcinoma | Drugs R D. 2017 Sep;17(3):381-388. doi: 10.1007/s40268-017-0187-7. | Sho T |
| 28555943 | Pro-angiogenic TIE-2-expressing monocytes/TEMs as a biomarker of the effect of sorafenib in patients with advanced hepatocellular carcinoma | Int J Cancer. 2017 Sep 1;141(5):1011-1017. doi: 10.1002/ijc.30804. Epub 2017 Jun 8. | Shoji H |
| 28549676 | Exposure-response relationship of regorafenib efficacy in patients with hepatocellular carcinoma | Eur J Pharm Sci. 2017 Nov 15;109S:S149-S153. doi: 10.1016/j.ejps.2017.05.050. Epub 2017 May 23. | Solms A |
| 28497756 | S-1 versus placebo in patients with sorafenib-refractory advanced hepatocellular carcinoma (S-CUBE): a randomised, double-blind, multicentre, phase 3 trial | Lancet Gastroenterol Hepatol. 2017 Jun;2(6):407-417. doi: 10.1016/S2468-1253(17)30072-9. Epub 2017 Apr 6. | Kudo M |
| 28465443 | Phase I and Preliminary Phase II Study of TRC105 in Combination with Sorafenib in Hepatocellular Carcinoma | Clin Cancer Res. 2017 Aug 15;23(16):4633-4641. doi: 10.1158/1078-0432.CCR-16-3171. Epub 2017 May 2. | Duffy AG |
| 28434648 | Nivolumab in patients with advanced hepatocellular carcinoma (CheckMate 040): an open-label, non-comparative, phase 1/2 dose escalation and expansion trial | Lancet. 2017 Jun 24;389(10088):2492-2502. doi: 10.1016/S0140-6736(17)31046-2. Epub 2017 Apr 20. | El-Khoueiry AB |
| 28426123 | Cabozantinib in hepatocellular carcinoma: results of a phase 2 placebo-controlled randomized discontinuation study | Ann Oncol. 2017 Mar 1;28(3):528-534. doi: 10.1093/annonc/mdw651. | Kelley RK |
| 28349781 | Evaluation of sorafenib in Chinese unresectable hepatocellular carcinoma patients with prior surgery and portal vein tumor thrombosis: A subset analysis of GIDEON study data | Tumour Biol. 2017 Mar;39(3):1010428317695030. doi: 10.1177/1010428317695030. | Ye SL |
| 28341759 | A Phase Ib Study of Sorafenib (BAY 43-9006) in Patients with Kaposi Sarcoma | Oncologist. 2017 May;22(5):505-e49. doi: 10.1634/theoncologist.2016-0486. Epub 2017 Mar 24. | Uldrick TS |
| 28314990 | An apparent clinical pharmacokinetic drug-drug interaction between bevacizumab and the anti-placental growth factor monoclonal antibody RO5323441 via a target-trapping mechanism | Cancer Chemother Pharmacol. 2017 Apr;79(4):661-671. doi: 10.1007/s00280-017-3242-8. Epub 2017 Mar 17. | Wang K |
| 28279536 | Clinical characteristics of hepatocellular carcinoma in Spain. Comparison with the 2008-2009 period and analysis of the causes of diagnosis out of screening programs. Analysis of 686 cases in 73 centers | Med Clin (Barc). 2017 Jul 21;149(2):61-71. doi: 10.1016/j.medcli.2016.12.048. Epub 2017 Mar 7. | Rodríguez de Lope C |
| 28272913 | Phase I Study of Sorafenib in Combination with Intermittent Hepatic Arterial Infusion Chemotherapy for Unresectable Hepatocellular Carcinoma | Cancer Invest. 2017 Apr 21;35(4):271-276. doi: 10.1080/07357907.2017.1289382. Epub 2017 Mar 8. | Ishizaki M |
| 28152477 | Anatomic trisegmentectomy: An alternative treatment for huge or multiple hepatocellular carcinoma of right liver | Biomed Pharmacother. 2017 Apr;88:684-688. doi: 10.1016/j.biopha.2016.12.136. Epub 2017 Jan 30. | Jia C |
| 28131794 | Objective response by mRECIST as a predictor and potential surrogate end-point of overall survival in advanced HCC | J Hepatol. 2017 Jun;66(6):1166-1172. doi: 10.1016/j.jhep.2017.01.012. Epub 2017 Jan 26. | Lencioni R |
| 28120036 | Phase Ib study of codrituzumab in combination with sorafenib in patients with non-curable advanced hepatocellular carcinoma (HCC) | Cancer Chemother Pharmacol. 2017 Feb;79(2):421-429. doi: 10.1007/s00280-017-3241-9. Epub 2017 Jan 24. | Abou-Alfa GK |
| 28066978 | mRECIST to predict survival in advanced hepatocellular carcinoma: Analysis of two randomised phase II trials comparing nintedanib vs sorafenib | Liver Int. 2017 Jul;37(7):1047-1055. doi: 10.1111/liv.13359. Epub 2017 Feb 2. | Meyer T |

| | | | |
|---|---|---|---|
| 27999803 | Efficacy of External Beam Radiation-Based Treatment plus Locoregional Therapy for Hepatocellular Carcinoma Associated with Portal Vein Tumor Thrombosis | Biomed Res Int. 2016;2016:6017406. doi: 10.1155/2016/6017406. Epub 2016 Nov 24. | Chen MY |
| 27943153 | Phase I/II Randomized Trial of Sorafenib and Bevacizumab as First-Line Therapy in Patients with Locally Advanced or Metastatic Hepatocellular Carcinoma: North Central Cancer Treatment Group Trial N0745 (Alliance) | Target Oncol. 2017 Apr;12(2):201-209. doi: 10.1007/s11523-016-0467-0. | Hubbard JM |
| 27932229 | Regorafenib for patients with hepatocellular carcinoma who progressed on sorafenib treatment (RESORCE): a randomised, double-blind, placebo-controlled, phase 3 trial | Lancet. 2017 Jan 7;389(10064):56-66. doi: 10.1016/S0140-6736(16)32453-9. Epub 2016 Dec 6. | Bruix J |
| 27909950 | HATT: a phase IV, single-arm, open-label study of sorafenib in Taiwanese patients with advanced hepatocellular carcinoma | Hepatol Int. 2017 Mar;11(2):199-208. doi: 10.1007/s12072-016-9774-x. Epub 2016 Dec 1. | Lin SM |
| 27821083 | Single administration of Selective Internal Radiation Therapy versus continuous treatment with sorafeNIB in locally advanced hepatocellular carcinoma (SIRveNIB): study protocol for a phase iii randomized controlled trial | BMC Cancer. 2016 Nov 7;16(1):856. doi: 10.1186/s12885-016-2868-y. | Gandhi M |
| 27793949 | Sorafenib Combined with Radio-frequency Ablation Compared with Sorafenib Alone in Treatment of Hepatocellular Carcinoma Invading Portal Vein: A Western Randomized Controlled Trial | Anticancer Res. 2016 Nov;36(11):6179-6183. doi: 10.21873/anticanres.11211. | Giorgio A |
| 27573564 | Sorafenib plus hepatic arterial infusion chemotherapy with cisplatin versus sorafenib for advanced hepatocellular carcinoma: randomized phase II trial | Ann Oncol. 2016 Nov;27(11):2090-2096. doi: 10.1093/annonc/mdw323. Epub 2016 Aug 29. | Ikeda M |
| 27755109 | Real-life experience with sorafenib for the treatment of hepatocellular carcinoma in HIV-infected patients | AIDS. 2017 Jan 2;31(1):89-95. doi: 10.1097/QAD.0000000000001293. | Merchante N |
| 27681866 | A phase Ib study of selumetinib (AZD6244, ARRY-142886) in combination with sorafenib in advanced hepatocellular carcinoma (HCC) | Ann Oncol. 2016 Dec;27(12):2210-2215. doi: 10.1093/annonc/mdw415. Epub 2016 Sep 28. | Tai WM |
| 27627050 | Phase I study of nintedanib in Japanese patients with advanced hepatocellular carcinoma and liver impairment | Cancer Sci. 2016 Dec;107(12):1791-1799. doi: 10.1111/cas.13077. Epub 2016 Dec 12. | Okusaka T |
| 27579536 | Tumor and circulating biomarkers in patients with second-line hepatocellular carcinoma from the randomized phase II study with tivantinib | Oncotarget. 2016 Nov 8;7(45):72622-72633. doi: 10.18632/oncotarget.11621. | Rimassa L |
| 27549242 | Ramucirumab as second-line treatment in patients with advanced hepatocellular carcinoma: Japanese subgroup analysis of the REACH trial | J Gastroenterol. 2017 Apr;52(4):494-503. doi: 10.1007/s00535-016-1247-4. Epub 2016 Aug 22. | Kudo M |
| 27354623 | Pilot Study of Intrahepatic Artery Chemotherapy in Combination with Sorafenib in Hepatocellular Carcinoma | Anticancer Res. 2016 Jul;36(7):3555-63. | Dinh VY |
| 27340354 | Adjuvant sorafenib after heptectomy for Barcelona Clinic Liver Cancer-stage C hepatocellular carcinoma patients | World J Gastroenterol. 2016 Jun 21;22(23):5384-92. doi: 10.3748/wjg.v22.i23.5384. | Xia F |
| 27300260 | Bridging Sunitinib Exposure to Time-to-Tumor Progression in Hepatocellular Carcinoma Patients With Mathematical Modeling of an Angiogenic Biomarker | CPT Pharmacometrics Syst Pharmacol. 2016 Jun;5(6):297-304. doi: 10.1002/psp4.12084. Epub 2016 Jun 8. | Ait-Oudhia S |
| 27266362 | Angiogenic Response following Radioembolization: Results from a Randomized Pilot Study of Yttrium-90 with or without Sorafenib | J Vasc Interv Radiol. 2016 Sep;27(9):1329-1336. doi: 10.1016/j.jvir.2016.03.043. Epub 2016 Jun 4. | Lewandowski RJ |
| 27256874 | Phase I Study of Lenalidomide and Sorafenib in Patients With Advanced Hepatocellular Carcinoma | Oncologist. 2016 Jun;21(6):664-5. doi: 10.1634/theoncologist.2016-0071. Epub 2016 Jun 2. | Shahda S |
| 27220960 | Biomarker Analyses of Clinical Outcomes in Patients with Advanced Hepatocellular Carcinoma Treated with Sorafenib with or without Erlotinib in the SEARCH Trial | Clin Cancer Res. 2016 Oct 1;22(19):4870-4879. doi: 10.1158/1078-0432.CCR-15-2883. Epub 2016 May 24. | Zhu AX |
| 27085251 | Randomized phase II placebo controlled study of codrituzumab in previously treated patients with advanced hepatocellular carcinoma | J Hepatol. 2016 Aug;65(2):289-95. doi: 10.1016/j.jhep.2016.04.004. Epub 2016 Apr 13. | Abou-Alfa GK |
| 27082062 | Randomized, open-label phase 2 study comparing frontline dovitinib versus sorafenib in patients with advanced hepatocellular carcinoma | Hepatology. 2016 Sep;64(3):774-84. doi: 10.1002/hep.28600. Epub 2016 May 17. | Cheng AL |
| 26989044 | Concurrent sorafenib therapy extends the interval to subsequent TACE for patients with unresectable hepatocellular carcinoma | J Surg Oncol. 2016 May;113(6):672-7. doi: 10.1002/jso.24215. Epub 2016 Mar 14. | Yao X |
| 26952006 | Resminostat plus sorafenib as second-line therapy of advanced hepatocellular carcinoma - The SHELTER study | J Hepatol. 2016 Aug;65(2):280-8. doi: 10.1016/j.jhep.2016.02.043. Epub 2016 Mar 4. | Bitzer M |
| 26884590 | Sorafenib with or without everolimus in patients with advanced hepatocellular carcinoma (HCC): a randomized multicenter, multinational phase II trial (SAKK 77/08 and SASL 29) | Ann Oncol. 2016 May;27(5):856-61. doi: 10.1093/annonc/mdw054. Epub 2016 Feb 15. | Koeberle D |
| 26867886 | Phase 1 Trial of Sorafenib and Stereotactic Body Radiation Therapy for Hepatocellular Carcinoma | Int J Radiat Oncol Biol Phys. 2016 Mar 1;94(3):580-7. doi: 10.1016/j.ijrobp.2015.11.048. Epub 2015 Dec 17. | Brade AM |
| 26847681 | Antibody-Mediated Blockade of Phosphatidylserine Enhances the Antitumor Effect of Sorafenib in Hepatocellular Carcinomas Xenografts | Ann Surg Oncol. 2016 Dec;23(Suppl 5):583-591. doi: 10.1245/s10434-016-5107-5. Epub 2016 Feb 4. | Cheng X |
| 26831408 | The Prognostic Value of Alpha-Fetoprotein Response for Advanced-Stage Hepatocellular Carcinoma Treated with Sorafenib Combined with Transarterial Chemoembolization | Sci Rep. 2016 Feb 2;6:19851. doi: 10.1038/srep19851. | Liu L |
| 26809111 | Sorafenib or placebo plus TACE with doxorubicin-eluting beads for intermediate stage HCC: The SPACE trial | J Hepatol. 2016 May;64(5):1090-1098. doi: 10.1016/j.jhep.2016.01.012. Epub 2016 Jan 22. | Lencioni R |
| 26802147 | A randomized, double-blind, placebo-controlled phase II study to assess the efficacy and safety of mapatumumab with sorafenib in patients with advanced hepatocellular carcinoma | Ann Oncol. 2016 Apr;27(4):680-7. doi: 10.1093/annonc/mdw004. Epub 2016 Jan 22. | Ciuleanu T |
| 26769245 | Post-progression survival in patients with advanced hepatocellular carcinoma resistant to sorafenib | Invest New Drugs. 2016 Apr;34(2):255-60. doi: 10.1007/s10637-016-0323-1. Epub 2016 Jan 14. | Ogasawara S |
| 26735891 | Safety and efficacy of sorafenib therapy in patients with hepatocellular carcinoma: final outcome from the Chinese patient subset of the GIDEON study | Oncotarget. 2016 Feb 9;7(6):6639-48. doi: 10.18632/oncotarget.6781. | Ye SL |
| 26644411 | A Phase I Study of the Safety, Pharmacokinetics, and Pharmacodynamics of Combination Therapy with Refametinib plus Sorafenib in Patients with Advanced Cancer | Clin Cancer Res. 2016 May 15;22(10):2368-76. doi: 10.1158/1078-0432.CCR-15-1681. Epub 2015 Dec 7. | Adjei AA |
| 26462681 | Liver function assessment according to the Albumin-Bilirubin (ALBI) grade in sorafenib-treated patients with advanced hepatocellular carcinoma | Invest New Drugs. 2015 Dec;33(6):1257-62. doi: 10.1007/s10637-015-0292-9. Epub 2015 Oct 14. | Ogasawara S |
| 26449224 | Phase II study of temozolomide and veliparib combination therapy for sorafenib-refractory advanced hepatocellular carcinoma | Cancer Chemother Pharmacol. 2015 Nov;76(5):1073-9. doi: 10.1007/s00280-015-2852-2. Epub 2015 Oct 8. | Gabrielson A |
| 26446238 | Sorafenib With and Without Transarterial Chemoembolization for Advanced Hepatocellular Carcinoma With Main Portal Vein Tumor Thrombosis: A Retrospective Analysis | Oncologist. 2015 Dec;20(12):1417-24. doi: 10.1634/theoncologist.2015-0196. Epub 2015 Oct 7. | Zhang Y |
| 26421430 | Early Clinical Response after 2 Weeks of Sorafenib Therapy Predicts Outcomes and Anti-Tumor Response in Patients with Advanced Hepatocellular Carcinoma | PLoS One. 2015 Sep 30;10(9):e0138776. doi: 10.1371/journal.pone.0138776. eCollection 2015. | Kuzuya T |
| 26361969 | Adjuvant sorafenib for hepatocellular carcinoma after resection or ablation (STORM): a phase 3, randomised, double-blind, placebo-controlled trial | Lancet Oncol. 2015 Oct;16(13):1344-54. doi: 10.1016/S1470-2045(15)00198-9. Epub 2015 Sep 8. | Bruix J |
| 26330362 | Prospective analysis of tiopronin in prevention of sorafenib and antiviral therapy inducing liver toxicity in advanced hepatitis B virus-related hepatocellular carcinoma | Med Oncol. 2015 Oct;32(10):238. doi: 10.1007/s12032-015-0684-x. Epub 2015 Sep 2. | Li J |
| 26207356 | Phase 2 trial of sorafenib in children and young adults with refractory solid tumors: A report from the Children's Oncology Group | Pediatr Blood Cancer. 2015 Sep;62(9):1562-6. doi: 10.1002/pbc.25548. Epub 2015 Apr 27. | Kim A |
| 26158398 | Impact of sorafenib dosing on outcome from the European patient subset of the GIDEON study | Future Oncol. 2015 Sep;11(18):2553-62. doi: 10.2217/fon.15.163. Epub 2015 Jul 9. | Daniele B |
| 26095784 | Ramucirumab versus placebo as second-line treatment in patients with advanced hepatocellular carcinoma following first-line therapy with sorafenib (REACH): a randomised, double-blind, multicentre, phase 3 trial | Lancet Oncol. 2015 Jul;16(7):859-70. doi: 10.1016/S1470-2045(15)00050-9. Epub 2015 Jun 18. | Zhu AX |
| 26072416 | A Phase 2, Open-Label, Randomized Study of Pexa-Vec (JX-594) Administered by Intratumoral Injection in Patients with Unresectable Primary Hepatocellular Carcinoma | Methods Mol Biol. 2015;1317:343-57. doi: 10.1007/978-1-4939-2727-2_19. | Breitbach CJ |
| 26071796 | Safety and efficacy of tigatuzumab plus sorafenib as first-line therapy in subjects with advanced hepatocellular carcinoma: A phase 2 randomized study | J Hepatol. 2015 Oct;63(4):896-904. doi: 10.1016/j.jhep.2015.06.001. Epub 2015 Jun 10. | Cheng AL |
| 26069923 | Open-Label Single-Arm Phase II Trial of Sorafenib Therapy with Drug-eluting Bead Transarterial Chemoembolization in Patients with Unresectable Hepatocellular Carcinoma: Clinical Results | Radiology. 2015 Nov;277(2):594-603. doi: 10.1148/radiol.2015142481. Epub 2015 Jun 11. | Cosgrove DP |
| 25965825 | SLC15A2 genomic variation is associated with the extraordinary response of sorafenib treatment: whole-genome analysis in patients with hepatocellular carcinoma | Oncotarget. 2015 Jun 30;6(18):16449-60. doi: 10.18632/oncotarget.3758. | Lee YS |
| 25957784 | Impact of neo-adjuvant Sorafenib treatment on liver transplantation in HCC patients - a prospective, randomized, double-blind, phase III trial | BMC Cancer. 2015 May 11;15:392. doi: 10.1186/s12885-015-1373-z. | Hoffmann K |
| 25850433 | Integrated Stable Isotope Labeling by Amino Acids in Cell Culture (SILAC) and Isobaric Tags for Relative and Absolute Quantitation (iTRAQ) Quantitative Proteomic Analysis Identifies Galectin-1 as a Potential Biomarker for Predicting Sorafenib Resistance in Liver Cancer | Mol Cell Proteomics. 2015 Jun;14(6):1527-45. doi: 10.1074/mcp.M114.046417. Epub 2015 Apr 7. | Yeh CC |
| 25838254 | Tivantinib, a new option for second-line treatment of advanced hepatocellular carcinoma? The experience of Italian centers | Tumori. 2015 Mar-Apr;101(2):139-43. doi: 10.5301/tj.5000217. Epub 2015 Mar 25. | Rimassa L |
| 25750040 | Adjuvant sorafenib therapy in patients with resected hepatocellular carcinoma: evaluation of predictive factors | Med Oncol. 2015 Apr;32(4):107. doi: 10.1007/s12032-015-0549-3. Epub 2015 Mar 7. | Zhang W |
| 25711511 | Phase I study of tivantinib in Japanese patients with advanced hepatocellular carcinoma: Distinctive pharmacokinetic profiles from other solid tumors | Cancer Sci. 2015 May;106(5):611-7. doi: 10.1111/cas.12644. Epub 2015 Apr 7. | Okusaka T |
| 25683938 | Sorafenib combined with percutaneous radiofrequency ablation for the treatment of medium-sized hepatocellular carcinoma | Eur Rev Med Pharmacol Sci. 2015;19(2):247-55. | Kan X |
| 25667293 | Randomized controlled trial of the prophylactic effect of urea-based cream on sorafenib-associated hand-foot skin reactions in patients with advanced hepatocellular carcinoma | J Clin Oncol. 2015 Mar 10;33(8):894-900. doi: 10.1200/JCO.2013.52.9651. Epub 2015 Feb 9. | Ren Z |
| 25583146 | [Therapeutic decisions in the treatment of hepatocellular carcinoma and patterns of sorafenib use. Results of the international observational GIDEON trial in Spain] | Gastroenterol Hepatol. 2015 Apr;38(4):263-73. doi: 10.1016/j.gastrohep.2014.11.001. Epub 2015 Jan 9. | Turnes J |
| 25547503 | SEARCH: a phase III, randomized, double-blind, placebo-controlled trial of sorafenib plus erlotinib in patients with advanced hepatocellular carcinoma | J Clin Oncol. 2015 Feb 20;33(6):559-66. doi: 10.1200/JCO.2013.53.7746. Epub 2014 Dec 29. | Zhu AX |
| 25488963 | Linifanib versus Sorafenib in patients with advanced hepatocellular carcinoma: results of a randomized phase III trial | J Clin Oncol. 2015 Jan 10;33(2):172-9. doi: 10.1200/JCO.2013.54.3298. Epub 2014 Dec 8. | Cainap C |
| 25472660 | Radioembolisation with yttrium–90 microspheres versus sorafenib for treatment of advanced hepatocellular carcinoma (SARAH): study protocol for a randomised controlled trial | Trials. 2014 Dec 3;15:474. doi: 10.1186/1745-6215-15-474. | Vilgrain V |
| 25294897 | A phase II study of the efficacy and safety of the combination therapy of the MEK inhibitor refametinib (BAY 86-9766) plus sorafenib for Asian patients with unresectable hepatocellular carcinoma | Clin Cancer Res. 2014 Dec 1;20(23):5976-85. doi: 10.1158/1078-0432.CCR-13-3445. Epub 2014 Oct 7. | Lim HY |
| 25294187 | Phase 1 trial of tivantinib in combination with sorafenib in adult patients with advanced solid tumors | Invest New Drugs. 2015 Feb;33(1):159-68. doi: 10.1007/s10637-014-0167-5. Epub 2014 Oct 8. | Puzanov I |
| 25248753 | A phase I and pharmacokinetic study of ganetespib (STA-9090) in advanced hepatocellular carcinoma | Invest New Drugs. 2015 Feb;33(1):128-37. doi: 10.1007/s10637-014-0164-8. Epub 2014 Sep 24. | Goyal L |
| 25226843 | Volumetric assessment of tumour response using functional MR imaging in patients with hepatocellular carcinoma treated with a combination of doxorubicin-eluting beads and sorafenib | Eur Radiol. 2015 Feb;25(2):380-90. doi: 10.1007/s00330-014-3412-6. Epub 2014 Sep 17. | Corona-Villalobos CP |
| 25173458 | TACE plus sorafenib for the treatment of hepatocellular carcinoma: results of the multicenter, phase II SOCRATES trial | Cancer Chemother Pharmacol. 2014 Nov;74(5):947-54. doi: 10.1007/s00280-014-2568-8. Epub 2014 Aug 31. | Erhardt A |
| 25099027 | The combination of transcatheter arterial chemoembolization and sorafenib is well tolerated and effective in Asian patients with hepatocellular carcinoma: final results of the START trial | Int J Cancer. 2015 Mar 15;136(6):1458-67. doi: 10.1002/ijc.29126. Epub 2014 Sep 16. | Chao Y |
| 25058218 | Effect of everolimus on survival in advanced hepatocellular carcinoma after failure of sorafenib: the EVOLVE-1 randomized clinical trial | JAMA. 2014 Jul 2;312(1):57-67. doi: 10.1001/jama.2014.7189. | Zhu AX |
| 24977690 | Randomized Phase II Study of the X-linked Inhibitor of Apoptosis (XIAP) Antisense AEG35156 in Combination With Sorafenib in Patients With Advanced Hepatocellular Carcinoma (HCC) | Am J Clin Oncol. 2016 Dec;39(6):609-613. doi: 10.1097/COC.0000000000000099. | Lee FA |
| 24930619 | Safety and toxicity of radioembolization plus Sorafenib in advanced hepatocellular carcinoma: analysis of the European multicentre trial SORAMIC | Liver Int. 2015 Feb;35(2):620-6. doi: 10.1111/liv.12622. Epub 2014 Jul 8. | Ricke J |
| 24894839 | Analysis of survival factors in patients with intermediate-advanced hepatocellular carcinoma treated with transcatheter arterial chemoembolization combined with sorafenib | Clin Transl Oncol. 2014 Nov;16(11):1012-7. doi: 10.1007/s12094-014-1189-3. Epub 2014 Jun 4. | Zheng J |
| 24810940 | Efficacy and safety of sorafenib in combination with gemcitabine in patients with advanced hepatocellular carcinoma: a multicenter, open-label, single-arm phase II study | Asia Pac J Clin Oncol. 2014 Sep;10(3):255-60. doi: 10.1111/ajco.12191. Epub 2014 May 9. | Srimuninnimit V |
| 24793745 | Neutrophil-lymphocyte ratio as a predictor of outcomes for patients with hepatocellular carcinoma undergoing TAE combined with Sorafenib | Med Oncol. 2014 Jun;31(6):969. doi: 10.1007/s12032-014-0969-5. Epub 2014 May 4. | Wei K |
| 24740650 | Safety and efficacy of sorafenib in the treatment of advanced hepatocellular carcinoma: a single center experience | Med Oncol. 2014 May;31(5):948. doi: 10.1007/s12032-014-0948-x. Epub 2014 Apr 17. | Imedio ER |
| 24737402 | A phase I/II trial of capecitabine combined with peginterferon α-2a in Patients with sorafenib-refractory advanced hepatocellular carcinoma | Invest New Drugs. 2014 Aug;32(4):762-8. doi: 10.1007/s10637-014-0097-2. Epub 2014 Apr 16. | Ogasawara S |
| 24708192 | Hepatocellular carcinoma with portal vein tumor thrombus: treatment with transarterial chemoembolization combined with sorafenib--a retrospective controlled study | Radiology. 2014 Jul;272(1):284-93. doi: 10.1148/radiol.14131946. Epub 2014 Apr 6. | Zhu K |
| 24698672 | Sorafenib in liver function impaired advanced hepatocellular carcinoma | Chin Med Sci J. 2014 Mar;29(1):7-14. doi: 10.1016/s1001-9294(14)60017-1. | Ji YX |
| 24681342 | Prospective randomized pilot study of Y90+/-sorafenib as bridge to transplantation in hepatocellular carcinoma | J Hepatol. 2014 Aug;61(2):309-17. doi: 10.1016/j.jhep.2014.03.023. Epub 2014 Mar 27. | Kulik L |
| 24661657 | Phase 2 study of combined sorafenib and radiation therapy in patients with advanced hepatocellular carcinoma | Int J Radiat Oncol Biol Phys. 2014 Apr 1;88(5):1041-7. doi: 10.1016/j.ijrobp.2014.01.017. | Chen SW |
| 24614178 | Multicenter phase II study of sequential radioembolization-sorafenib therapy for inoperable hepatocellular carcinoma | PLoS One. 2014 Mar 10;9(3):e90909. doi: 10.1371/journal.pone.0090909. eCollection 2014. | Chow PK |
| 24599799 | A phase I/II study of S-1 with sorafenib in patients with advanced hepatocellular carcinoma | Invest New Drugs. 2014 Aug;32(4):723-8. doi: 10.1007/s10637-014-0077-6. Epub 2014 Mar 7. | Ooka Y |
| 24438504 | Phase I study of combination chemotherapy using sorafenib and transcatheter arterial infusion with cisplatin for advanced hepatocellular carcinoma | Cancer Sci. 2014 Mar;105(3):354-8. doi: 10.1111/cas.12353. Epub 2014 Feb 18. | Hagihara A |
| 24372624 | The prognosis and treatment outcomes of patients with recurrent hepatocellular carcinoma after liver transplantation | Clin Transplant. 2014 Jan;28(1):141-8. doi: 10.1111/ctr.12286. Epub 2013 Dec 26. | Roh YN |
| 24350564 | The feasibility of combined transcatheter arterial chemoembolization and radiotherapy for advanced hepatocellular carcinoma | Liver Int. 2014 May;34(5):795-801. doi: 10.1111/liv.12445. Epub 2014 Jan 12. | Cho JY |
| 24333135 | Efficacy and safety of sorafenib-gemcitabine combination therapy in advanced hepatocellular carcinoma: an open-label Phase II feasibility study | Hematol Oncol Stem Cell Ther. 2014 Mar;7(1):27-31. doi: 10.1016/j.hemonc.2013.11.003. Epub 2013 Dec 12. | Naqi N |
| 24312711 | Practical effect of sorafenib monotherapy on advanced hepatocellular carcinoma and portal vein tumor thrombosis | Gut Liver. 2013 Nov;7(6):696-703. doi: 10.5009/gnl.2013.7.6.696. Epub 2013 Aug 14. | Jeong SW |
| 24283303 | GIDEON (Global Investigation of therapeutic DEcisions in hepatocellular carcinoma and Of its treatment with sorafeNib): second interim analysis | Int J Clin Pract. 2014 May;68(5):609-17. doi: 10.1111/ijcp.12352. Epub 2013 Nov 28. | Lencioni R |
| 24232581 | Metronomic capecitabine in advanced hepatocellular carcinoma patients: a phase II study | Oncologist. 2013;18(12):1256-7. doi: 10.1634/theoncologist.2013-0093. Epub 2013 Nov 13. | Brandi G |
| 24122122 | Changes in plasma vascular endothelial growth factor at 8 weeks after sorafenib administration as predictors of survival for advanced hepatocellular carcinoma | Cancer. 2014 Jan 15;120(2):229-37. doi: 10.1002/cncr.28384. Epub 2013 Oct 7. | Tsuchiya K |
| 24081937 | Sunitinib versus sorafenib in advanced hepatocellular cancer: results of a randomized phase III trial | J Clin Oncol. 2013 Nov 10;31(32):4067-75. doi: 10.1200/JCO.2012.45.8372. Epub 2013 Sep 30. | Cheng AL |
| 23980090 | Brivanib in patients with advanced hepatocellular carcinoma who were intolerant to sorafenib or for whom sorafenib failed: results from the randomized phase III BRISK-PS study | J Clin Oncol. 2013 Oct 1;31(28):3509-16. doi: 10.1200/JCO.2012.47.3009. Epub 2013 Aug 26. | Llovet JM |
| 23980084 | Brivanib versus sorafenib as first-line therapy in patients with unresectable, advanced hepatocellular carcinoma: results from the randomized phase III BRISK-FL study | J Clin Oncol. 2013 Oct 1;31(28):3517-24. doi: 10.1200/JCO.2012.48.4410. Epub 2013 Aug 26. | Johnson PJ |
| 23928403 | Phase I study investigating everolimus combined with sorafenib in patients with advanced hepatocellular carcinoma | J Hepatol. 2013 Dec;59(6):1271-7. doi: 10.1016/j.jhep.2013.07.029. Epub 2013 Aug 6. | Finn RS |
| 23824645 | Sorafenib versus capecitabine in the management of advanced hepatocellular carcinoma | Med Oncol. 2013;30(3):655. doi: 10.1007/s12032-013-0655-z. Epub 2013 Jul 4. | Abdel-Rahman O |
| 23809766 | Regorafenib as second-line therapy for intermediate or advanced hepatocellular carcinoma: multicentre, open-label, phase II safety study | Eur J Cancer. 2013 Nov;49(16):3412-9. doi: 10.1016/j.ejca.2013.05.028. Epub 2013 Jun 25. | Bruix J |
| 23749944 | Phase I adjuvant trial of sorafenib in patients with hepatocellular carcinoma after orthotopic liver transplantation | Anticancer Res. 2013 Jun;33(6):2797-800. | Jia N |
| 23703789 | Radiological-pathological analysis of WHO, RECIST, EASL, mRECIST and DWI: Imaging analysis from a prospective randomized trial of Y90 ± sorafenib | Hepatology. 2013 Nov;58(5):1655-66. doi: 10.1002/hep.26487. Epub 2013 Oct 1. | Vouche M |
| 23648434 | Lenalidomide for second-line treatment of advanced hepatocellular cancer: a Brown University oncology group phase II study | Am J Clin Oncol. 2015 Feb;38(1):1-4. doi: 10.1097/COC.0b013e3182868c66. | Safran H |
| 23580239 | A phase II randomized dose escalation trial of sorafenib in patients with advanced hepatocellular carcinoma | Oncologist. 2013;18(4):379-80. doi: 10.1634/theoncologist.2012-0221. Epub 2013 Apr 11. | Rimassa L |
| 23547075 | Sequential phase I and II trials of stereotactic body radiotherapy for locally advanced hepatocellular carcinoma | J Clin Oncol. 2013 May 1;31(13):1631-9. doi: 10.1200/JCO.2012.44.1659. Epub 2013 Apr 1. | Bujold A |
| 23519998 | Temsirolimus combined with sorafenib in hepatocellular carcinoma: a phase I dose-finding trial with pharmacokinetic and biomarker correlates | Ann Oncol. 2013 Jul;24(7):1900-1907. doi: 10.1093/annonc/mdt109. Epub 2013 Mar 21. | Kelley RK |
| 23494836 | Sorafenib in patients with refractory or recurrent multiple myeloma | Hematol Oncol. 2013 Dec;31(4):197-200. doi: 10.1002/hon.2043. Epub 2013 Mar 15. | Yordanova A |
| 23469975 | Sorafenib in combination with transarterial chemoembolization in Chinese patients with hepatocellular carcinoma: a subgroup interim analysis of the START trial | Future Oncol. 2013 Mar;9(3):403-10. doi: 10.2217/fon.13.11. | Han G |
| 23431262 | Sorafenib in advanced hepatocellular carcinoma: a nationwide retrospective study of efficacy and tolerability | ScientificWorldJournal. 2013;2013:931972. doi: 10.1155/2013/931972. Epub 2013 Jan 29. | Køstner AH |
| 23410734 | A phase II study of sunitinib in advanced hepatocellular carcinoma | Dig Liver Dis. 2013 Aug;45(8):692-8. doi: 10.1016/j.dld.2013.01.002. Epub 2013 Feb 11. | Barone C |
| 23324079 | Sorafenib in combination with transarterial chemoembolization improves the survival of patients with unresectable hepatocellular carcinoma: a propensity score matching study | J Dig Dis. 2013 Apr;14(4):181-90. doi: 10.1111/1751-2980.12038. | Bai W |
| 23299770 | CF102 for the treatment of hepatocellular carcinoma: a phase I/II, open-label, dose-escalation study | Oncologist. 2013;18(1):25-6. doi: 10.1634/theoncologist.2012-0211. Epub 2013 Jan 8. | Stemmer SM |

| | | | |
|---|---|---|---|
| 23273822 | Advanced Hepatocellular Carcinoma: early evaluation of response to targeted therapy and prognostic value of Perfusion CT and Dynamic Contrast Enhanced-Ultrasound. Preliminary results | Eur J Radiol. 2013 May;82(5):e205-11. doi: 10.1016/j.ejrad.2012.12.004. Epub 2012 Dec 28. | Frampas E |
| 23263829 | Sorafenib in hepatocellular carcinoma: prospective study on adverse events, quality of life, and related feasibility under daily conditions | Med Oncol. 2013 Mar;30(1):345. doi: 10.1007/s12032-012-0345-2. Epub 2012 Dec 22. | Brunocilla PR |
| 23258770 | Early response to anti-tumoral treatment in hepatocellular carcinoma--can quantitative contrast-enhanced ultrasound predict outcome? | Ultraschall Med. 2013 Feb;34(1):38-46. doi: 10.1055/s-0032-1330387. Epub 2012 Dec 20. | Knieling F |
| 23246016 | Quantitative therapy response assessment by volumetric iodine-uptake measurement: initial experience in patients with advanced hepatocellular carcinoma treated with sorafenib | Eur J Radiol. 2013 Feb;82(2):327-34. doi: 10.1016/j.ejrad.2012.11.013. Epub 2012 Dec 12. | Dai X |
| 23182627 | Tivantinib for second-line treatment of advanced hepatocellular carcinoma: a randomised, placebo-controlled phase 2 study | Lancet Oncol. 2013 Jan;14(1):55-63. doi: 10.1016/S1470-2045(12)70490-4. Epub 2012 Nov 20. | Santoro A |
| 23129123 | Interim analysis of START: Study in Asia of the combination of TACE (transcatheter arterial chemoembolization) with sorafenib in patients with hepatocellular carcinoma trial | Int J Cancer. 2013 May 15;132(10):2448-58. doi: 10.1002/ijc.27925. Epub 2012 Nov 28. | Chung YH |
| 23079897 | Combination treatment of localized concurrent chemoradiation therapy and transarterial chemoembolization in locally advanced hepatocellular carcinoma with intrahepatic metastasis | Cancer Chemother Pharmacol. 2013 Jan;71(1):165-73. doi: 10.1007/s00280-012-1993-9. Epub 2012 Oct 19. | Park MS |
| 23041587 | Sorafenib in patients with Child-Pugh class A and B advanced hepatocellular carcinoma: a prospective feasibility analysis | Ann Oncol. 2013 Feb;24(2):406-411. doi: 10.1093/annonc/mds343. Epub 2012 Oct 5. | Pressiani T |
| 22902857 | Sorafenib-induced hepatocellular carcinoma cell death depends on reactive oxygen species production in vitro and in vivo | Mol Cancer Ther. 2012 Oct;11(10):2284-93. doi: 10.1158/1535-7163.MCT-12-0093. Epub 2012 Aug 17. | Coriat R |
| 22727733 | Efficacy and safety of sorafenib in patients with advanced hepatocellular carcinoma: subanalyses of a phase III trial | J Hepatol. 2012 Oct;57(4):821-9. doi: 10.1016/j.jhep.2012.06.014. Epub 2012 Jun 19. | Bruix J |
| 22720895 | Selective internal radiation therapy of hepatocellular carcinoma: potential hepatopulmonary shunt reduction after sorafenib administration | J Vasc Interv Radiol. 2012 Jul;23(7):949-52. doi: 10.1016/j.jvir.2012.04.007. | Theysohn JM |
| 22675707 | Intermediate and advanced hepatocellular carcinoma treated with the antiangiogenic agent sorafenib. Evaluation with unenhanced and contrast-enhanced ultrasonography | Med Ultrason. 2012 Jun;14(2):87-94. | Moschouris H |
| 22643560 | Bevacizumab and erlotinib in previously untreated inoperable and metastatic hepatocellular carcinoma | Am J Clin Oncol. 2013 Jun;36(3):254-7. doi: 10.1097/COC.0b013e318248d83f. | Govindarajan R |
| 22524575 | The efficacy of hepatic arterial infusion chemotherapy as an alternative to sorafenib in advanced hepatocellular carcinoma | Asia Pac J Clin Oncol. 2012 Jun;8(2):164-71. doi: 10.1111/j.1743-7563.2012.01543.x. | Jeong SW |
| 22477032 | Cryotherapy is associated with improved clinical outcomes of Sorafenib therapy for advanced hepatocellular carcinoma | Cell Biochem Biophys. 2012 Jun;63(2):159-69. doi: 10.1007/s12013-012-9353-2. | Yang Y |
| 22402942 | Phase II study of bevacizumab and erlotinib in the treatment of advanced hepatocellular carcinoma patients with sorafenib-refractory disease | Invest New Drugs. 2012 Dec;30(6):2384-90. doi: 10.1007/s10637-012-9808-8. Epub 2012 Mar 9. | Yau T |
| 22374331 | Plasma biomarkers as predictors of outcome in patients with advanced hepatocellular carcinoma | Clin Cancer Res. 2012 Apr 15;18(8):2290-300. doi: 10.1158/1078-0432.CCR-11-2175. Epub 2012 Feb 28. | Llovet JM |
| 22334456 | Transarterial chemoembolization plus sorafenib: a sequential therapeutic scheme for HCV-related intermediate-stage hepatocellular carcinoma: a randomized clinical trial | Oncologist. 2012;17(3):359-66. doi: 10.1634/theoncologist.2011-0313. Epub 2012 Feb 14. | Sansonno D |
| 22331734 | Hand-foot syndrome due to sorafenib in hepatocellular carcinoma treated with vitamin E without dose modification; a preliminary clinical study | J BUON. 2011 Oct-Dec;16(4):759-64. | Bozkurt Duman B |
| 22314421 | Phase II study of concurrent transarterial chemoembolization and sorafenib in patients with unresectable hepatocellular carcinoma | J Hepatol. 2012 Jun;56(6):1336-42. doi: 10.1016/j.jhep.2012.01.006. Epub 2012 Feb 4. | Park JW |
| 22245896 | Relationship between baseline hepatic status and outcome, and effect of sorafenib on liver function: SHARP trial subanalyses | J Hepatol. 2012 May;56(5):1080-1088. doi: 10.1016/j.jhep.2011.12.009. Epub 2012 Jan 13. | Raoul JL |
| 22240282 | Efficacy and safety of sorafenib in patients with advanced hepatocellular carcinoma according to baseline status: subset analyses of the phase III Sorafenib Asia-Pacific trial | Eur J Cancer. 2012 Jul;48(10):1452-65. doi: 10.1016/j.ejca.2011.12.006. Epub 2012 Jan 10. | Cheng AL |
| 22238246 | Phase II, open-label study of brivanib as second-line therapy in patients with advanced hepatocellular carcinoma | Clin Cancer Res. 2012 Apr 1;18(7):2090-8. doi: 10.1158/1078-0432.CCR-11-1991. Epub 2012 Jan 11. | Finn RS |
| 22215073 | Conventional transarterial chemoembolisation in combination with sorafenib for patients with hepatocellular carcinoma: a pilot study | Eur Radiol. 2012 Jun;22(6):1214-23. doi: 10.1007/s00330-011-2368-z. Epub 2012 Jan 4. | Sieghart W |
| 22033636 | Phase II trial of sorafenib in combination with 5-fluorouracil infusion in advanced hepatocellular carcinoma | Cancer Chemother Pharmacol. 2012 Mar;69(3):773-80. doi: 10.1007/s00280-011-1753-2. Epub 2011 Oct 28. | Petrini I |
| 22006162 | Saturable absorption of sorafenib in patients with solid tumors: a population model | Invest New Drugs. 2012 Oct;30(5):1991-2000. doi: 10.1007/s10637-011-9760-z. Epub 2011 Oct 18. | Hornecker M |
| 21986371 | High circulating endothelial progenitor levels associated with poor survival of advanced hepatocellular carcinoma patients receiving sorafenib combined with metronomic chemotherapy | Oncology. 2011;81(2):98-103. doi: 10.1159/000331684. Epub 2011 Oct 4. | Shao YY |
| 21981250 | Sorafenib in hepatocellular carcinoma - a post marketing evaluation | Immunopharmacol Immunotoxicol. 2012 Jun;34(3):419-22. doi: 10.3109/08923973.2011.617373. Epub 2011 Oct 8. | Trojniak MP |
| 21945779 | [Safety and efficacy of Sorafenib in treatment of tumor recurrence in liver transplantation recipients] | Nan Fang Yi Ke Da Xue Xue Bao. 2011 Sep;31(9):1608-10. | Li XH |
| 21941188 | A single center experience of sorafenib in advanced hepatocellular carcinoma patients: evaluation of prognostic factors | Eur J Gastroenterol Hepatol. 2011 Nov;23(12):1233-8. doi: 10.1097/MEG.0b013e32834bd2d0. | Song T |
| 21932373 | Efficacy and safety of sorafenib in combination with mammalian target of rapamycin inhibitors for recurrent hepatocellular carcinoma after liver transplantation | Liver Transpl. 2012 Jan;18(1):45-52. doi: 10.1002/lt.22434. | Gomez-Martin C |
| 21922643 | Sorafenib and cisplatin/doxorubicin (PLADO) in pediatric hepatocellular carcinoma | Pediatr Blood Cancer. 2012 Apr;58(4):539-44. doi: 10.1002/pbc.23295. Epub 2011 Sep 15. | Schmid I |
| 21911714 | Phase II trial of sorafenib combined with concurrent transarterial chemoembolization with drug-eluting beads for hepatocellular carcinoma | J Clin Oncol. 2011 Oct 20;29(30):3960-7. doi: 10.1200/JCO.2011.37.1021. Epub 2011 Sep 12. | Pawlik TM |
| 21885876 | The significance of early alpha-fetoprotein level changes in predicting clinical and survival benefits in advanced hepatocellular carcinoma patients receiving sorafenib | Oncologist. 2011;16(9):1270-9. doi: 10.1634/theoncologist.2011-0105. Epub 2011 Sep 1. | Yau T |
| 21726819 | Long-term safety and tolerability of sorafenib in patients with advanced non-small-cell lung cancer: a case-based review | Clin Lung Cancer. 2011 Jul;12(4):212-7. doi: 10.1016/j.cllc.2011.03.021. Epub 2011 Apr 28. | Adjei AA |
| 21695438 | Phase 1 trial of S-1 in combination with sorafenib for patients with advanced hepatocellular carcinoma | Invest New Drugs. 2012 Aug;30(4):1540-7. doi: 10.1007/s10637-011-9706-5. Epub 2011 Jun 22. | Lee SJ |
| 21664811 | Phase III study of sorafenib after transarterial chemoembolisation in Japanese and Korean patients with unresectable hepatocellular carcinoma | Eur J Cancer. 2011 Sep;47(14):2117-27. doi: 10.1016/j.ejca.2011.05.007. | Kudo M |
| 21594722 | PR-104 plus sorafenib in patients with advanced hepatocellular carcinoma | Cancer Chemother Pharmacol. 2011 Aug;68(2):539-45. doi: 10.1007/s00280-011-1671-3. Epub 2011 May 19. | Abou-Alfa GK |
| 21555932 | Gemcitabine and docetaxel for hepatocellular carcinoma: a phase II North Central Cancer Treatment Group clinical trial | Am J Clin Oncol. 2012 Oct;35(5):418-23. doi: 10.1097/COC.0b013e318219863b. | Alberts SR |
| 21340026 | Reversible decrease of portal venous flow in cirrhotic patients: a positive side effect of sorafenib | PLoS One. 2011 Feb 14;6(2):e16978. doi: 10.1371/journal.pone.0016978. | Coriat R |
| 21338641 | Dynamic contrast-enhanced magnetic resonance imaging biomarkers predict survival and response in hepatocellular carcinoma patients treated with sorafenib and metronomic tegafur/uracil | J Hepatol. 2011 Oct;55(4):858-65. doi: 10.1016/j.jhep.2011.01.032. Epub 2011 Feb 19. | Hsu CY |
| 21314962 | Phase i study evaluating the treatment of patients with hepatocellular carcinoma (HCC) with carbon ion radiotherapy: the PROMETHEUS-01 trial | BMC Cancer. 2011 Feb 12;11:67. doi: 10.1186/1471-2407-11-67. | Combs SE |
| 21122388 | [Clinical observation of transarterial chemoembolization combined with sorafenib for advanced hepatocellular carcinoma] | Zhonghua Zhong Liu Za Zhi. 2010 Sep;32(9):703-5. | Xu LT |
| 21081728 | Doxorubicin plus sorafenib vs doxorubicin alone in patients with advanced hepatocellular carcinoma: a randomized trial | JAMA. 2010 Nov 17;304(19):2154-60. doi: 10.1001/jama.2010.1672. | Abou-Alfa GK |
| 21036880 | Continuous administration of sorafenib in combination with transarterial chemoembolization in patients with hepatocellular carcinoma: results of a phase I study | Oncologist. 2010;15(11):1198-204. doi: 10.1634/theoncologist.2010-0180. Epub 2010 Oct 29. | Dufour JF |
| 21029658 | [Clinical analysis of the treatment:transcatheter arterial chemoembolization combined with sorafenib in advanced hepatocellular carcinoma] | Zhonghua Yi Xue Za Zhi. 2010 Aug 17;90(31):2187-92. | Li Y |
| 20717115 | Activity and safety of NGR-hTNF, a selective vascular-targeting agent, in previously treated patients with advanced hepatocellular carcinoma | Br J Cancer. 2010 Sep 7;103(6):837-44. doi: 10.1038/sj.bjc.6605858. Epub 2010 Aug 17. | Santoro A |
| 20642705 | Design and rationale for the non-interventional Global Investigation of Therapeutic DEcisions in Hepatocellular Carcinoma and Of its Treatment with Sorafenib (GIDEON) study | Int J Clin Pract. 2010 Jul;64(8):1034-41. doi: 10.1111/j.1742-1241.2010.02414.x. | Lencioni R |
| 20478057 | A phase II open label trial evaluating safety and efficacy of a telomerase peptide vaccination in patients with advanced hepatocellular carcinoma | BMC Cancer. 2010 May 17;10:209. doi: 10.1186/1471-2407-10-209. | Greten TF |
| 20424492 | A single-institute experience with sorafenib in untreated and previously treated patients with advanced hepatocellular carcinoma | Oncology. 2010;78(3-4):210-2. doi: 10.1159/000313701. Epub 2010 Apr 26. | Balsom SM |
| 20416968 | Phase II study of combining sorafenib with metronomic tegafur/uracil for advanced hepatocellular carcinoma | J Hepatol. 2010 Jul;53(1):126-31. doi: 10.1016/j.jhep.2010.01.035. Epub 2010 Mar 30. | Hsu CH |
| 20203173 | Continuous Sunitinib treatment in patients with advanced hepatocellular carcinoma: a Swiss Group for Clinical Cancer Research (SAKK) and Swiss Association for the Study of the Liver (SASL) multicenter phase II trial (SAKK 77/06) | Oncologist. 2010;15(3):285-92. doi: 10.1634/theoncologist.2009-0316. Epub 2010 Mar 4. | Koeberle D |
| 20149262 | Maintenance of Sorafenib following combined therapy of three-dimensional conformal radiation therapy/intensity-modulated radiation therapy and transcatheter arterial chemoembolization in patients with locally advanced hepatocellular carcinoma: a phase I/II study | Radiat Oncol. 2010 Feb 12;5:12. doi: 10.1186/1748-717X-5-12. | Zhao JD |
| 20041325 | Sorafenib plus octreotide is an effective and safe treatment in advanced hepatocellular carcinoma: multicenter phase II So.LAR. study | Cancer Chemother Pharmacol. 2010 Oct;66(5):837-44. doi: 10.1007/s00280-009-1226-z. Epub 2009 Dec 30. | Prete SD |
| 19943706 | New pharmacological developments in the treatment of hepatocellular cancer | Drugs. 2009;69(18):2533-40. doi: 10.2165/11530870-000000000-00000. | Gusani NJ |
| 19538872 | [Clinical observation of sorafenib monotherapy in Chinese patients with advanced hepatocellular carcinoma] | Zhonghua Zhong Liu Za Zhi. 2009 Jan;31(1):58-61. | Xu L |
| 19418937 | [Palliative management of hepatocarcinoma with sorafenib (Nexavar). Results of the SHARP study (sorafenib hepatocarcinoma assessment randomized protocol trial)] | Rev Med Liege. 2009 Mar;64(3):168-70. | Detry O |
| 19107763 | Phase 2 open-label study of single-agent sorafenib in treating advanced hepatocellular carcinoma in a hepatitis B-endemic Asian population: presence of lung metastasis predicts poor response | Cancer. 2009 Jan 15;115(2):428-36. doi: 10.1002/cncr.24029. | Yau T |
| 19101137 | Combination of sorafenib and doxorubicin in patients with advanced hepatocellular carcinoma: results from a phase I extension trial | Eur J Cancer. 2009 Mar;45(4):579-87. doi: 10.1016/j.ejca.2008.10.039. Epub 2008 Dec 26. | Richly H |
| 19095497 | Efficacy and safety of sorafenib in patients in the Asia-Pacific region with advanced hepatocellular carcinoma: a phase III randomised, double-blind, placebo-controlled trial | Lancet Oncol. 2009 Jan;10(1):25-34. doi: 10.1016/S1470-2045(08)70285-7. Epub 2008 Dec 16. | Cheng AL |
| 19036146 | Prospective, randomized, double-blind, multi-center, Phase III clinical study on transarterial chemoembolization (TACE) combined with Sorafenib versus TACE plus placebo in patients with hepatocellular cancer before liver transplantation - HeiLivCa [ISRCTN24081794] | BMC Cancer. 2008 Nov 26;8:349. doi: 10.1186/1471-2407-8-349. | Hoffmann K |
| 19032137 | Economic evaluation of sorafenib in the treatment of hepatocellular carcinoma in Canada | Curr Med Res Opin. 2008 Dec;24(12):3559-69. doi: 10.1185/03007990802563706. | Muszbek N |
| 18650514 | Sorafenib in advanced hepatocellular carcinoma | N Engl J Med. 2008 Jul 24;359(4):378-90. doi: 10.1056/NEJMoa0708857. | Llovet JM |
| 17953709 | Phase I study of sorafenib in Japanese patients with hepatocellular carcinoma | Cancer Sci. 2008 Jan;99(1):159-65. doi: 10.1111/j.1349-7006.2007.00648.x. Epub 2007 Oct 22. | Furuse J |
| 16908937 | Phase II study of sorafenib in patients with advanced hepatocellular carcinoma | J Clin Oncol. 2006 Sep 10;24(26):4293-300. doi: 10.1200/JCO.2005.01.3441. Epub 2006 Aug 14. | Abou-Alfa GK |
| 15613696 | Phase I clinical and pharmacokinetic study of the Novel Raf kinase and vascular endothelial growth factor receptor inhibitor BAY 43-9006 in patients with advanced refractory solid tumors | J Clin Oncol. 2005 Feb 10;23(5):965-72. doi: 10.1200/JCO.2005.06.124. Epub 2004 Dec 21. | Strumberg D |
